# Synthesis and crystal structure of 2-(anthracen-9-yl)-1-(*tert*-butyl­dimethyl­sil­yl)-3,6-di­hydro-1λ^4^,2λ^4^-aza­borinine

**DOI:** 10.1107/S2056989023008381

**Published:** 2023-10-10

**Authors:** Philipp J. Gliese, Yannik Appiarius, Tarek Scheele, Enno Lork, Tim Neudecker, Anne Staubitz

**Affiliations:** a University of Bremen, Institute for Organic and Analytical Chemistry, 28359 Bremen, Germany; b University of Bremen, MAPEX Center for Materials and Processes, 28359 Bremen, Germany; c University of Bremen, Institute for Physical and Theoretical Chemistry, 28359 Bremen, Germany; d University of Bremen, Institute for Inorganic Chemistry and Crystallography, 28359 Bremen, Germany; e Bremen Center for Computational Materials Science, 28359 Bremen, Germany; Katholieke Universiteit Leuven, Belgium

**Keywords:** crystal structure, 1,2-aza­borinine, boron–nitro­gen bond, boron–nitro­gen heterocycle, ring-closing metathesis, BN cyclo­hexene

## Abstract

The title compound, C_24_H_30_BNSi (**I**), is an asymmetric 1,2,3,6-tetra­hydro-1,2-aza­borinine consisting of a BN-substituted cyclo­hexa­diene analog with a *B*-anthracenyl substituent. A ring-closing metathesis with subsequent substitution of the obtained BCl 1,2-aza­borinine using anthracenyl lithium yielded the title compound **I**. The asymmetric unit (*Z* = 8) belongs to the ortho­rhom­bic space group *Pbca* and shows an elongated N—C bond compared to previously reported BN-1,4-cyclo­hexa­diene [Abbey *et al.* (2008 ▸) *J. Am. Chem. Soc.* **130**, 7250–7252]. The primarily contributing surface inter­actions are H⋯H and C⋯H/H⋯C (as elucidated by Hirshfeld surface analysis) which are dominated by van der Waals forces. Moreover, the non-aromatic BN heterocycle and the protecting group exhibit intra- and inter­molecular C—H⋯π inter­actions, respectively, with the anthracenyl substituent.

## Abstract 2

A modification of a B—Cl 1,2–aza­borinine precursor with anthracenyl lithium generates an air-stable analog of cyclo­hexa­diene. In the crystal packing, van der Waals inter­actions are dominant.

## 1. Chemical context

The formal replacement of a C–C bond with a B–N motif (BN isosterism) in six-membered rings changes their reactivity, as well as the dipole moments and electronic and optical properties (Bélanger-Chabot *et al.*, 2017 ▸; Campbell *et al.*, 2012 ▸; Appiarius *et al.*, 2023 ▸). This allows for potential applications in functionalized polymers (Thiedemann *et al.*, 2017 ▸), hydrogen-storage materials (Campbell *et al.*, 2010 ▸), pharmacology (Boknevitz *et al.*, 2019 ▸) or optoelectronics (Appiarius *et al.*, 2021 ▸; Hoffmann *et al.*, 2021*a* ▸,*b* ▸). 
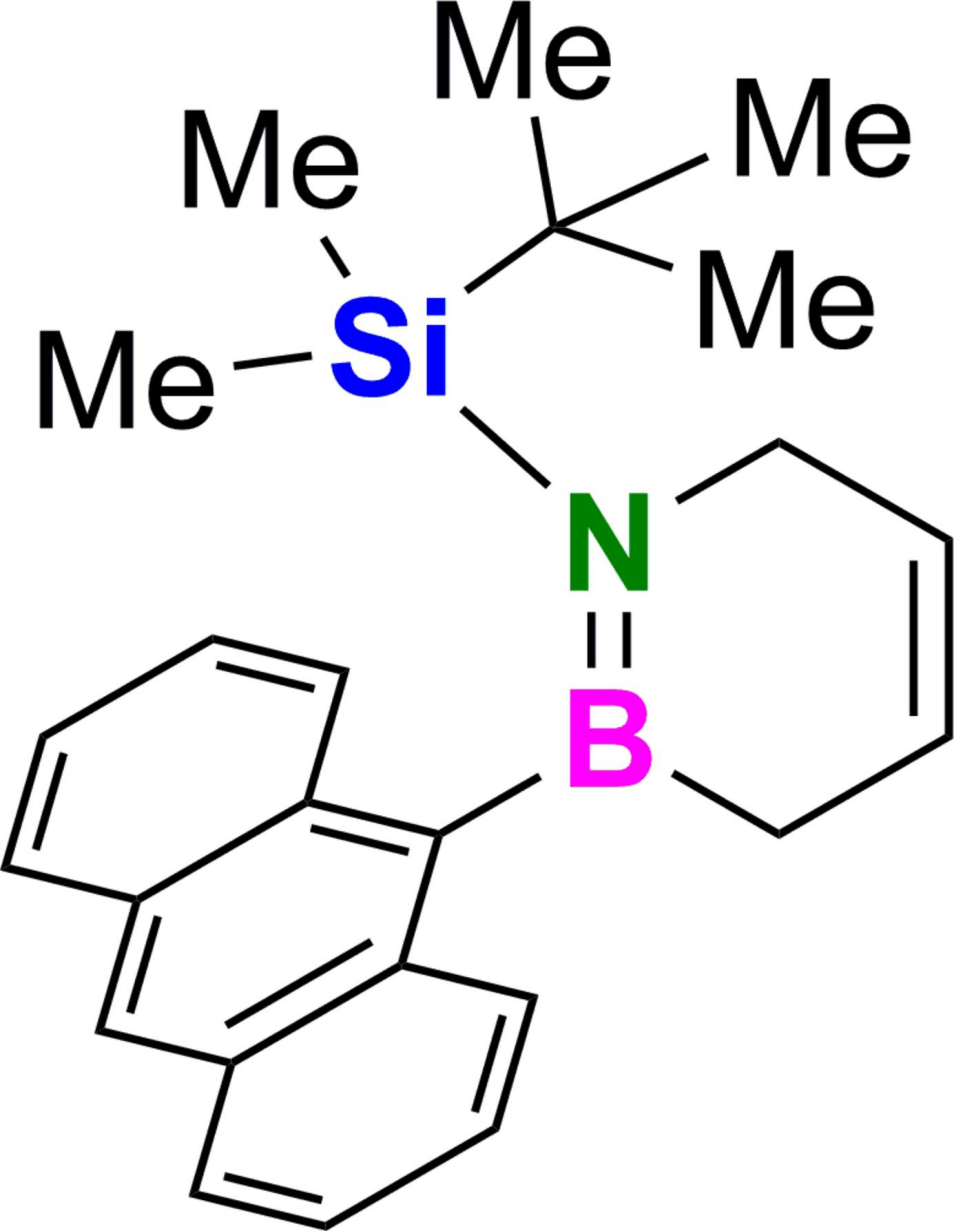


Being the formal BN analogs of 1,4-cyclo­hexa­diene, 1,2,3,6-tetra­hydro-1,2-aza­borinines were reported to be inter­mediates for the synthesis of aromatic 1,2-aza­borinines (Ashe & Fang, 2000 ▸; Marwitz *et al.*, 2009 ▸). A B-Cl 1,2,3,6-tetra­hydro-1,2-aza­borinine was aromatized under inert cond­itions, followed by subsequent substitution of the boron atom to obtain an air-stable derivative. The approach of this work presents an alternative: initial replacement of the highly reactive BCl bond by substitution with polycyclic anthracenyl lithium yields an air-stable product early on. Therefore, the respective *B*-anthracenyl heterocycle (**I**) was synthesized giving access to the 1,2,3,6-tetra­hydro-1,2-aza­borinines with lower oxidation state of the C_4_ backbone.

## 2. Structural commentary

**Figure 1:**
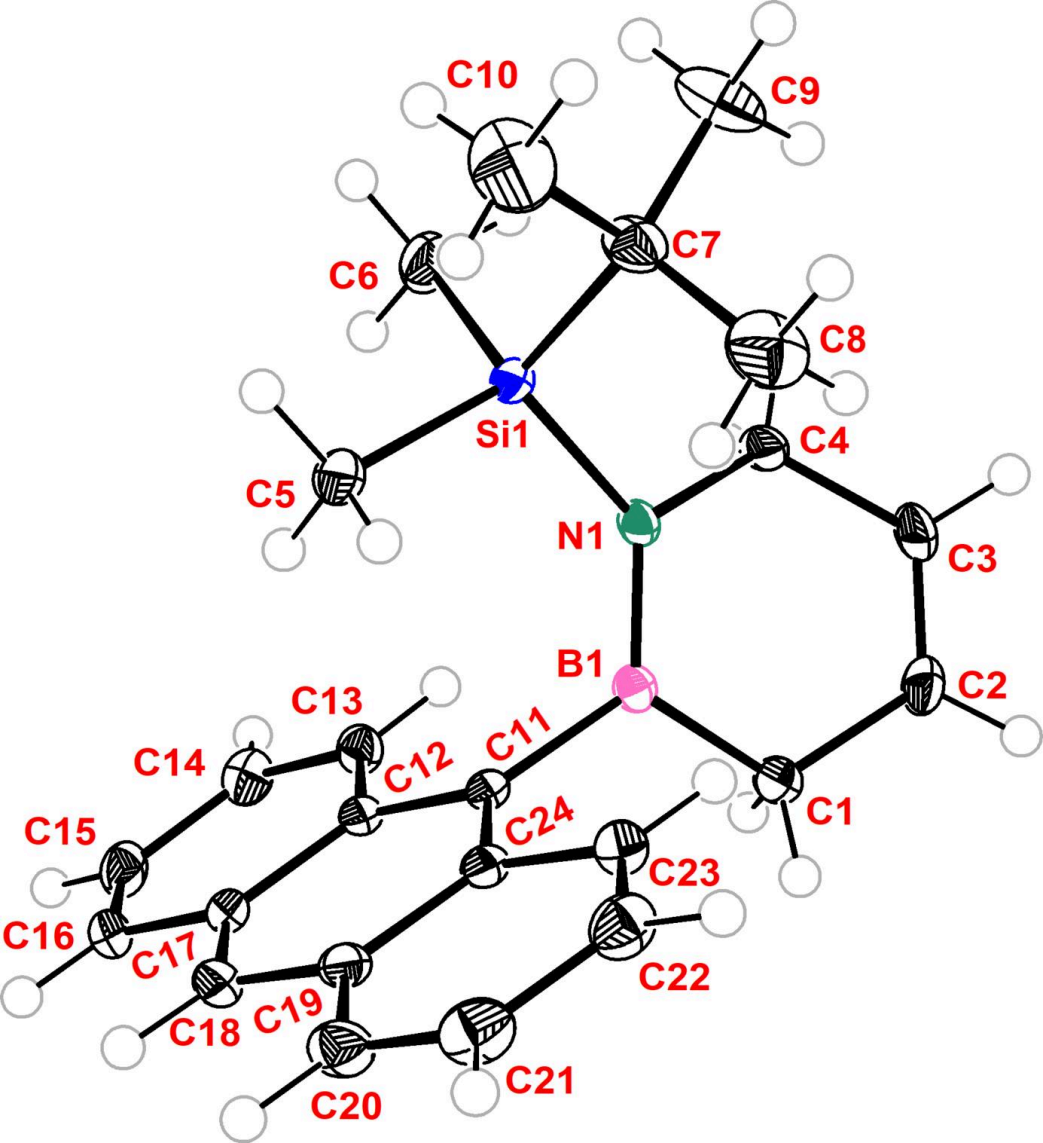
Crystal structure of the title compound **I** with atom labeling with displacement ellipsoids drawn at the 60% probability level.

The title compound **I** is an example of a 1,2,3,6-tetra­hydro-1,2-aza­borinine with substituted boron and nitro­gen atoms, crystallizing in the centrosymmetric ortho­rhom­bic space group, *Pbca* (Fig. 1 ▸). Its asymmetric unit (*Z* = 8 with *Z*′ = 1) consists of one mol­ecule. In contrast to the planar ring of the parent 1,4-cyclo­hexa­dienes (Jeffrey *et al.*, 1988 ▸), the BN-containing ring resembles a flat boat conformer. The C1—B1—N1—C4 unit is not perfectly planar [torsion angle = 10.55 (17)°] and the dihedral angle between the double-bond analog B1—N1 and the C2—C3 bond is 5.04 (11)°. Moreover, the almost perpendicular dihedral twist angle between the planar anthracenyl rings [plane of carbons C11–C24] and the B1—N1 unit is 97.96 (13)° [anthracenyl plane to B1—N1 bond angle].

According to an investigation of the B—N bond lengths in BNC_4_ rings ranging from benzene to cyclo­hexane analogs of B-NPh_2_, *N*-*tert*-butyl 1,2,3,6-tetra­hydro-1,2-aza­borinines (Abbey *et al.*, 2008 ▸), the comparison of the closely related title compound **I** with the reported 1,4-cyclo­hexa­diene analog showed comparable heterocyclic bond lengths. In the title compound **I**, the C2—C3 bond length [1.3276 (19) Å] resembles the bond in 1,4-cyclo­hexa­dienes [1.318 (2) Å] and their 1,2-aza­borinine analog [1.319 (2) Å] more closely than that in the benzene analog (Jeffrey *et al.*, 1988 ▸). Comparison of the title compound **I** with the benzene analog and three additional 1,2-aza­borinine examples (Rudebusch *et al.*, 2013 ▸; Liu *et al.*, 2021 ▸; Pan *et al.*, 2008 ▸) showed shorter B1—N1 [1.4052 (17)] and C2—C3 [1.3276 (19) Å] bond lengths. As a result of weaker bond-length compensation effects, the remaining bonds within the ring are elongated by between 0.11 and 0.15 Å.

## 3. Supra­molecular features and computational analysis

**Figure 2:**
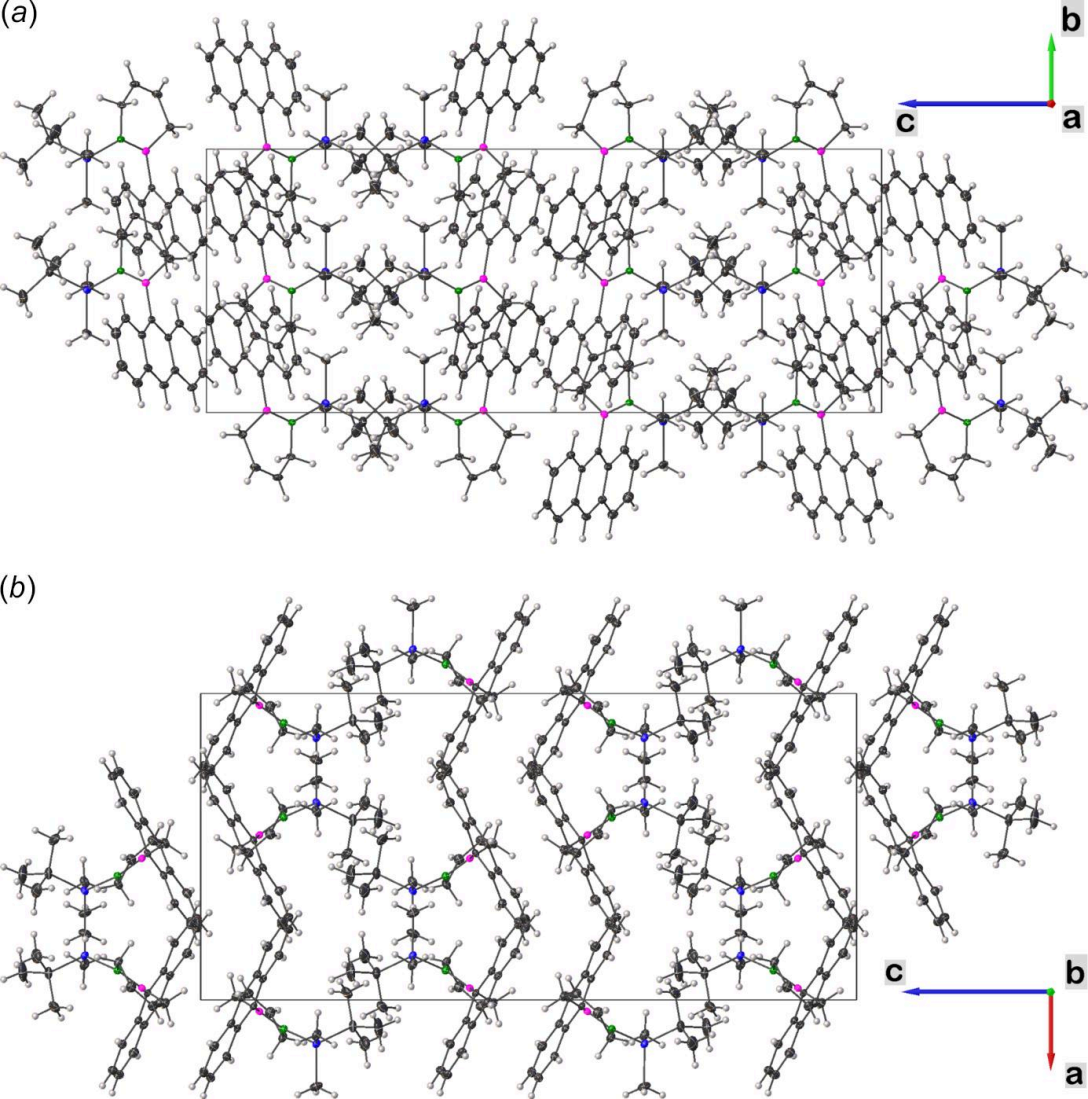
The crystal packing of the title compound **I** viewed along the *a*- (*a*) and *b*-axes (*b*). The unit cell is outlined in black.

Analysis of the crystal packing exhibits zigzag layers of the title compound **I** in the *c*-axis direction (Fig. 2 ▸). The *tert*-butyl moieties of the protecting groups are paired in groups of two along the *ac* plane (Fig. 2 ▸ *a*). The anthracenyl substituents are aligned in a zigzag manner [plane normal to plane normal angle of 52.936 (18)°], leading to a centroid–centroid distance for the anthracenyl substituents of 6.4604 (8) Å for in-plane orientation and 8.7850 (6) Å within the pattern (Fig. 2 ▸ *b*). Therefore, no π-stacking is evident, given the positioning of the anthracenyl substituents in an anti­planar arrangement.

**Figure 3:**
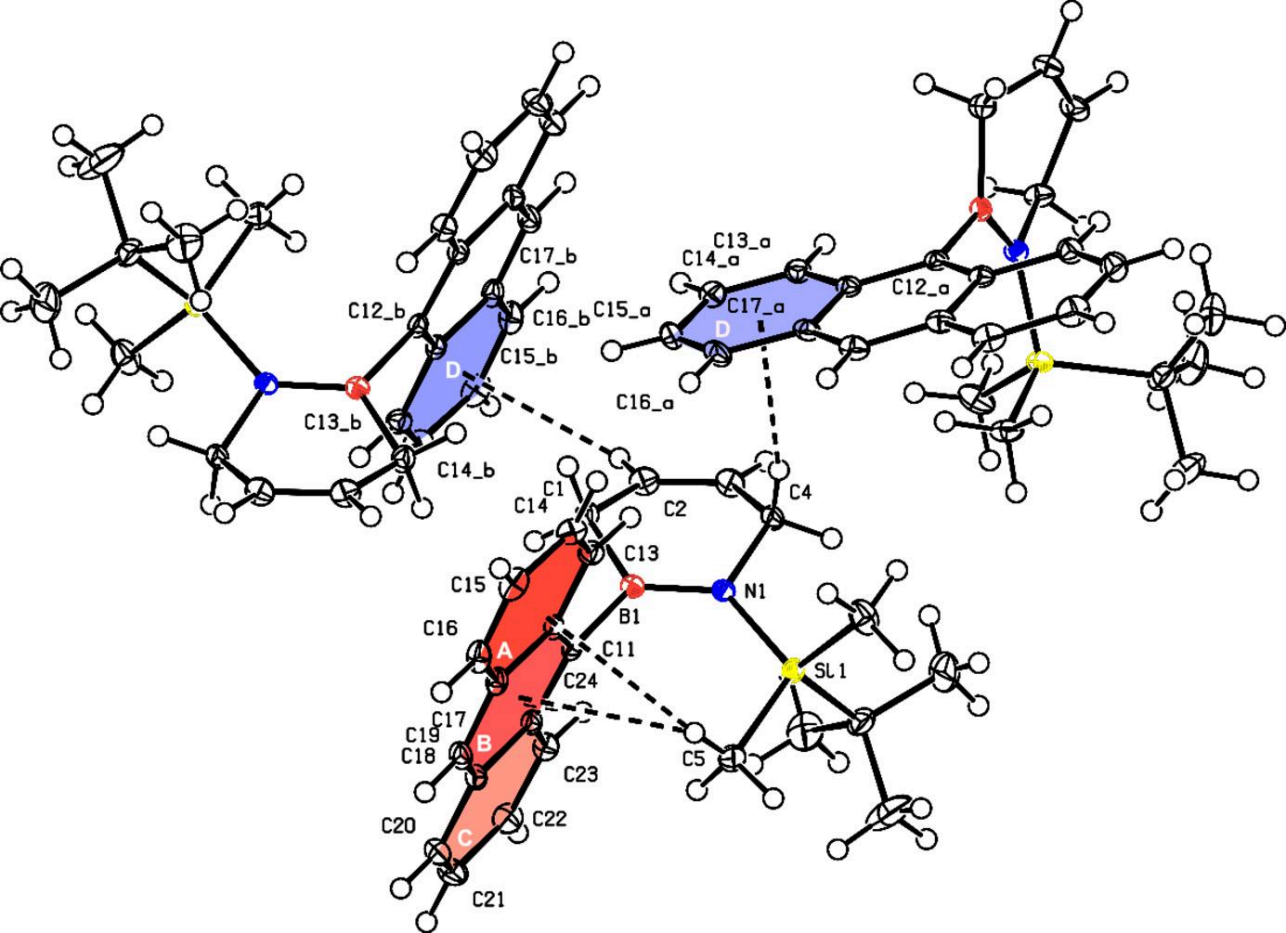
Inter- and intra­molecular C—H⋯π inter­actions in the title compound **I** with atom labeling between the respective centers of gravity (labels A–D).

The C5—H5*A* group exhibits intra­molecular C—H⋯π inter­actions with the anthracene ring [see Fig. 3 ▸ (labels A–C) and Table 1 ▸ for details; Spek, 2020 ▸). Moreover, inter­actions of the C2—H2 and the C4—H4*B* groups with the C12–C17 ring [Fig. 3 ▸ (label D), Table 1 ▸] are observed. Additional theoretical analysis (Becke, 1993 ▸; Epifanovsky *et al.*, 2021 ▸; Francl *et al.*, 1982 ▸; Glendening *et al.*, 2001 ▸; Hariharan & Pople, 1973 ▸; Hehre *et al.*, 1972 ▸; Stephens *et al.*, 1994 ▸) matching the obtained crystal bond lengths revealed no aromatic character of the BN heterocycle of the title compound **I**. Instead, the free electron pair of nitro­gen shows a significant donation to boron, and the C2—C3 bond exhibits no significant inter­actions with surrounding atoms (see supporting information). In particular, the C1—B1—N1—C4 motif shows a significant electron deficiency through low bond orders. While the B1—N1 and N1—C4 bonds have bond orders of 0.73 and 0.77, respectively, a value of 0.67 is obtained for the elongated B1—C1 bond.

## 4. Hirshfeld analysis

**Figure 4:**
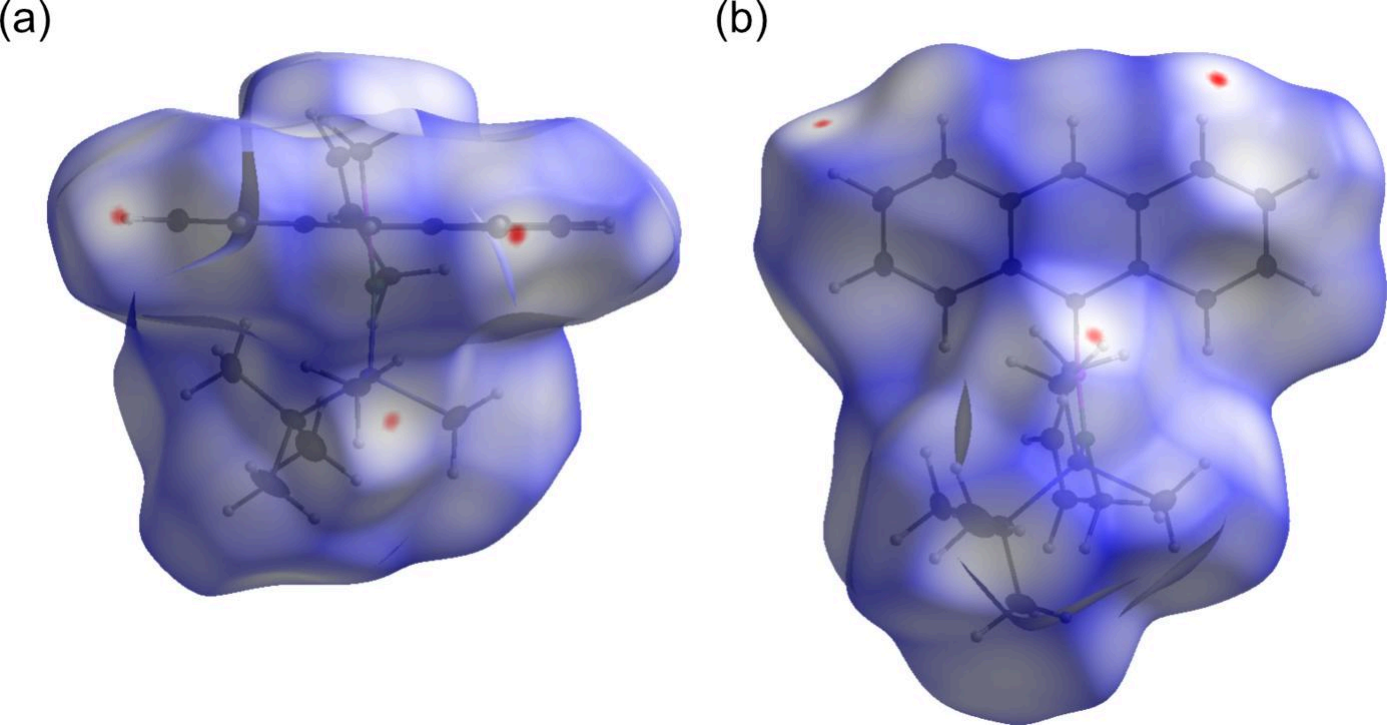
Two views of the three-dimensional Hirshfeld surface mapped over *d*_norm_.

For the analysis of the inter­molecular inter­actions, Hirshfeld surface (HS) calculations (Spackman & Jayatilaka, 2009 ▸) were performed and plotted over the *d*_norm_ in the range between −1.0432 and +2.0960 a.u. using *CrystalExplorer 21.5* (Spackman *et al.*, 2021 ▸) (Fig. 4 ▸).

Minor inter­actions were found for C14—H14, C16—H16, C21—H21, and C23—H23, as well as the two silyl methyl groups (C5 and C6).

**Figure 5:**
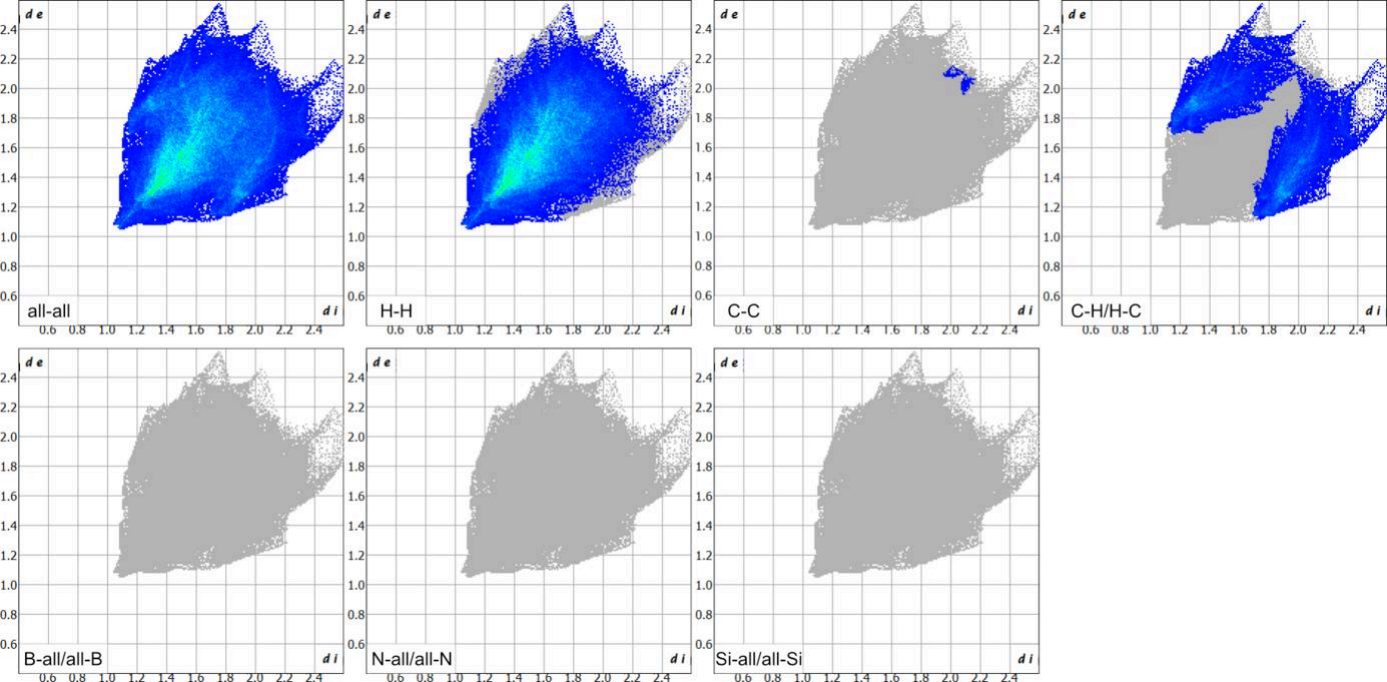
Two-dimensional fingerprint plots of title compound **I** with the respective H⋯H, C⋯C, C⋯H/H⋯C, B⋯all/all⋯B, N⋯all/all⋯N, and Si⋯all/all⋯Si inter­actions (*d*_i_ and *d*_e_ are the closest inter­nal and external distances in Å on the Hirshfeld surface).

The generation of 2D fingerprint plots (McKinnon *et al.*, 2007 ▸) was performed using CrystalExplorer 21.5, investigating all specific inter­molecular contacts (Fig. 5 ▸).

In the crystal packing, the H⋯H inter­actions are predominating and contribute to 77.0% of the overall close atom contacts (Entry 1, Table 2 ▸). C⋯C inter­actions contribute 0.1% (Entry 2, Table 2 ▸), while C⋯H/H⋯C contacts account for 22.8% (Entry 3, Table 2 ▸), indicating no additional inter­actions involving the heteroatoms.

## 5. Database Survey

A search of the Cambridge Structural Database (WebCSD version 1.9.32; update 27.06.2023; Groom *et al.*, 2016 ▸) revealed no reports of BN-containing 1,4-cyclo­hexa­diene structures with B-anthracenyl substituents. However, a search for the substructure of 1,2,3,6-tetra­hydro-1,2-aza­borinines produced nine results with the B-NPh_2_, *N*-*tert*-butyl 1,2,3,6-tetra­hydro-1,2-aza­borinine derivative (CSD refcode: EFUPIF; Abbey *et al.*, 2008 ▸) as the only monocyclic example. Of the 13 substructures with N—Si substitutions, eight examples with a similar protection group were found, but none of these examples had the C_4_ oxidation state of the title compound **I**.

## 6. Synthesis and Crystallization

The precursor, 2-chloro-1-methyl-1,2,3,6-tetra­hydro-1,2-aza­borine, was synthesized according to the literature (Appiarius *et al.*, 2021 ▸).

Under a nitro­gen atmosphere, 9-bromo­anthracene (4.68 g, 18.2 mmol) was dissolved in *n*-pentane (20 mL) and cooled to 273 K. A solution of *n*-butyl­lithium (1.1 eq., 8.0 mL, 20 mmol, 2.5 *M* in hexa­nes) was added over the course of 5 min. The solution was allowed to warm to 292 K and was stirred for 19 h. The mixture was then kept at 269 K for 48 h for subsequent precipitation. In a nitro­gen-filled glovebox, the solution was filtered through a frit (pore size 3), and the solid was washed with *n*-pentane (50 mL) until no color was observed in the filtrate. It was then transferred to a flask and dried *in vacuo* (200 mbar). The product was obtained as a yellow powder (3.32 g, 18.0 mmol, 99%, purity: 70%). The purity was determined after a literature-reported procedure (Lin & Paquette, 1994 ▸).

**Figure 6:**
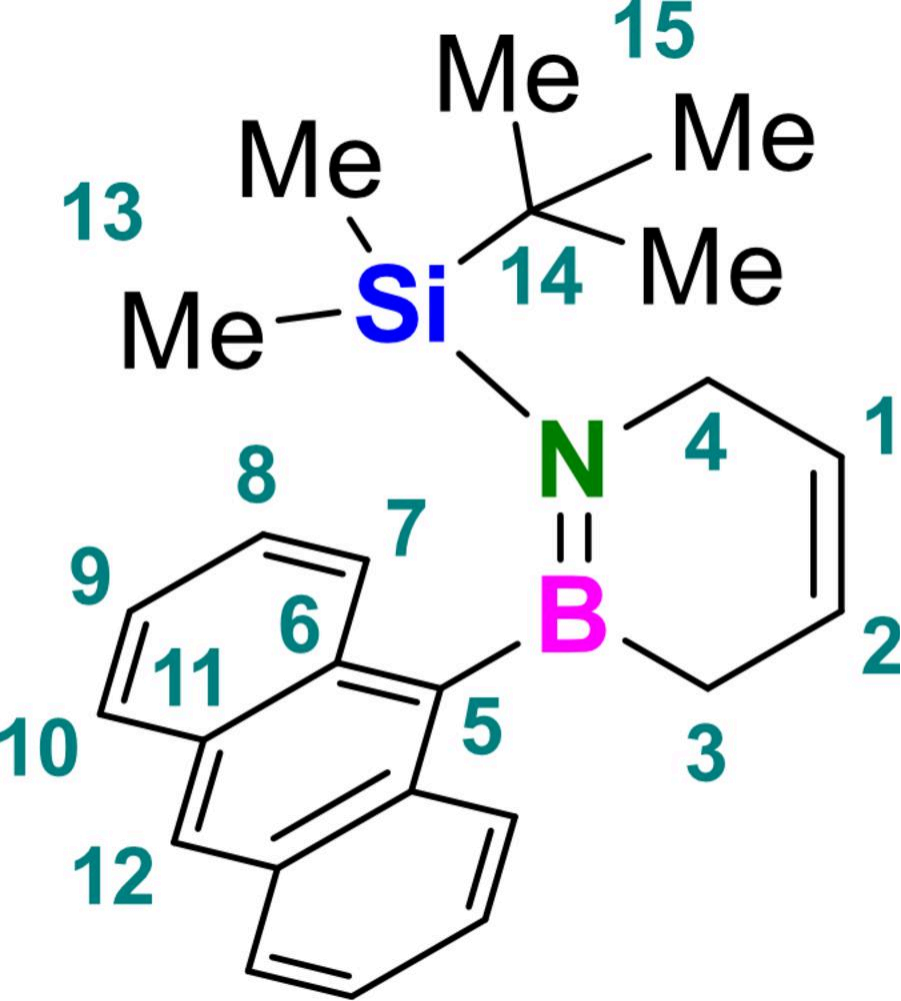
Numbering scheme of the title compound **I** used for inter­pretation of spectroscopic data.

Under a nitro­gen atmosphere, the B-Cl 1,2-aza­borinine (101 mg, 436 µmol) was dissolved in THF (5 mL) and cooled to 195 K. The lithium reagent (1.10 eq., 126 mg, 479 µmol, purity 70%) was dissolved in THF (5 mL) and added while maintaining the temperature at 195 K. The solution was allowed to warm to 292 K while it was stirred for 2 h. The reaction mixture was quenched with water (2 mL) and extracted with chloro­form (3 × 20 mL). The combined organic layers were washed with brine (2 × 20 mL) and dried over MgSO_4_. Subsequently to filtration, the solvent was removed *in vacuo* (200 mbar) to obtain the crude product. Purification by column chromatography (*n*-pentane, *R_f_* = 0.60) gave the title compound **I** as colorless crystals (129 mg, 350 µmol, 80%). The title compound was crystallized from a *n*-penta­ne/aceto­nitrile mixture by slow evaporation at 273 K. It was stored under non-inert conditions for at least 4–6 weeks (stored at 265 K) without decomposition. The numbering scheme for inter­pretation of spectroscopic data is given in Fig. 6 ▸.

^1^H NMR [600 MHz, CDCl_3_,δ (ppm)]: 8.30 (*s*, 1H, *H*-12), 7.96 (*ddd*, ^3^ *J* = 8.4 Hz, ^4^ *J* = 1.4, 0.7 Hz, 2H, *H*-10), 7.80 (*dd*, ^3^ *J* = 8.5 Hz, ^4^ *J* = 1.3, 0.7 Hz, 2H, *H*-7), 7.41 (*ddd*, ^3^ *J* = 8.4, 6.5 Hz, ^4^ *J* = 1.3 Hz, 2H, *H*-9), 7.36 (*ddd*, ^3^ *J* = 8.5, 6.5 Hz, ^4^ *J* = 1.4 Hz, 2H, *H*-8), 6.13–6.07 (*m*, 1H, *H*-2), 6.03–5.97 (*m*, 1H, *H*-1), 4.00–3.95 (*m*, 2H, *H*-4), 1.92–1.86 (*m*, 2H, *H*-3), 0.84 (*s*, 9H, *H*-15), −0.57 (*s*, 6H, *H*-13).

^13^C{^1^H} NMR [151 MHz, CDCl_3_, δ (ppm)]: 132.2 (*C*-6), 131.2 (*C*-11), 129.3 (*C*-7), 128.7 (*C*-10), 127.8 (*C*-2), 126.1 (*C*-1), 125.2 (*C*-12), 124.8 (*C*-9), 124.2 (*C*-8), 45.6 (*C*-4), 28.0 (*C*-15), 19.2 (*C*-14), 3.8 (C-13).

^11^B{^1^H} NMR [160 MHz, CDCl_3_, δ (ppm)]: 50.6.

^29^Si{^1^H} NMR [119 MHz, CDCl_3_, δ (ppm)]: 15.9.

IR [ATR, ν (cm^−1^)]: 3048 (*w*), 3024 (*w*), 2925 (*m*), 2854 (*m*), 2359 (*w*), 1621 (*w*), 1463 (*w*), 1442 (*m*), 1414 (*m*), 1378 (*m*), 1295 (*m*), 1272 (*m*), 1251 (*m*), 1123 (*m*), 1079 (*m*), 1044 (*m*), 961 (*m*), 947 (*w*), 829 (*m*), 842 (*s*), 776 (*s*), 731 (*s*), 679 (*s*).

HRMS (ESI positive, *m*/*z*): calculated for C_24_H_31_^11^BN^28^Si 372.23133 [*M* + H]^+^; found 372.23167 [*M* + H]^+^.

M.p. [DSC, Onset, (K)]: 374.

## 7. Refinement

Crystal data, data collection and structure refinement details are summarized in Table 3 ▸. Using a riding model with bond lengths of 0.95 Å (CH), 0.99 Å (CH_2_) and 0.98 Å (CH_3_), the hydrogen atoms were positioned geometrically. Isotropic displacement parameters (*U*_iso_) of these H atoms were fixed to 1.2 (CH and CH_2_) or 1.5 (CH_3_) times the values of the parent carbon atoms. The idealized methyl groups were refined as rotating groups.

## Supplementary Material

Crystal structure: contains datablock(s) I. DOI: 10.1107/S2056989023008381/vm2290sup1.cif

Structure factors: contains datablock(s) I. DOI: 10.1107/S2056989023008381/vm2290Isup3.hkl

Experimental Data, NMR Spectra, Platon Output, Output from Computational Geometry Optimization. DOI: 10.1107/S2056989023008381/vm2290sup4.pdf

Supporting information file. DOI: 10.1107/S2056989023008381/vm2290Isup4.cml

CCDC reference: 2297014

Additional supporting information:  crystallographic information; 3D view; checkCIF report

## Figures and Tables

**Table 1:** Geometry of C—H⋯π interactions (Å, °) *Cg*2, *Cg*5, *Cg*7 and *Cg*3 are the centroids of rings C11–C12/C17–C19/C24, C11–C18, C11–C24 and C12–C17, respectively.

*D*—H⋯*A*	*D*—H	H⋯*A*	*D*⋯*A*	*D*—H⋯*A*
C5—H5*A*⋯*Cg*2	0.98	2.86	3.4529 (15)	120
C5—H5*A*⋯*Cg*5	0.98	2.91	3.7305 (15)	142
C5—H5*A*⋯*Cg*7	0.98	2.88	3.4772 (15)	120
C2—H2⋯*Cg*3^i^	0.95	2.92	3.6380 (15)	133
C4—H4*B*⋯*Cg*3^ii^	0.99	2.97	3.9373 (15)	167

Symmetry codes: (i) 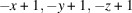 ; (ii) 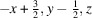 .

**Table 2:** Inter­atomic contacts with the title mol­ecule **I** as percentage contributions to the Hirshfeld surface

Entry	Contact	Percentage contribution
1	all⋯all	100
2	H⋯H	77
3	C⋯C	0.1
4	C⋯H/H⋯C	22.8
5	B⋯all/all⋯B	0
6	N⋯all/all⋯N	0
7	Si⋯all/all⋯Si	0

**Table 3:** Experimental details

Crystal data	
Chemical formula	C_24_H_30_BNSi
*M*_r_	371.39
Crystal system, space group	Orthorhombic, *P* *b* *c* *a*
Temperature (K)	100
*a*, *b*, *c* (Å)	13.3292 (6), 11.1365 (5), 28.5973 (12)
*V* (Å^3^)	4245.0 (3)
*Z*	8
Radiation type	Mo *K*α
μ (mm^−1^)	0.12
Crystal size (mm)	0.28 × 0.22 × 0.2

Data collection	
Diffractometer	Bruker Photon 100
Absorption correction	Multi-scan (*SADABS*; Krause *et al.*, 2015 ▸)
*T*_min_, *T*_max_	0.707, 0.746
No. of measured, independent and observed [*I* > 2σ(*I*)] reflections	67479, 5264, 4416
*R*_int_	0.049
(sin θ/λ)_max_ (Å^−1^)	0.667

Refinement	
*R*[*F* ^2^ > 2σ(*F* ^2^)], *wR*(*F* ^2^), *S*	0.042, 0.103, 1.07
No. of reflections	5264
No. of parameters	249
H-atom treatment	H-atom parameters constrained
Δρ_max_, Δρ_min_ (e Å^−3^)	0.37, −0.29

Computer programs: *PHOTON* and *SAINT* (Bruker, 2019 ▸), *SHELXT2014/5* (Sheldrick, 2015*a* ▸), *SHELXL* Sheldrick, 2015*b* ▸), *OLEX2* (Dolomanov *et al.*, 2009 ▸), *ORTEP-3 for Windows* (Farrugia, 2012 ▸), and *publCIF* (Westrip, (2010 ▸).

## supplementary crystallographic information

### Crystal data

**Table:**

C_24_H_30_BNSi	*D*_x_ = 1.162 Mg m^−^^3^
*M**_r_* = 371.39	Mo *K*α radiation, λ = 0.71073 Å
Orthorhombic, *P**b**c**a*	Cell parameters from 9845 reflections
*a* = 13.3292 (6) Å	θ = 2.5–30.5°
*b* = 11.1365 (5) Å	µ = 0.12 mm^−^^1^
*c* = 28.5973 (12) Å	*T* = 100 K
*V* = 4245.0 (3) Å^3^	Block, colourless
*Z* = 8	0.28 × 0.22 × 0.2 mm
*F*(000) = 1600	

### Data collection

**Table:**

Bruker Photon 100 diffractometer	5264 independent reflections
Radiation source: microfocus sealed X-ray tube, Incoatec Iµs	4416 reflections with *I* > 2σ(*I*)
Mirror optics monochromator	*R*_int_ = 0.049
Detector resolution: 7.9 pixels mm^-1^	θ_max_ = 28.3°, θ_min_ = 2.5°
ω and φ scans	*h* = −17→17
Absorption correction: multi-scan (SADABS; Krause *et al.*, 2015)	*k* = −14→14
*T*_min_ = 0.707, *T*_max_ = 0.746	*l* = −38→38
67479 measured reflections	

### Refinement

**Table:**

Refinement on *F*^2^	Primary atom site location: dual
Least-squares matrix: full	Hydrogen site location: inferred from neighbouring sites
*R*[*F*^2^ > 2σ(*F*^2^)] = 0.042	H-atom parameters constrained
*wR*(*F*^2^) = 0.103	*w* = 1/[σ^2^(*F*_o_^2^) + (0.0421*P*)^2^ + 2.6379*P*] where *P* = (*F*_o_^2^ + 2*F*_c_^2^)/3
*S* = 1.07	(Δ/σ)_max_ = 0.001
5264 reflections	Δρ_max_ = 0.37 e Å^−^^3^
249 parameters	Δρ_min_ = −0.28 e Å^−^^3^
0 restraints	

### Special details

**Table:**

Geometry. All esds (except the esd in the dihedral angle between two l.s. planes) are estimated using the full covariance matrix. The cell esds are taken into account individually in the estimation of esds in distances, angles and torsion angles; correlations between esds in cell parameters are only used when they are defined by crystal symmetry. An approximate (isotropic) treatment of cell esds is used for estimating esds involving l.s. planes.

### Fractional atomic coordinates and isotropic or equivalent isotropic displacement parameters (Å^2^)

**Table:**

	*x*	*y*	*z*	*U*_iso_*/*U*_eq_	
Si1	0.64414 (3)	0.53526 (3)	0.67676 (2)	0.01305 (9)	
N1	0.59622 (8)	0.46229 (9)	0.62665 (4)	0.0121 (2)	
C11	0.52126 (9)	0.64751 (11)	0.58088 (4)	0.0119 (2)	
C19	0.41726 (10)	0.83144 (12)	0.58633 (5)	0.0149 (3)	
C12	0.59392 (9)	0.71475 (11)	0.55610 (4)	0.0126 (2)	
C24	0.43227 (9)	0.70565 (11)	0.59506 (4)	0.0130 (2)	
C17	0.57825 (10)	0.84029 (12)	0.54660 (5)	0.0146 (3)	
C18	0.49112 (10)	0.89607 (12)	0.56257 (5)	0.0168 (3)	
H18	0.481850	0.979511	0.557198	0.020*	
C13	0.68530 (10)	0.66195 (12)	0.53987 (4)	0.0153 (3)	
H13	0.698476	0.579903	0.546572	0.018*	
C16	0.65239 (10)	0.90463 (12)	0.52065 (5)	0.0180 (3)	
H16	0.642415	0.987500	0.514292	0.022*	
C23	0.35484 (10)	0.64256 (12)	0.61956 (5)	0.0166 (3)	
H23	0.363308	0.559548	0.626116	0.020*	
C14	0.75406 (10)	0.72648 (12)	0.51500 (5)	0.0182 (3)	
H14	0.813807	0.688816	0.504372	0.022*	
C2	0.48012 (10)	0.29143 (12)	0.57271 (5)	0.0184 (3)	
H2	0.429913	0.239109	0.560836	0.022*	
C1	0.48884 (10)	0.41613 (12)	0.55382 (5)	0.0164 (3)	
H1A	0.421131	0.445468	0.545345	0.020*	
H1B	0.529719	0.414180	0.524923	0.020*	
C3	0.54188 (11)	0.25311 (12)	0.60593 (5)	0.0186 (3)	
H3	0.534485	0.173818	0.617685	0.022*	
C15	0.73675 (11)	0.84979 (13)	0.50489 (5)	0.0195 (3)	
H15	0.784345	0.893832	0.487081	0.023*	
C20	0.32658 (11)	0.88722 (13)	0.60194 (5)	0.0200 (3)	
H20	0.316206	0.970332	0.596236	0.024*	
C7	0.58707 (11)	0.46528 (13)	0.73184 (5)	0.0208 (3)	
C5	0.61517 (11)	0.69869 (12)	0.67683 (5)	0.0194 (3)	
H5A	0.639979	0.735062	0.647849	0.029*	
H5B	0.647856	0.736898	0.703685	0.029*	
H5C	0.542432	0.710205	0.679006	0.029*	
C4	0.62300 (10)	0.33186 (11)	0.62535 (5)	0.0155 (3)	
H4A	0.638945	0.304962	0.657494	0.019*	
H4B	0.684208	0.321657	0.606145	0.019*	
C22	0.26936 (10)	0.69877 (14)	0.63369 (5)	0.0207 (3)	
H22	0.218894	0.654525	0.649613	0.025*	
C21	0.25508 (11)	0.82314 (14)	0.62480 (5)	0.0221 (3)	
H21	0.195274	0.861639	0.634892	0.027*	
C6	0.78352 (10)	0.52013 (13)	0.67616 (6)	0.0236 (3)	
H6A	0.801667	0.434860	0.675946	0.035*	
H6B	0.811589	0.558499	0.704089	0.035*	
H6C	0.810525	0.559100	0.648130	0.035*	
C8	0.47669 (12)	0.43301 (16)	0.72304 (6)	0.0311 (4)	
H8A	0.440495	0.504437	0.712272	0.047*	
H8B	0.446415	0.403817	0.752130	0.047*	
H8C	0.472708	0.370187	0.699121	0.047*	
B1	0.53820 (10)	0.50755 (13)	0.58960 (5)	0.0119 (3)	
C9	0.64271 (14)	0.35359 (15)	0.74992 (6)	0.0345 (4)	
H9A	0.635604	0.288080	0.727276	0.052*	
H9B	0.614021	0.328939	0.779975	0.052*	
H9C	0.713960	0.372554	0.754027	0.052*	
C10	0.59141 (18)	0.55923 (17)	0.77124 (6)	0.0448 (5)	
H10A	0.660967	0.585254	0.775764	0.067*	
H10B	0.566082	0.523710	0.800285	0.067*	
H10C	0.549936	0.628553	0.762762	0.067*	

### Atomic displacement parameters (Å^2^)

**Table:**

	*U*^11^	*U*^22^	*U*^33^	*U*^12^	*U*^13^	*U*^23^
Si1	0.01541 (18)	0.01078 (17)	0.01295 (17)	−0.00045 (13)	−0.00323 (13)	−0.00092 (13)
N1	0.0150 (5)	0.0087 (5)	0.0127 (5)	0.0004 (4)	−0.0005 (4)	−0.0003 (4)
C11	0.0138 (6)	0.0115 (6)	0.0105 (6)	−0.0009 (5)	−0.0033 (4)	−0.0001 (5)
C19	0.0156 (6)	0.0136 (6)	0.0154 (6)	0.0021 (5)	−0.0050 (5)	−0.0010 (5)
C12	0.0149 (6)	0.0115 (6)	0.0114 (6)	−0.0008 (5)	−0.0037 (5)	0.0004 (5)
C24	0.0136 (6)	0.0130 (6)	0.0126 (6)	0.0001 (5)	−0.0036 (5)	−0.0005 (5)
C17	0.0173 (6)	0.0122 (6)	0.0144 (6)	−0.0028 (5)	−0.0054 (5)	0.0009 (5)
C18	0.0204 (7)	0.0112 (6)	0.0187 (7)	0.0011 (5)	−0.0055 (5)	0.0009 (5)
C13	0.0177 (6)	0.0132 (6)	0.0150 (6)	−0.0005 (5)	−0.0005 (5)	−0.0012 (5)
C16	0.0212 (7)	0.0133 (6)	0.0193 (7)	−0.0048 (5)	−0.0053 (5)	0.0046 (5)
C23	0.0177 (6)	0.0159 (6)	0.0162 (6)	−0.0014 (5)	−0.0013 (5)	0.0008 (5)
C14	0.0180 (6)	0.0195 (7)	0.0171 (6)	−0.0019 (5)	0.0020 (5)	−0.0027 (5)
C2	0.0200 (7)	0.0127 (6)	0.0224 (7)	−0.0042 (5)	−0.0023 (5)	−0.0027 (5)
C1	0.0204 (7)	0.0136 (6)	0.0152 (6)	−0.0005 (5)	−0.0034 (5)	0.0009 (5)
C3	0.0249 (7)	0.0088 (6)	0.0221 (7)	−0.0019 (5)	−0.0014 (5)	0.0007 (5)
C15	0.0210 (7)	0.0210 (7)	0.0164 (6)	−0.0092 (6)	0.0001 (5)	0.0030 (5)
C20	0.0209 (7)	0.0173 (7)	0.0219 (7)	0.0061 (5)	−0.0035 (5)	−0.0020 (5)
C7	0.0302 (8)	0.0196 (7)	0.0125 (6)	0.0011 (6)	0.0000 (5)	0.0005 (5)
C5	0.0262 (7)	0.0131 (6)	0.0190 (7)	−0.0001 (5)	−0.0063 (6)	−0.0036 (5)
C4	0.0204 (7)	0.0097 (6)	0.0164 (6)	0.0031 (5)	−0.0047 (5)	−0.0009 (5)
C22	0.0160 (7)	0.0259 (7)	0.0202 (7)	−0.0021 (6)	0.0014 (5)	−0.0003 (6)
C21	0.0168 (7)	0.0256 (7)	0.0238 (7)	0.0059 (6)	0.0002 (6)	−0.0042 (6)
C6	0.0174 (7)	0.0185 (7)	0.0349 (8)	−0.0013 (5)	−0.0076 (6)	0.0005 (6)
C8	0.0264 (8)	0.0412 (10)	0.0256 (8)	−0.0016 (7)	0.0092 (6)	0.0085 (7)
B1	0.0116 (6)	0.0116 (6)	0.0124 (6)	−0.0003 (5)	0.0016 (5)	0.0005 (5)
C9	0.0447 (10)	0.0350 (9)	0.0238 (8)	0.0081 (8)	0.0028 (7)	0.0144 (7)
C10	0.0817 (15)	0.0363 (10)	0.0165 (8)	−0.0084 (10)	0.0059 (9)	−0.0064 (7)

### Geometric parameters (Å, º)

**Table:**

Si1—N1	1.7669 (11)	C1—H1B	0.9900
Si1—C7	1.9148 (14)	C1—B1	1.5863 (19)
Si1—C5	1.8606 (14)	C3—H3	0.9500
Si1—C6	1.8655 (15)	C3—C4	1.4988 (18)
N1—C4	1.4963 (16)	C15—H15	0.9500
N1—B1	1.4052 (17)	C20—H20	0.9500
C11—C12	1.4144 (17)	C20—C21	1.358 (2)
C11—C24	1.4108 (18)	C7—C8	1.535 (2)
C11—B1	1.5946 (18)	C7—C9	1.538 (2)
C19—C24	1.4370 (18)	C7—C10	1.539 (2)
C19—C18	1.3960 (19)	C5—H5A	0.9800
C19—C20	1.4304 (19)	C5—H5B	0.9800
C12—C17	1.4395 (18)	C5—H5C	0.9800
C12—C13	1.4300 (18)	C4—H4A	0.9900
C24—C23	1.4318 (18)	C4—H4B	0.9900
C17—C18	1.3940 (19)	C22—H22	0.9500
C17—C16	1.4286 (19)	C22—C21	1.421 (2)
C18—H18	0.9500	C21—H21	0.9500
C13—H13	0.9500	C6—H6A	0.9800
C13—C14	1.3646 (19)	C6—H6B	0.9800
C16—H16	0.9500	C6—H6C	0.9800
C16—C15	1.357 (2)	C8—H8A	0.9800
C23—H23	0.9500	C8—H8B	0.9800
C23—C22	1.3613 (19)	C8—H8C	0.9800
C14—H14	0.9500	C9—H9A	0.9800
C14—C15	1.422 (2)	C9—H9B	0.9800
C2—H2	0.9500	C9—H9C	0.9800
C2—C1	1.4947 (18)	C10—H10A	0.9800
C2—C3	1.3276 (19)	C10—H10B	0.9800
C1—H1A	0.9900	C10—H10C	0.9800
			
N1—Si1—C7	109.65 (6)	C19—C20—H20	119.5
N1—Si1—C5	112.07 (6)	C21—C20—C19	121.00 (13)
N1—Si1—C6	108.12 (6)	C21—C20—H20	119.5
C5—Si1—C7	108.35 (7)	C8—C7—Si1	109.95 (10)
C5—Si1—C6	107.16 (7)	C8—C7—C9	109.14 (13)
C6—Si1—C7	111.50 (7)	C8—C7—C10	108.36 (14)
C4—N1—Si1	112.36 (8)	C9—C7—Si1	114.47 (11)
B1—N1—Si1	130.21 (9)	C9—C7—C10	106.60 (14)
B1—N1—C4	117.43 (10)	C10—C7—Si1	108.10 (11)
C12—C11—B1	119.92 (11)	Si1—C5—H5A	109.5
C24—C11—C12	118.47 (11)	Si1—C5—H5B	109.5
C24—C11—B1	121.51 (11)	Si1—C5—H5C	109.5
C18—C19—C24	119.27 (12)	H5A—C5—H5B	109.5
C18—C19—C20	121.60 (12)	H5A—C5—H5C	109.5
C20—C19—C24	119.13 (12)	H5B—C5—H5C	109.5
C11—C12—C17	120.62 (12)	N1—C4—C3	113.90 (11)
C11—C12—C13	121.88 (11)	N1—C4—H4A	108.8
C13—C12—C17	117.49 (12)	N1—C4—H4B	108.8
C11—C24—C19	120.97 (12)	C3—C4—H4A	108.8
C11—C24—C23	121.44 (12)	C3—C4—H4B	108.8
C23—C24—C19	117.58 (12)	H4A—C4—H4B	107.7
C18—C17—C12	119.46 (12)	C23—C22—H22	119.8
C18—C17—C16	121.54 (12)	C23—C22—C21	120.48 (13)
C16—C17—C12	119.00 (12)	C21—C22—H22	119.8
C19—C18—H18	119.4	C20—C21—C22	120.28 (13)
C17—C18—C19	121.15 (12)	C20—C21—H21	119.9
C17—C18—H18	119.4	C22—C21—H21	119.9
C12—C13—H13	119.2	Si1—C6—H6A	109.5
C14—C13—C12	121.65 (12)	Si1—C6—H6B	109.5
C14—C13—H13	119.2	Si1—C6—H6C	109.5
C17—C16—H16	119.3	H6A—C6—H6B	109.5
C15—C16—C17	121.34 (12)	H6A—C6—H6C	109.5
C15—C16—H16	119.3	H6B—C6—H6C	109.5
C24—C23—H23	119.2	C7—C8—H8A	109.5
C22—C23—C24	121.53 (13)	C7—C8—H8B	109.5
C22—C23—H23	119.2	C7—C8—H8C	109.5
C13—C14—H14	119.8	H8A—C8—H8B	109.5
C13—C14—C15	120.36 (13)	H8A—C8—H8C	109.5
C15—C14—H14	119.8	H8B—C8—H8C	109.5
C1—C2—H2	119.7	N1—B1—C11	123.12 (11)
C3—C2—H2	119.7	N1—B1—C1	118.97 (11)
C3—C2—C1	120.60 (12)	C1—B1—C11	117.89 (11)
C2—C1—H1A	108.9	C7—C9—H9A	109.5
C2—C1—H1B	108.9	C7—C9—H9B	109.5
C2—C1—B1	113.30 (11)	C7—C9—H9C	109.5
H1A—C1—H1B	107.7	H9A—C9—H9B	109.5
B1—C1—H1A	108.9	H9A—C9—H9C	109.5
B1—C1—H1B	108.9	H9B—C9—H9C	109.5
C2—C3—H3	119.2	C7—C10—H10A	109.5
C2—C3—C4	121.63 (12)	C7—C10—H10B	109.5
C4—C3—H3	119.2	C7—C10—H10C	109.5
C16—C15—C14	120.11 (13)	H10A—C10—H10B	109.5
C16—C15—H15	119.9	H10A—C10—H10C	109.5
C14—C15—H15	119.9	H10B—C10—H10C	109.5
			
Si1—N1—C4—C3	143.11 (10)	C18—C17—C16—C15	−179.54 (13)
Si1—N1—B1—C11	12.58 (19)	C13—C12—C17—C18	−178.75 (12)
Si1—N1—B1—C1	−168.86 (9)	C13—C12—C17—C16	1.69 (18)
C11—C12—C17—C18	0.84 (18)	C13—C14—C15—C16	1.1 (2)
C11—C12—C17—C16	−178.72 (12)	C16—C17—C18—C19	177.47 (12)
C11—C12—C13—C14	178.38 (12)	C23—C22—C21—C20	0.2 (2)
C11—C24—C23—C22	179.33 (13)	C2—C1—B1—N1	20.11 (17)
C19—C24—C23—C22	0.77 (19)	C2—C1—B1—C11	−161.25 (12)
C19—C20—C21—C22	0.1 (2)	C2—C3—C4—N1	32.76 (19)
C12—C11—C24—C19	−2.54 (18)	C1—C2—C3—C4	−0.7 (2)
C12—C11—C24—C23	178.94 (12)	C3—C2—C1—B1	−25.46 (19)
C12—C11—B1—N1	83.53 (16)	C20—C19—C24—C11	−179.05 (12)
C12—C11—B1—C1	−95.05 (14)	C20—C19—C24—C23	−0.48 (18)
C12—C17—C18—C19	−2.07 (19)	C20—C19—C18—C17	−178.58 (13)
C12—C17—C16—C15	0.0 (2)	C7—Si1—N1—C4	−62.08 (10)
C12—C13—C14—C15	0.7 (2)	C7—Si1—N1—B1	117.35 (12)
C24—C11—C12—C17	1.44 (18)	C5—Si1—N1—C4	177.55 (9)
C24—C11—C12—C13	−178.99 (11)	C5—Si1—N1—B1	−3.01 (14)
C24—C11—B1—N1	−100.06 (15)	C4—N1—B1—C11	−168.01 (11)
C24—C11—B1—C1	81.36 (15)	C4—N1—B1—C1	10.55 (17)
C24—C19—C18—C17	0.99 (19)	C6—Si1—N1—C4	59.67 (10)
C24—C19—C20—C21	0.1 (2)	C6—Si1—N1—B1	−120.89 (12)
C24—C23—C22—C21	−0.6 (2)	B1—N1—C4—C3	−36.40 (16)
C17—C12—C13—C14	−2.04 (19)	B1—C11—C12—C17	177.96 (11)
C17—C16—C15—C14	−1.4 (2)	B1—C11—C12—C13	−2.47 (18)
C18—C19—C24—C11	1.37 (19)	B1—C11—C24—C19	−179.00 (11)
C18—C19—C24—C23	179.94 (12)	B1—C11—C24—C23	2.49 (18)
C18—C19—C20—C21	179.65 (13)		

### Hydrogen-bond geometry (Å, º)

*Cg*2, *Cg*5, *Cg*7 and *Cg*3 are the centroids of rings C11–C12/C17–C19/C24, C11–C18, C11–C24 and C12–C17, respectively.

**Table:**

*D*—H···*A*	*D*—H	H···*A*	*D*···*A*	*D*—H···*A*
C5—H5*A*···*Cg*2	0.98	2.86	3.4529 (15)	120
C5—H5*A*···*Cg*5	0.98	2.91	3.7305 (15)	142
C5—H5*A*···*Cg*7	0.98	2.88	3.4772 (15)	120
C2—H2···*Cg*3^i^	0.95	2.92	3.6380 (15)	133
C4—H4*B*···*Cg*3^ii^	0.99	2.97	3.9373 (15)	167

Symmetry codes: (i) −*x*+1, −*y*+1, −*z*+1; (ii) −*x*+3/2, *y*−1/2, *z*.
