# Supplementary material for: Synthesis and crystal structure of 2-(anthracen-9-yl)-1-(tert-butyl­dimethyl­sil­yl)-3,6-di­hydro-1λ4,2λ4-aza­borinine
Source: Acta Crystallogr E Crystallogr Commun. 2023 Oct 10;79(Pt 11):1012–6. doi: 10.1107/S2056989023008381 (PMC10626957; doi:10.1107/S2056989023008381)
Supplement: Supplementary file 3 [file e-79-01012-sup4.pdf]

Supporting Information for

Synthesis and crystal structure of 2-(anthracen-9-yl)-1-(*tert*-butyldimethylsilyl)-3,6-dihydro-1λ^4,2λ^4-azaborinine

Philipp J. Gliese,[a,b] Yannik Appiarius,[a,b], Tarek Scheele[c], Enno Lork[d], Tim Neudecker[b,c,e] and Anne Staubitz[a,b*]

[a]University of Bremen, Institute for Organic and Analytic Chemistry, 28359 Bremen, Germany, [b]University of Bremen, MAPEX Center for Materials and Processes, 28359 Bremen, Germany, [c]University of Bremen, Institute for Physical and Theoretical Chemistry, 28359 Bremen, Germany, [d]University of Bremen, Institute for Inorganic Chemistry and Crystallography, 28359 Bremen, Germany, [e]Bremen Center for Computational Materials Science, 28359 Bremen, Germany.

Correspondence e-mail: staubitz@uni-bremen.de

Table of Contents

Abbreviations 2
General Methods and Materials 3
NMR Spectroscopy 3
Mass Spectrometry 3
IR Spectroscopy 3
Melting Points 3
Computational Analysis 3
NMR Spectra 4
Platon Output 6
Computationally Optimized Geometry 18
References 19

Abbreviations

ATR	Attenuated total reflection
COSY	Correlation spectroscopy
DFT	Density Functional Theory
ESI	Electrospray ionization
HMBC	Heteronuclear multiple-bond correlation spectroscopy
HR	High resolution
HSQC	Heteronuclear single-quantum correlation spectroscopy
FT-IR	Fourier-transform infrared spectroscopy
m (NMR)	Multiplet (NMR)
NBO	Natural Bond Orbital
NLMO	Natural Localized Molecular Orbital
NMR	Nuclear magnetic resonance
NPA	Natural Population Analysis
s (NMR)	Singlet (NMR)
t (NMR)	Triplet
TMS	Tetramethylsilane

General Methods and Materials

Unless stated otherwise, all syntheses were carried out under standard Schlenk conditions under an atmosphere of nitrogen or argon. If necessary, reactions were carried out in a nitrogen-flushed glovebox from Inert Innovative Technology Inc. or reagents were prepared and stored there. All used glassware was heated under a vacuum below 0.1 mbar and flushed with inert gas at least three times before use. Syringes were flushed with inert gas at least three times before use. NMR tubes were dried in an oven at 110 °C for at least 2 h before use.

NMR Spectroscopy

^{1}H (601 MHz), $^{13}C\{^{1}H\}$ (151 MHz), $^{11}B\{^{1}H\}$ (193 MHz) and $^{29}Si\{^{1}H\}$ (119 MHz) NMR spectra were recorded at 298 K on a Bruker AVANCE NEO 600 spectrometer (Bruker BioSpin, Rheinstetten, Germany). The chemical shifts (δ) are given in parts per million (ppm). NMR spectra were referenced against tetramethylsilane, the residual solvent signals (^{1}H, $CDCl_3$: 7.26 ppm) or the solvent itself ($^{13}C\{^{1}H\}$, $CDCl_3$: 77.16 ppm). The assignment of signals was performed using two-dimensional NMR experiments such as $^{1}H/^{1}H$ COSY, $^{1}H/^{13}C\{^{1}H\}$ HSQC and $^{1}H/^{13}C$ HMBC. The positions of hydrogen and carbon atoms are labelled with numbers to simplify their assignment.

Mass Spectrometry

High-resolution ESI mass spectra were recorded on a Bruker Impact II (Bruker Daltonics, Bremen, Germany).

IR Spectroscopy

IR spectra were recorded on a Thermo Scientific Nicolet FT-IR Spectrometer System spectrometer with an ATR unit, equipped with a diamond window. The resolution was 4 cm^{-1}. The absorption bands are reported in cm^{-1}.

Melting Points

Melting points were measured on a Büchi M-560 Melting Point (Büchi, Flawil, Switzerland) device.

Computational Analysis

A geometry optimization based on density functional theory (DFT) was performed using Q-Chem 5.4.0 (Epifanovsky et al., 2021). The structure was optimized at the B3LYP/6-31G* level of theory (Becke, 1993; Francl et al., 1982; Hariharan et al., 1973; Hehre et al., 1972; Stephens et al., 1994). A natural bond orbital (NBO) analysis of the optimized structure was performed using the NBO 5.0 program package (Glendening et al., 2001). To determine bond orders, a natural population analysis (NPA) of the calculated natural localized molecular orbitals (NLMOs) was performed.

NMR Spectra

^{1}H NMR [600 MHz, $CDCl_3$, δ (ppm)]:

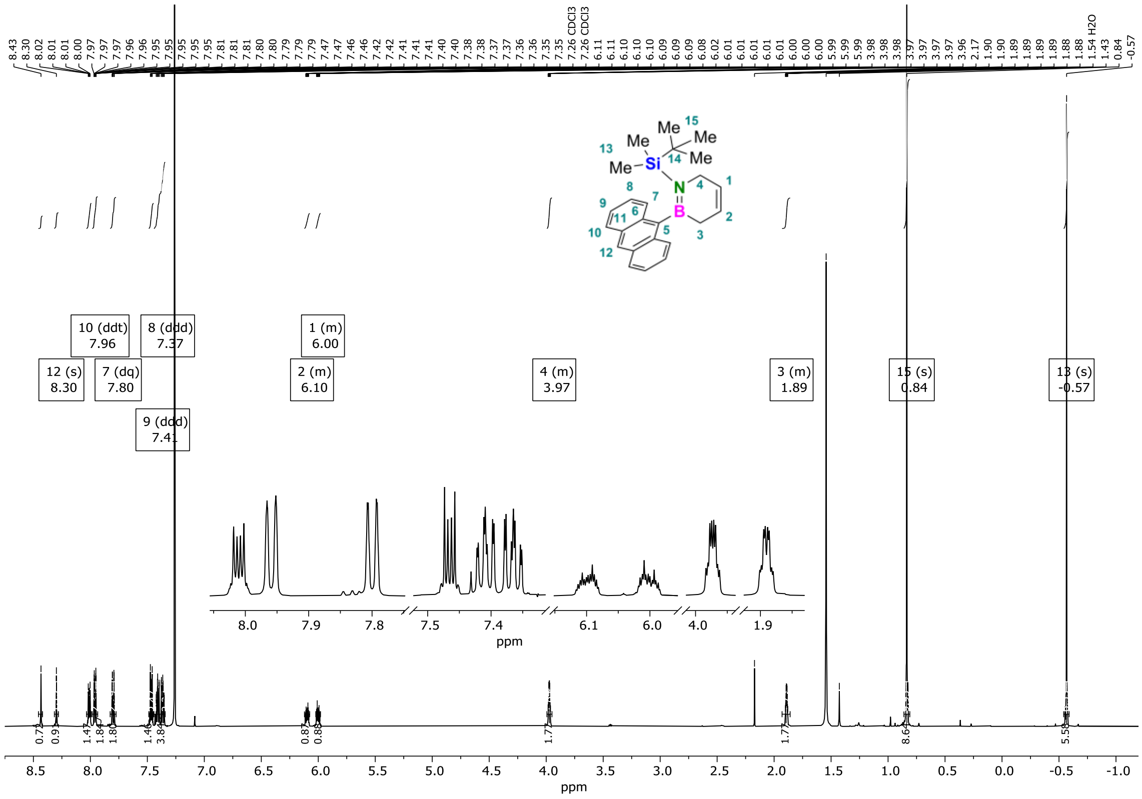

$^{13}C\{^1H\}$ NMR [151 MHz, $CDCl_3$, δ (ppm)]:

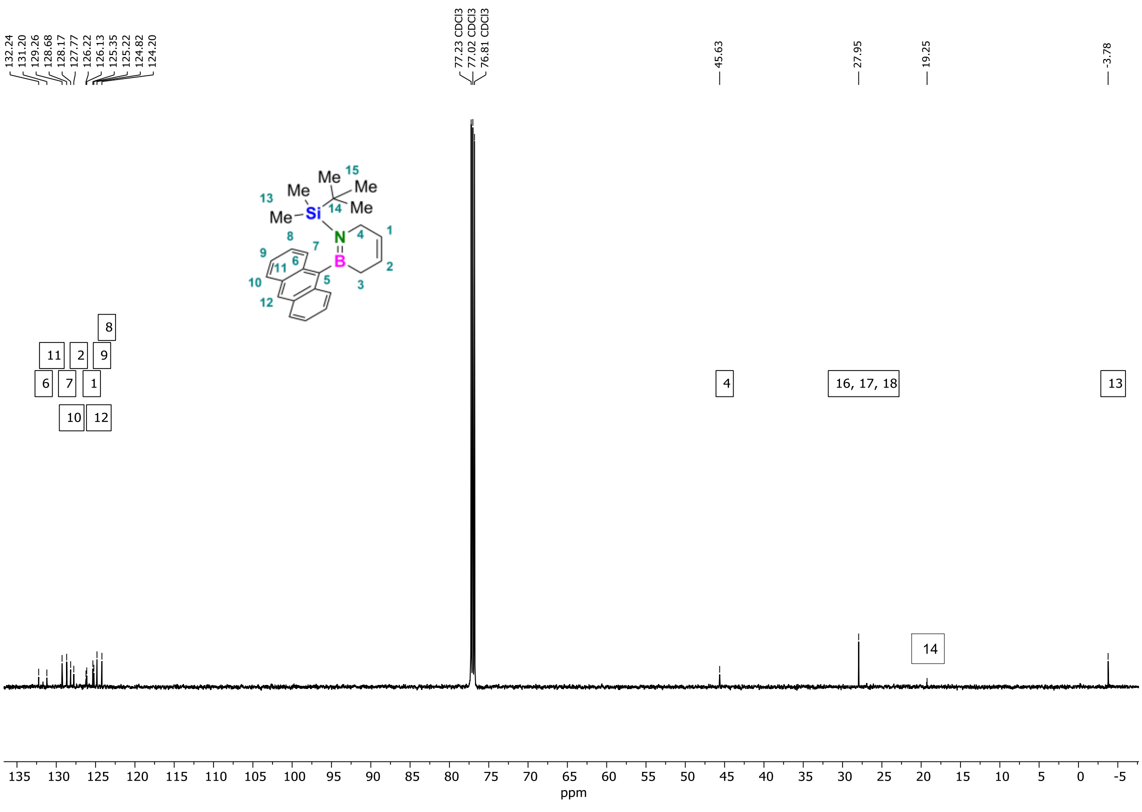

$^{11}B\{^{1}H\}$ NMR [160 MHz, $CDCl_3$, δ (ppm)]:

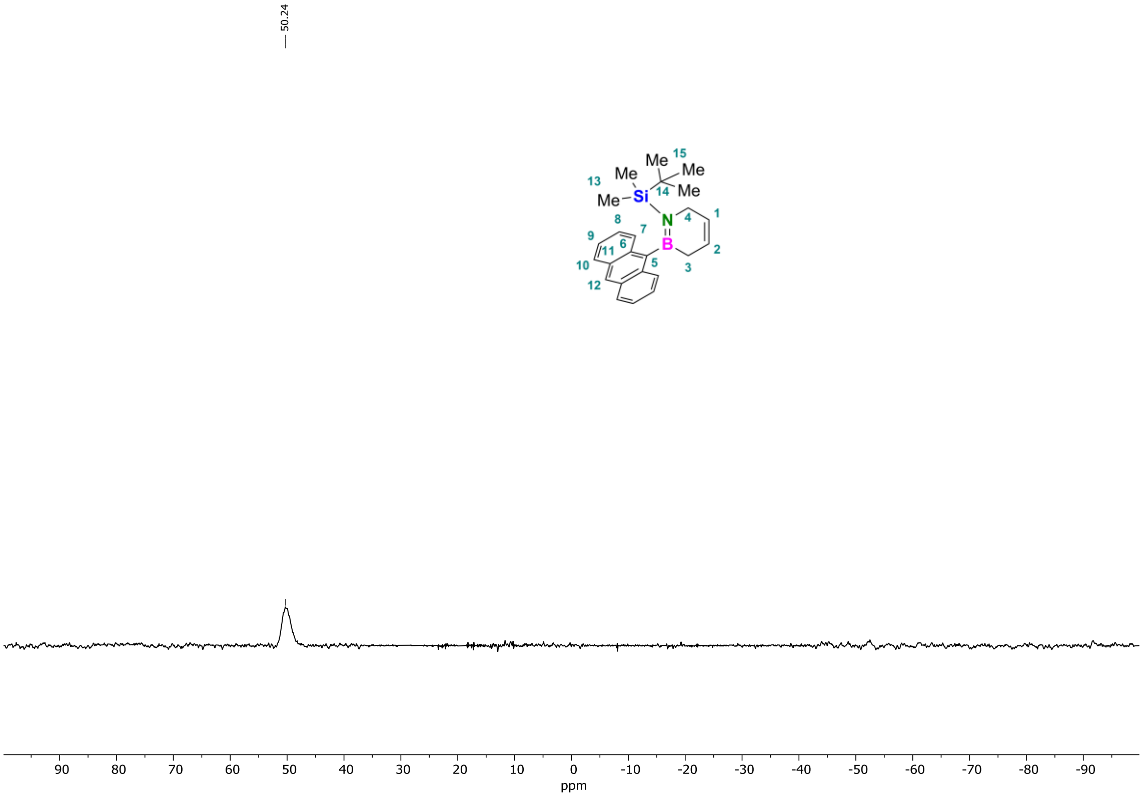

$^{29}Si\{^{1}H\}$ NMR [119 MHz, $CDCl_3$, δ (ppm)]:

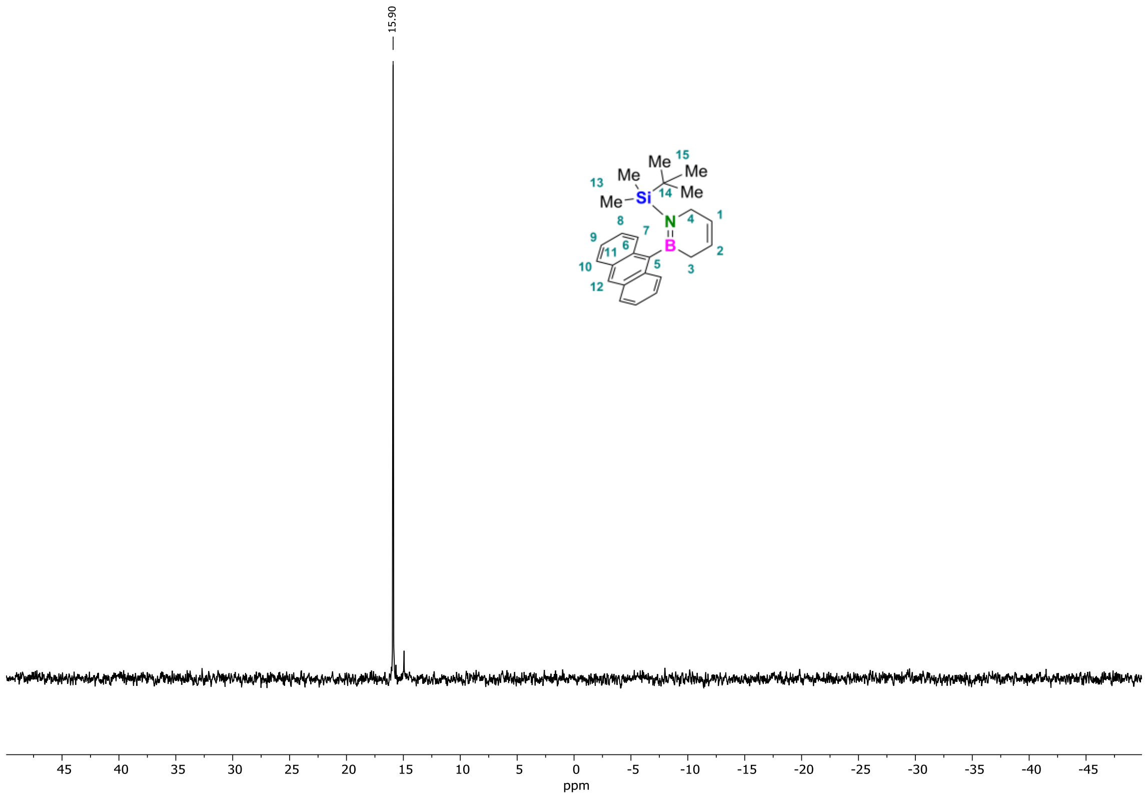

Platon Output

```
                                                                                      "gliesep200"  PLATON-GENERAL   Page    1
==============================================================================================================================
PLATON(V-301021)-Run for: gliesep200207_0maP b c a      R = 0.04                                    TIME: sday, 31 August 2023
==============================================================================================================================
                                                                                                        (C) 1980-2021 A.L.Spek
                                                      ============
====================================================== Crystal Data ==========================================================
                                                      ============
                       Input Cell  (Lattice Type: P)   -   Temp = 100K           Reduced Cell     (Acta Cryst.(1976),A32,297-298)
----------------------------------------------------------------------------   ---------------------------------------------
a =    13.3292(6)  Angstrom                alpha =           90 Degree          a  =   11.137     alpha  =   90.00  V  =  4245.0
b =    11.1365(5)                           beta =           90                 b  =   13.329     beta   =   90.00
c =   28.5973(12)                          gamma =           90                 c  =   28.597     gamma  =   90.00

V =     4245.0(3) Cubic-Angstrom          d(100) =     13.3292   Angstrom                   Niggli Values
                                          d(010) =     11.1365                          124.022   177.668   817.806
Lambda(MoKa) =   0.71073 Angstrom         d(001) =     28.5973                            0.000     0.000     0.000

                                                 ==========================
================================================ Orthogonalization Matrices ==================================================
                                                 ==========================

(See e.g. J.D.Dunitz, Xray Analysis and Structure Determination of Organic Molecules, Cornell Univ. Press, 1979, P236)

(XO)   (  13.32920         0         0 ) (X)   ,   (X)   (   0.07502         0         0 ) (XO)      Orthogonal Axes AO, BO and CO
(YO) = (         0  11.13650         0 )*(Y)   ,   (Y) = (         0   0.08979         0 )*(YO)             are defined as:
(ZO)   (         0         0  28.59730 ) (Z)   ,   (Z)   (         0         0   0.03497 ) (ZO)     AO // A, CO // C*, BO // CO X AO

                                                   ====================
================================================== Space Group Symmetry ======================================================
                                                   ====================

(See e.g. G. Burns & A.M. Glazer, Space Groups for Solid State Scientists, Academic Press, 1990 or Int. Tables A)

Space Group  H-M:  Pbca                                   Laue:  mmm
Space Group Hall: -P 2ac 2ab                       [Schoenflies: D2h^15]
Lattice Type: oP,  Centric, Orthorhombic, Multiplicity:   8( 4), No:  61

Non-Sohnke - No Absolute Structure

  Nr           ***** Symmetry Operation(s) *****

   1              X ,             Y ,             Z
   2        1/2 - X ,           - Y ,       1/2 + Z
   3        1/2 + X ,       1/2 - Y ,           - Z
   4            - X ,       1/2 + Y ,       1/2 - Z
   5            - X ,           - Y ,           - Z
   6        1/2 + X ,             Y ,       1/2 - Z
   7        1/2 - X ,       1/2 + Y ,             Z
   8              X ,       1/2 - Y ,       1/2 + Z
```

```
                                                                                      "gliesep200"  PLATON-GEOMETRY  Page    2
==============================================================================================================================
(1:19-Rule Rounded) Coordinates of Unique Residue(s) Identified.  Standard Deviations in the Last Digit are in Parentheses.
------------------------------------------------------------------------------------------------------------------------------
          Site    = Site Symmetry; SSN = Site Symmetry Number; SSOF = SHELX Site Occupation Factor (= S.O.F / SSN).
*******   Move    = Transformation on Input Data: N.IJK (N = SymOp, IJK = Translation) i.e. 1.555 = nomove
SYMBOLS:  Type    = D/A = Potential Donor or Acceptor atom, D-H = H on Donor atom, MET = Metal.
*******   El Type = AK = Alkali Metal, AE = Alkaline Earth, HL = Halogen, AN = Actinide, LN = Lanthanide, TR = Transition Element.
          ARU     = Asymmetric Residue Unit encoded as sklm.nn, with s = symmetry op, klm = translation, nn = residue #.
          RESIDUE = collection of ARU's constituting an isolated unit (= molecule, ion).
          FLAGS   : d = determined, c = calculated, R = riding G = group
------------------------------------------------------------------------------------------------------------------------------
```

Atom Types :	C	H	B	N	Si
Cov.Rad(Ang):	0.68	0.35	0.83	0.68	1.20
Atom Volume :	13.87	5.08	13.24	11.80	37.30
Atom Number :	6	1	5	7	14
Atom Weight :	12.010	1.008	10.81	14.01	28.09
Scat.Fact.f0:	5.999	1.000	4.999	6.995	13.998
Scat.Fact.f':	0.003	0.000	0.001	0.006	0.082
Scat.Fact.f":	0.002	0.000	0.001	0.003	0.070
Mu/Rho(MoKa):	0.58	0.37	0.37	0.84	6.65
Elem. Type :	--	--	--	--	--

```
------------------------------------------------------------------------------------------------------------------------------
Sources - Cov. Radii : Manual Cambridge Crystallographic Data Base
        - Atom Volume: D.W.M. Hofmann (2002). Acta Cryst. B58, 489-493
        - Atomic Wt. : SHELXL                                                                                           NOMOVE
        - Scat. Fact.: SHELXL (International Tables)
        - mu/rho     : International Tables C, Table 4.2.4.2, 193-199 - [Multiply by Atom Weight and 1.66054 for Barns/atom values]
------------------------------------------------------------------------------------------------------------------------------
```

Flags	Label	Fractional Coordinates (x,y,z)			Orthogonal Coordinates (XO,YO,ZO)			Site	SSN*SSOF =		S.O.F	Move	Type
d	Si1	0.64414(3)	0.53526(3)	0.67676(2)	8.5859(4)	5.9609(3)	19.3535(6)	1	1	1	1	-	-
d	N1	0.59622(8)	0.46229(9)	0.62665(4)	7.9471(11)	5.1483(10)	17.9205(11)	1	1	1	1	-	D/A
d	C1	0.48884(10)	0.41613(12)	0.55382(5)	6.5158(13)	4.6342(13)	15.8378(14)	1	1	1	1	-	-
d	C2	0.48012(10)	0.29143(12)	0.57271(5)	6.3996(13)	3.2455(13)	16.3780(14)	1	1	1	1	-	-
d	C3	0.54188(11)	0.25311(12)	0.60593(5)	7.2228(15)	2.8188(13)	17.3280(14)	1	1	1	1	-	-
d	C4	0.62300(10)	0.33186(11)	0.62535(5)	8.3041(13)	3.6958(12)	17.8833(14)	1	1	1	1	-	-
d	C5	0.61517(11)	0.69869(12)	0.67683(5)	8.1997(15)	7.7810(13)	19.3555(14)	1	1	1	1	-	-
d	C6	0.78352(10)	0.52013(13)	0.67616(6)	10.4437(13)	5.7924(14)	19.3363(17)	1	1	1	1	-	-
d	C7	0.58707(11)	0.46528(13)	0.73184(5)	7.8252(15)	5.1816(14)	20.9286(14)	1	1	1	1	-	-
d	C8	0.47669(12)	0.43301(16)	0.72304(6)	6.3539(16)	4.8222(18)	20.6770(17)	1	1	1	1	-	-
d	C9	0.64271(14)	0.35359(15)	0.74992(6)	8.5668(19)	3.9378(17)	21.4457(17)	1	1	1	1	-	-
d	C10	0.59141(18)	0.55923(17)	0.77124(6)	7.883(2)	6.2279(19)	22.0554(17)	1	1	1	1	-	-
d	C11	0.52126(9)	0.64751(11)	0.58088(4)	6.9480(12)	7.2110(12)	16.6116(11)	1	1	1	1	-	-
d	C12	0.59392(9)	0.71475(11)	0.55610(4)	7.9165(12)	7.9598(12)	15.9030(11)	1	1	1	1	-	-
d	C13	0.68530(10)	0.66195(12)	0.53987(4)	9.1345(13)	7.3718(13)	15.4388(11)	1	1	1	1	-	-
d	C14	0.75406(10)	0.72648(12)	0.51500(5)	10.0510(13)	8.0904(13)	14.7276(14)	1	1	1	1	-	-
d	C15	0.73675(11)	0.84979(13)	0.50489(5)	9.8203(15)	9.4637(14)	14.4385(14)	1	1	1	1	-	-
d	C16	0.65239(10)	0.90463(12)	0.52065(5)	8.6958(13)	10.0744(13)	14.8892(14)	1	1	1	1	-	-
d	C17	0.57825(10)	0.84029(12)	0.54660(5)	7.7076(13)	9.3579(13)	15.6313(14)	1	1	1	1	-	-

```
                                                                                      "gliesep200"  PLATON-GEOMETRY  Page    3
==============================================================================================================================
```

d	C18	0.49112(10)	0.89607(12)	0.56257(5)	6.5462(13)	9.9791(13)	16.0880(14)	1	1	1	1	-	-
d	C19	0.41726(10)	0.83144(12)	0.58633(5)	5.5617(13)	9.2593(13)	16.7675(14)	1	1	1	1	-	-
d	C20	0.32658(11)	0.88722(13)	0.60194(5)	4.3530(15)	9.8805(14)	17.2139(14)	1	1	1	1	-	-
d	C21	0.25508(11)	0.82314(14)	0.62480(5)	3.4000(15)	9.1669(16)	17.8676(14)	1	1	1	1	-	-
d	C22	0.26936(10)	0.69877(14)	0.63369(5)	3.5904(13)	7.7819(16)	18.1218(14)	1	1	1	1	-	-
d	C23	0.35484(10)	0.64256(12)	0.61956(5)	4.7297(13)	7.1559(13)	17.7177(14)	1	1	1	1	-	-

```
d     C24        0.43227(9)    0.70565(11)     0.59506(4)  5.7618(12)  7.8585(12) 17.0171(11)     1  1     1          1    -     -
d     B1        0.53820(10)    0.50755(13)     0.58960(5)  7.1738(13)  5.6523(14) 16.8610(14)     1  1     1          1    -     -

cRU   H1A           0.42113        0.44547        0.54535      5.6133      4.9610      15.5954     1  1     1          1    -     -
cRU   H1B           0.52972        0.41418        0.52492      7.0607      4.6125      15.0114     1  1     1          1    -     -
cRU   H2            0.42991        0.23911        0.56084      5.7304      2.6628      16.0384     1  1     1          1    -     -
cRU   H3            0.53448        0.17382        0.61769      7.1243      1.9357      17.6641     1  1     1          1    -     -
cRU   H4A           0.63894        0.30496        0.65749      8.5166      3.3962      18.8026     1  1     1          1    -     -
cRU   H4B           0.68421        0.32166        0.60615      9.1199      3.5821      17.3341     1  1     1          1    -     -
cRU   H5A           0.63998        0.73506        0.64785      8.5304      8.1860      18.5267     1  1     1          1    -     -
cRU   H5B           0.64786        0.73690        0.70368      8.6354      8.2065      20.1235     1  1     1          1    -     -
cRU   H5C           0.54243        0.71021        0.67901      7.2302      7.9092      19.4177     1  1     1          1    -     -
cRU   H6A           0.80167        0.43486        0.67595     10.6856      4.8428      19.3302     1  1     1          1    -     -
cRU   H6B           0.81159        0.55850        0.70409     10.8178      6.2197      20.1350     1  1     1          1    -     -
cRU   H6C           0.81053        0.55910        0.64813     10.8036      6.2264      18.5348     1  1     1          1    -     -
cRU   H8A           0.44050        0.50444        0.71227      5.8714      5.6177      20.3691     1  1     1          1    -     -
cRU   H8B           0.44642        0.40382        0.75213      5.9504      4.4971      21.5089     1  1     1          1    -     -
cRU   H8C           0.47271        0.37019        0.69912      6.3008      4.1226      19.9930     1  1     1          1    -     -
cRU   H9A           0.63560        0.28808        0.72728      8.4721      3.2082      20.7981     1  1     1          1    -     -
cRU   H9B           0.61402        0.32894        0.77997      8.1844      3.6632      22.3052     1  1     1          1    -     -
cRU   H9C           0.71396        0.37255        0.75403      9.5165      4.1489      21.5631     1  1     1          1    -     -
cRU   H10A          0.66097        0.58525        0.77576      8.8102      6.5177      22.1848     1  1     1          1    -     -
cRU   H10B          0.56608        0.52371        0.80028      7.5454      5.8323      22.8860     1  1     1          1    -     -
cRU   H10C          0.54994        0.62855        0.76276      7.3302      6.9999      21.8129     1  1     1          1    -     -
cRU   H13           0.69848        0.57990        0.54657      9.3101      6.4581      15.6305     1  1     1          1    -     -
cRU   H14           0.81381        0.68882        0.50437     10.8474      7.6710      14.4237     1  1     1          1    -     -
cRU   H15           0.78434        0.89383        0.48708     10.4547      9.9542      13.9292     1  1     1          1    -     -
cRU   H16           0.64241        0.98750        0.51429      8.5629     10.9973      14.7074     1  1     1          1    -     -
cRU   H18           0.48185        0.97951        0.55720      6.4227     10.9083      15.9344     1  1     1          1    -     -
cRU   H20           0.31621        0.97033        0.59624      4.2148     10.8061      17.0507     1  1     1          1    -     -
cRU   H21           0.19527        0.86164        0.63489      2.6028      9.5956      18.1562     1  1     1          1    -     -
cRU   H22           0.21889        0.65452        0.64961      2.9177      7.2891      18.5772     1  1     1          1    -     -
cRU   H23           0.36331        0.55955        0.62612      4.8426      6.2314      17.9052     1  1     1          1    -     -
```

```
                                                                                        "gliesep200"  PLATON-GEOMETRY  Page    4
===================================================================================================================================

   Ordered Structure                                  Unit Cell Contents (Based on Contents of Atom List, that may be Incomplete)
===================================================================================================================================
Resd Site  X(cen) Y(cen) Z(cen)  Mol.Wt S.O.F   Z       C     H    B    N   Si
===================================================================================================================================
  1     1  0.550  0.608  0.619   371.39     1   8      24    30    1    1    1
                           ---------------------------------------------------------------------------------------------------------
        Unit Cell Weight =      2971.12             192   240    8    8    8

 Calculated Analysis (%) =                         77.6   8.1  2.9  3.8  7.6

          Moiety_Formula = C24 H30 B N Si

             Sum_Formula = C24 H30 B N Si

          Formula_Weight =      371.39 [Note: Based on SHELXL2014 Atomic Weights]

               Formula_Z =           8

            SpaceGroup_Z =           8  ===>  Z' =  8 /  8 =   1.000

      Calculated Density =   1.1622(1) g cm-3 [= Mg m-3]                         ** WARNING **
                                                                       Please Check the Derived Crystal Data.
                 F(000) =      1600.0 [    1601.11]                    They may be Incorrect for Disordered,
                                                                       Incomplete or Polymeric Structures.
               mu(MoKa) =        1.19 cm-1 =   0.119 mm-1


        Predicted Volume =      4381.0[    4298.6] Ang**3, 298[100]K - (D.W.M. Hofmann (2002). Acta Cryst. B58, 489-493)

Note on F000: The first number is a pure electron count, the second number between [] is calculated from f,f' & f"
```

```
                                                                                        "gliesep200"  PLATON-MOLSYM    Page    5
===================================================================================================================================
MOLSYM: Search for (additional) Molecular (Point Group) Symmetry - Hydrogen Atoms Excluded ! - Ordered Residues Only !
===================================================================================================================================

For Details on the Molecular Symmetry Determination see: T. Pilati & A. Forni, J.Appl.Cryst. (1998), 31, 503-504 & (2000), 33, 417.

For CSM (i.e. Continuous Symmetry Measure), see: H. Zabrodsky et al. (1993) JACS, 115, 8278-8289

 ***** Weighting Mode = ATOMIC *****

Resd 1          No Molecular Symmetry Within Tolerance =  0.80 Ang.
Resd 1          No Molecular Symmetry Within Tolerance =  0.80 Ang.
```

```
                                                                                        "gliesep200"  PLATON-MOLSYM    Page    6
===================================================================================================================================
MOLSYM: Search for (additional) Molecular (Point Group) Symmetry - Hydrogen Atoms Excluded ! - Ordered Residues Only !
===================================================================================================================================

For Details on the Molecular Symmetry Determination see: T. Pilati & A. Forni, J.Appl.Cryst. (1998), 31, 503-504 & (2000), 33, 417.

For CSM (i.e. Continuous Symmetry Measure), see: H. Zabrodsky et al. (1993) JACS, 115, 8278-8289

 ***** Weighting Mode = UNIT   *****

Resd 1          No Molecular Symmetry Within Tolerance =  0.80 Ang.
Resd 1          No Molecular Symmetry Within Tolerance =  0.80 Ang.
```

```
                                                                                        "gliesep200"  PLATON-NONSYM    Page    7
===================================================================================================================================
NONSYM Search for Additional (Non)Crystallographic Symmetry between Residues (Experimental)
===================================================================================================================================

- Residue numbers with opposite signs indicate potential enantiomeric pairs
- Hydrogen atoms omitted from the analysis
- Only residues with more than 6 atoms are analysed


RES# Coords Center of Gravity        Main axes (hor)   EigenV   Asym Angle a,b,c
--------------------------------------------------------------------------------
  1  0.5487   0.6126   0.6168    0.425  0.642  0.638    3324   35.96  65  50  50
     7.3134   6.8220  17.6376   -0.892  0.177  0.417    2856   45.41 153  80  65
       Res.Mol.Wt. =   341.15    0.154 -0.746  0.648    1832   47.49  81 138  50
```

```
                                                                                        "gliesep200"  PLATON-ADP-Anal  Page    8
===================================================================================================================================
(An)isotropic, Equivalent and Main Axes Displacement Parameters - Unusual Values Marked with a # - [Optional Coordinate Split-up]
-----------------------------------------------------------------------------------------------------------------------------------
-----------------------------------------------------------------------------------------------------------------------------------
```

Atom	Label	U11 or Uiso	U22	U33	U23	U13	U12	Ueq(sUeq)	U1	U2	U3	U3/U1
1	Si1	0.01541(18)	0.01078(17)	0.01295(17)	-0.00092(13)	-0.00323(13)	-0.00045(13)	0.01305(10)	0.0097	0.0118	0.0176	1.81
		[0.6466	0.5354	0.6764] &	[0.6417	0.5352	0.6771]					
2	N1	0.0150(5)	0.0087(5)	0.0127(5)	-0.0003(4)	-0.0005(4)	0.0004(4)	0.0121(3)	0.0087	0.0126	0.0151	1.75
		[0.5985	0.4625	0.6265] &	[0.5940	0.4621	0.6268]					
3	C1	0.0204(7)	0.0136(6)	0.0152(6)	0.0009(5)	-0.0034(5)	-0.0005(5)	0.0164(4)	0.0129	0.0141	0.0222	1.71
		[0.4914	0.4157	0.5535] &	[0.4863	0.4165	0.5541]					
4	C2	0.0200(7)	0.0127(6)	0.0224(7)	-0.0027(5)	-0.0023(5)	-0.0042(5)	0.0184(4)	0.0099	0.0215	0.0238	2.42
		[0.4769	0.2912	0.5739] &	[0.4833	0.2916	0.5715]					
5	C3	0.0249(7)	0.0088(6)	0.0221(7)	0.0007(5)	-0.0014(5)	-0.0019(5)	0.0186(4)	0.0086	0.0215	0.0257	3.01
		[0.5468	0.2522	0.6055] &	[0.5369	0.2540	0.6064]					
6	C4	0.0204(7)	0.0097(6)	0.0164(6)	-0.0009(5)	-0.0047(5)	0.0031(5)	0.0155(4)	0.0088	0.0135	0.0242	2.73
		[0.6272	0.3334	0.6248] &	[0.6188	0.3303	0.6259]					
7	C5	0.0262(7)	0.0131(6)	0.0190(7)	-0.0036(5)	-0.0063(6)	-0.0001(5)	0.0194(4)	0.0108	0.0175	0.0300	2.79
		[0.6201	0.6996	0.6762] &	[0.6102	0.6978	0.6775]					
8	C6	0.0174(7)	0.0185(7)	0.0349(8)	0.0005(6)	-0.0076(6)	-0.0013(5)	0.0236(4)	0.0143	0.0187	0.0378	2.64
		[0.7800	0.5208	0.6782] &	[0.7870	0.5194	0.6741]					
9	C7	0.0302(8)	0.0196(7)	0.0125(6)	0.0005(5)	0.0000(5)	0.0011(6)	0.0208(4)	0.0125	0.0195	0.0303	2.43
		[0.5917	0.4660	0.7318] &	[0.5824	0.4646	0.7318]					
10	C8	0.0264(8)	0.0412(10)	0.0256(8)	0.0085(7)	0.0092(6)	-0.0016(7)	0.0311(5)	0.0147	0.0332	0.0453	3.07
		[0.4776	0.4410	0.7237] &	[0.4758	0.4250	0.7224]					
11	C9	0.0447(10)	0.0350(9)	0.0238(8)	0.0144(7)	0.0028(7)	0.0081(8)	0.0345(5)	0.0138	0.0366	0.0531	3.86
		[0.6485	0.3608	0.7506] &	[0.6369	0.3464	0.7493]					
12	C10	0.0817(15)	0.0363(10)	0.0165(8)	-0.0064(7)	0.0059(9)	-0.0084(10)	0.0448(7)	0.0144	0.0361	0.0839	5.81
		[0.6038	0.5558	0.7715] &	[0.5790	0.5626	0.7710]					
13	C11	0.0138(6)	0.0115(6)	0.0105(6)	-0.0001(5)	-0.0033(4)	-0.0009(5)	0.0119(3)	0.0084	0.0115	0.0160	1.91
		[0.5237	0.6468	0.5806] &	[0.5188	0.6482	0.5812]					
14	C12	0.0149(6)	0.0115(6)	0.0114(6)	0.0004(5)	-0.0037(5)	-0.0008(5)	0.0126(3)	0.0091	0.0114	0.0174	1.92
		[0.5965	0.7141	0.5557] &	[0.5913	0.7154	0.5565]					
15	C13	0.0177(6)	0.0132(6)	0.0150(6)	-0.0012(5)	-0.0005(5)	-0.0005(5)	0.0153(3)	0.0125	0.0156	0.0178	1.42
		[0.6869	0.6618	0.5398] &	[0.6837	0.6621	0.5399]					
16	C14	0.0180(6)	0.0195(7)	0.0171(6)	-0.0027(5)	0.0020(5)	-0.0019(5)	0.0182(4)	0.0151	0.0167	0.0228	1.51
		[0.7530	0.7286	0.5148] &	[0.7551	0.7244	0.5152]					
17	C15	0.0210(7)	0.0210(7)	0.0164(6)	0.0030(5)	0.0001(5)	-0.0092(6)	0.0195(4)	0.0109	0.0170	0.0305	2.80
		[0.7401	0.8449	0.5047] &	[0.7334	0.8547	0.5050]					
18	C16	0.0212(7)	0.0133(6)	0.0193(7)	0.0046(5)	-0.0053(5)	-0.0048(5)	0.0179(4)	0.0104	0.0149	0.0285	2.75
		[0.6563	0.9014	0.5199] &	[0.6485	0.9079	0.5214]					
19	C17	0.0173(6)	0.0122(6)	0.0144(6)	0.0009(5)	-0.0054(5)	-0.0028(5)	0.0146(3)	0.0097	0.0120	0.0222	2.28
		[0.5816	0.8386	0.5461] &	[0.5749	0.8419	0.5471]					
20	C18	0.0204(7)	0.0112(6)	0.0187(7)	0.0009(5)	-0.0055(5)	0.0011(5)	0.0168(4)	0.0106	0.0146	0.0251	2.37
		[0.4950	0.8962	0.5619] &	[0.4873	0.8959	0.5633]					
21	C19	0.0156(6)	0.0136(6)	0.0154(6)	-0.0010(5)	-0.0050(5)	0.0021(5)	0.0149(3)	0.0103	0.0132	0.0211	2.05

Atom	Label	U11 or Uiso	U22	U33	U23	U13	U12	Ueq(sUeq)	U1	U2	U3	U3/U1
		[0.4200	0.8330	0.5858] &	[0.4145	0.8298	0.5869]					
22	C20	0.0209(7)	0.0173(7)	0.0219(7)	-0.0020(5)	-0.0035(5)	0.0061(5)	0.0200(4)	0.0127	0.0193	0.0281	2.21
		[0.3296	0.8903	0.6014] &	[0.3236	0.8841	0.6025]					
23	C21	0.0168(7)	0.0256(7)	0.0238(7)	-0.0042(6)	0.0002(6)	0.0059(6)	0.0221(4)	0.0134	0.0222	0.0306	2.29
		[0.2565	0.8280	0.6244] &	[0.2536	0.8182	0.6252]					
24	C22	0.0160(7)	0.0259(7)	0.0202(7)	-0.0003(6)	0.0014(5)	-0.0021(6)	0.0207(4)	0.0152	0.0205	0.0264	1.73
		[0.2688	0.7022	0.6336] &	[0.2699	0.6953	0.6337]					
25	C23	0.0177(6)	0.0159(6)	0.0162(6)	0.0008(5)	-0.0013(5)	-0.0014(5)	0.0166(3)	0.0151	0.0154	0.0192	1.27
		[0.3558	0.6418	0.6194] &	[0.3539	0.6433	0.6197]					
26	C24	0.0136(6)	0.0130(6)	0.0126(6)	-0.0005(5)	-0.0036(5)	0.0001(5)	0.0131(3)	0.0094	0.0130	0.0168	1.78
		[0.4345	0.7061	0.5946] &	[0.4300	0.7052	0.5955]					
27	B1	0.0116(6)	0.0116(6)	0.0124(6)	0.0005(5)	0.0016(5)	-0.0003(5)	0.0119(3)	0.0101	0.0118	0.0137	1.35
		[0.5392	0.5078	0.5899] &	[0.5372	0.5073	0.5893]					
28	H1A	0.02000						0.02000				
29	H1B	0.02000						0.02000				
30	H2	0.02200						0.02200				
31	H3	0.02200						0.02200				
32	H4A	0.01900						0.01900				
33	H4B	0.01900						0.01900				
34	H5A	0.02900						0.02900				
35	H5B	0.02900						0.02900				
36	H5C	0.02900						0.02900				
37	H6A	0.03500						0.03500				
38	H6B	0.03500						0.03500				
39	H6C	0.03500						0.03500				
40	H8A	0.04700						0.04700				
41	H8B	0.04700						0.04700				
42	H8C	0.04700						0.04700				
43	H9A	0.05200						0.05200				
44	H9B	0.05200						0.05200				
45	H9C	0.05200						0.05200				
46	H10A	0.06700						0.06700				
47	H10B	0.06700						0.06700				
48	H10C	0.06700						0.06700				
49	H13	0.01800						0.01800				
50	H14	0.02200						0.02200				
51	H15	0.02300						0.02300				
52	H16	0.02200						0.02200				
53	H18	0.02000						0.02000				
54	H20	0.02400						0.02400				
55	H21	0.02700						0.02700				
56	H22	0.02500						0.02500				
57	H23	0.02000						0.02000				
U(i,j)-Average		0.0222	0.0172	0.0178	0.0004	-0.0016	-0.0006	0.0190	0.0170	0.0174	0.0228	1.34

The Displacement Factor has the Form of Exp(-T)

where

T = 8*(pi**2)*Uiso*sin(theta/lambda)**2, for Isotropic Atoms,

T = 2*(pi**2)*(U11*(h*as)**2+U22*(k*bs)**2+U33*(l*cs)**2+2*U23*k*l*bs*cs+2*U13*h*l*as*cs+2*U12*h*k*as*bs),for Anisotr. Atoms

Ueq = 1/3 Sum(i,j) (Uij*as(i)*as(j)*a(i).a(j))

U1, U2, U3 are the three Main Axes Components of Uij

Reference U(eq): R.X. Fischer & E. Tillmanns, Acta Cryst. (1988). C44, 775-776

Ueq [or U(iso)] Averages per Element

	Non-H	C	H	B	N	Si
Average	0.0191	0.0199	0.0338	0.0119	0.0121	0.0131
Minimum	0.0119	0.0119	0.0180	0.0119	0.0121	0.0131

Maximum 0.0448 0.0448 0.0670 0.0119 0.0121 0.0131
Ratio 3.7647 3.7647 3.7222 1.0000 1.0000 1.0000
Number 27 24 30 1 1 1

"gliesep200" PLATON-ADP-Anal Page 11

V.Schomaker and K.N.Trueblood Rigid Body Motion Analysis, TLS - Model (Acta Cryst. (1968), B24, 63-76) - see also Dunitz, p244

Observed Vibration Tensor in Inertial System I(1) = L, I(2) = M, I(3) = N (Difference U(calc) - U(obs) in Parentheses)

Label	U(L,L)<br>U11	U(L,M)<br>U12	U(L,N)<br>U13	U(M,M)<br>U22	U(M,N)<br>U23	U(N,N)<br>U33	Ueq(obs)	Ueq(cal)
Si1	0.01215[0.00171]	-0.00147[0.00116]	-0.00015[0.00418]	0.01724[0.00733]	-0.00004[-.00151]	0.00975[0.00507]	0.0130	0.0178
	0.01541[0.00770]	-0.00045[0.00088]	-0.00323[-.00155]	0.01078[0.00724]	-0.00092[0.00170]	0.01295[-.00083]		
N1	0.01063[0.00168]	-0.00080[0.00131]	-0.00181[0.00155]	0.01460[0.00246]	-0.00144[0.00097]	0.01117[0.00153]	0.0121	0.0140
	0.01500[0.00169]	0.00040[-.00121]	-0.00050[-.00062]	0.00870[0.00370]	-0.00030[0.00071]	0.01270[0.00028]		
C1	0.01300[0.00395]	-0.00072[0.00099]	0.00011[-.00302]	0.02210[-.00301]	-0.00023[0.00034]	0.01410[-.00529]	0.0164	0.0149
	0.02040[-.00285]	-0.00050[-.00294]	-0.00340[0.00158]	0.01360[-.00263]	0.00090[-.00415]	0.01520[0.00113]		
C2	0.02006[-.00063]	-0.00041[-.00011]	-0.00471[-.00221]	0.02282[0.00044]	0.00177[-.00006]	0.01222[0.00177]	0.0184	0.0189
	0.02000[0.00096]	-0.00420[0.00011]	-0.00230[0.00105]	0.01270[-.00177]	-0.00270[0.00088]	0.02240[0.00239]		
C3	0.01424[0.00256]	-0.00064[0.00002]	-0.00640[-.00083]	0.02565[-.00236]	-0.00081[0.00223]	0.01591[0.00327]	0.0186	0.0198
	0.02490[-.00280]	-0.00190[-.00034]	-0.00140[0.00147]	0.00880[0.00241]	0.00070[0.00073]	0.02210[0.00386]		
C4	0.01198[0.00170]	-0.00507[0.00410]	-0.00109[-.00216]	0.02175[-.00123]	-0.00232[0.00278]	0.01277[0.00535]	0.0155	0.0174
	0.02040[-.00067]	0.00310[-.00323]	-0.00470[0.00390]	0.00970[0.00276]	-0.00090[0.00301]	0.01640[0.00373]		
C5	0.01813[-.00056]	-0.00366[0.00253]	-0.00118[0.00568]	0.02872[-.00258]	-0.00225[-.00168]	0.01145[-.00131]	0.0194	0.0179
	0.02620[-.00107]	-0.00010[0.00044]	-0.00630[0.00038]	0.01310[0.00479]	-0.00360[-.00026]	0.01900[-.00817]		
C6	0.02365[-.00648]	-0.00748[0.00191]	-0.00412[0.00780]	0.02660[0.00432]	0.00859[-.00435]	0.02055[-.00267]	0.0236	0.0220
	0.01740[0.00560]	-0.00130[0.00188]	-0.00760[-.00321]	0.01850[0.00242]	0.00050[0.00155]	0.03490[-.01284]		
C7	0.01614[-.00028]	0.00271[0.00114]	0.00251[0.00487]	0.02652[0.00750]	-0.00626[0.00057]	0.01964[0.00840]	0.0208	0.0260
	0.03020[0.00672]	0.00110[0.00114]	0.00000[-.00199]	0.01960[0.00863]	0.00050[0.00486]	0.01250[0.00027]		
C8	0.02830[-.01107]	0.00967[-.00199]	0.00414[-.00079]	0.02165[0.00594]	0.00189[-.00759]	0.04325[-.00909]	0.0311	0.0263
	0.02640[0.00813]	-0.00160[0.00283]	0.00920[-.00353]	0.04120[-.01273]	0.00850[-.00126]	0.02560[-.00962]		
C9	0.01531[-.00099]	0.00301[-.00050]	0.00559[0.00236]	0.03826[0.00557]	-0.00630[0.00344]	0.04993[-.01281]	0.0345	0.0318
	0.04470[-.00097]	0.00810[-.00511]	0.00280[-.00813]	0.03500[-.00275]	0.01440[-.00301]	0.02380[-.00452]		
C10	0.03838[-.01528]	0.01918[-.01464]	0.00077[0.01254]	0.06629[-.02057]	-0.02306[0.01087]	0.02983[0.00124]	0.0448	0.0333
	0.08170[-.03042]	-0.00840[0.01283]	0.00590[-.01001]	0.03630[0.00197]	-0.00640[0.00840]	0.01650[-.00617]		
C11	0.01078[0.00170]	-0.00050[0.00097]	0.00129[-.00093]	0.01588[-.00105]	0.00056[-.00118]	0.00914[-.00230]	0.0119	0.0114
	0.01380[0.00007]	-0.00090[-.00095]	-0.00330[0.00124]	0.01150[-.00093]	-0.00010[-.00216]	0.01050[-.00079]		
C12	0.01060[0.00184]	-0.00077[0.00177]	0.00105[-.00097]	0.01725[-.00070]	0.00068[-.00050]	0.00996[-.00261]	0.0126	0.0121
	0.01490[0.00001]	-0.00080[-.00200]	-0.00370[0.00120]	0.01150[-.00064]	0.00040[-.00191]	0.01140[-.00083]		
C13	0.01524[-.00030]	-0.00001[0.00229]	-0.00103[0.00009]	0.01744[-.00169]	-0.00124[0.00164]	0.01322[-.00153]	0.0153	0.0141
	0.01770[-.00225]	-0.00050[-.00250]	-0.00050[0.00068]	0.01320[0.00021]	-0.00120[0.00047]	0.01500[-.00148]		
C14	0.02190[-.00337]	0.00225[0.00220]	-0.00024[-.00248]	0.01661[0.00159]	-0.00093[0.00314]	0.01609[0.00372]	0.0182	0.0188
	0.01800[0.00041]	-0.00190[-.00247]	0.00200[0.00091]	0.01950[-.00138]	-0.00270[0.00474]	0.01710[0.00291]		
C15	0.01831[-.00073]	0.00825[-.00302]	0.00004[-.00381]	0.02347[0.00049]	0.00445[0.00020]	0.01661[0.00553]	0.0195	0.0212
	0.02100[0.00090]	-0.00920[0.00267]	0.00010[0.00105]	0.02100[-.00252]	0.00300[0.00190]	0.01640[0.00691]		
C16	0.01160[0.00275]	0.00098[0.00114]	-0.00179[-.00117]	0.02675[-.00069]	0.00481[-.00072]	0.01544[0.00029]	0.0179	0.0187
	0.02120[0.00059]	-0.00480[-.00082]	-0.00530[0.00192]	0.01330[0.00065]	0.00460[-.00138]	0.01930[0.00112]		
C17	0.01194[0.00089]	-0.00055[0.00055]	0.00022[-.00111]	0.02167[-.00036]	0.00250[-.00105]	0.01030[-.00114]	0.0146	0.0144
	0.01730[0.00062]	-0.00280[-.00046]	-0.00540[0.00102]	0.01220[-.00114]	0.00090[-.00128]	0.01440[-.00009]		
C18	0.01234[0.00217]	-0.00416[0.00097]	-0.00135[-.00003]	0.02369[-.00055]	0.00045[0.00011]	0.01427[-.00445]	0.0168	0.0158
	0.02040[-.00100]	0.00110[-.00211]	-0.00550[-.00032]	0.01120[-.00039]	0.00090[-.00268]	0.01870[-.00143]		

"gliesep200" PLATON-ADP-Anal Page 12

C19	0.01389[0.00285]	-0.00443[0.00082]	0.00139[-.00226]	0.01841[0.00075]	0.00026[-.00118]	0.01231[-.00107]	0.0149	0.0157
	0.01560[0.00189]	0.00210[-.00121]	-0.00500[0.00144]	0.01360[-.00104]	-0.00100[-.00218]	0.01540[0.00169]		
C20	0.01914[0.00441]	-0.00716[0.00017]	0.00038[-.00318]	0.02134[-.00063]	-0.00256[0.00065]	0.01962[-.00020]	0.0200	0.0212
	0.02090[-.00047]	0.00610[-.00160]	-0.00350[0.00164]	0.01730[-.00049]	-0.00200[-.00217]	0.02190[0.00454]		
C21	0.02738[0.00197]	-0.00553[-.00036]	0.00228[-.00611]	0.01566[0.00181]	-0.00175[-.00038]	0.02316[0.00504]	0.0221	0.0250
	0.01680[0.00338]	0.00590[-.00003]	0.00020[0.00314]	0.02560[-.00280]	-0.00420[0.00066]	0.02380[0.00823]		
C22	0.02433[0.00219]	0.00229[-.00380]	0.00200[-.00523]	0.01662[-.00185]	0.00262[-.00333]	0.02115[0.00627]	0.0207	0.0229
	0.01600[0.00188]	-0.00210[0.00490]	0.00140[0.00392]	0.02590[-.00303]	-0.00030[-.00096]	0.02020[0.00776]		
C23	0.01536[0.00378]	0.00063[-.00060]	-0.00001[-.00141]	0.01891[-.00445]	0.00091[-.00131]	0.01553[0.00189]	0.0166	0.0170
	0.01770[-.00209]	-0.00140[0.00154]	-0.00130[0.00328]	0.01590[0.00093]	0.00080[-.00208]	0.01620[0.00238]		
C24	0.01259[0.00248]	-0.00182[0.00093]	0.00146[-.00153]	0.01598[-.00053]	0.00084[-.00166]	0.01064[-.00071]	0.0131	0.0135
	0.01360[0.00123]	0.00010[-.00055]	-0.00360[0.00195]	0.01300[-.00052]	-0.00050[-.00207]	0.01260[0.00054]		
B1	0.01184[0.00103]	0.00091[0.00004]	-0.00096[0.00016]	0.01072[0.00404]	-0.00008[-.00044]	0.01304[-.00420]	0.0119	0.0122
	0.01160[0.00280]	-0.00030[-.00168]	0.00160[-.00240]	0.01160[-.00097]	0.00050[-.00207]	0.01240[-.00096]		

R1 = Sum(abs(U(obs)-U(calc)))/Sum(abs(U(obs))) = 0.25200

R2 = Sqrt(Sum((U(obs)-U(calc))**2)/Sum(U(obs)**2)) = 0.27102

S = Sqrt(Sum((U(obs)-U(calc))**2)/(6*N-NS*M)) = 0.00444

N = Number of Atoms in Rigid Group = 27

NS = Symmetry Factor = 1

M = Number of Rigid-Body Parameters = 20

Largest abs(U(obs)-U(calc)) = 0.02057

TLS-Mode

:: No TLS-Analysis for Residue Nr: 1, Because R > 0.25

"gliesep200" PLATON-ADP-Anal Page 13

Rigid-Body Model Libration Corrections for Bond Distances and "Hirshfeld Rigid-Bond" Test (Acta Cryst., 1976, A32, 239-244)

Bond		Bond Distance		Components of the Correction			MSDA from U(obs) Vibration Along the Interatomic Bond				Angle with Lib. Axes		
Atom(I)	Atom(J)	Obsd	Calcd	Del(L)	Del(M)	Del(N)	I to J	J to I	Difference	Sqrt(Diff)	L(1)	L(2)	L(3)
Si1	- N1	1.7669(12)	0	0	0	0	0.01008(17)	0.0118(5)	0.0017(5)	0.0412	0.00	0.00	0.00
Si1	- C5	1.8606(14)	0	0	0	0	0.01116(17)	0.0137(7)	0.0025(7)	0.0500	0.00	0.00	0.00
Si1	- C6	1.8655(14)	0	0	0	0	0.01551(17)	0.0178(7)	0.0023(8)	0.0480	0.00	0.00	0.00
Si1	- C7	1.9150(15)	0	0	0	0	0.01556(17)	0.0165(7)	0.0009(7)	0.0300	0.00	0.00	0.00
N1	- C4	1.4962(16)	0	0	0	0	0.0089(5)	0.0089(6)	0.0000(8)	0	0.00	0.00	0.00
N1	- B1	1.4053(18)	0	0	0	0	0.0125(5)	0.0132(6)	0.0007(8)	0.0265	0.00	0.00	0.00
C1	- C2	1.4946(19)	0	0	0	0	0.0134(6)	0.0154(7)	0.0020(9)	0.0447	0.00	0.00	0.00
C1	- B1	1.586(2)	0	0	0	0	0.0141(6)	0.0131(6)	0.0010(9)	0.0316	0.00	0.00	0.00
C2	- C3	1.328(2)	0	0	0	0	0.0213(7)	0.0210(7)	0.0003(9)	0.0173	0.00	0.00	0.00
C3	- C4	1.4989(19)	0	0	0	0	0.0170(7)	0.0159(6)	0.0011(9)	0.0332	0.00	0.00	0.00
C7	- C8	1.535(2)	0	0	0	0	0.0297(7)	0.0300(9)	0.0003(11)	0.0173	0.00	0.00	0.00
C7	- C9	1.538(2)	0	0	0	0	0.0201(7)	0.0227(9)	0.0026(11)	0.0510	0.00	0.00	0.00
C7	- C10	1.539(2)	0	0	0	0	0.0164(7)	0.0193(11)	0.0029(13)	0.0539	0.00	0.00	0.00
C11	- C12	1.4145(17)	0	0	0	0	0.0140(6)	0.0148(6)	0.0008(8)	0.0283	0.00	0.00	0.00
C11	- C24	1.4109(17)	0	0	0	0	0.0153(6)	0.0149(6)	0.0004(8)	0.0200	0.00	0.00	0.00
C11	- B1	1.5946(19)	0	0	0	0	0.0117(6)	0.0116(6)	0.0001(8)	0.0100	0.00	0.00	0.00
C12	- C13	1.4300(18)	0	0	0	0	0.0167(6)	0.0170(6)	0.0003(8)	0.0173	0.00	0.00	0.00

```
C12    - C17     1.4395(18)          0        0        0        0  0.0114(6)   0.0126(6)   0.0012(8)     0.0346     0.00  0.00  0.00
C13    - C14     1.3647(19)          0        0        0        0  0.0164(6)   0.0169(6)   0.0005(9)     0.0224     0.00  0.00  0.00
C14    - C15       1.422(2)          0        0        0        0  0.0212(6)   0.0225(7)   0.0013(9)     0.0361     0.00  0.00  0.00
C15    - C16       1.357(2)          0        0        0        0  0.0282(7)   0.0273(7)   0.0009(9)     0.0300     0.00  0.00  0.00
C16    - C17     1.4285(19)          0        0        0        0  0.0168(7)   0.0167(6)   0.0001(9)     0.0100     0.00  0.00  0.00
C17    - C18     1.3940(19)          0        0        0        0  0.0213(6)   0.0208(7)   0.0005(9)     0.0224     0.00  0.00  0.00
C18    - C19     1.3961(19)          0        0        0        0  0.0217(7)   0.0205(6)   0.0012(9)     0.0346     0.00  0.00  0.00
C19    - C20       1.430(2)          0        0        0        0  0.0160(6)   0.0172(7)   0.0012(9)     0.0346     0.00  0.00  0.00
C19    - C24     1.4369(18)          0        0        0        0  0.0132(6)   0.0130(6)   0.0002(8)     0.0141     0.00  0.00  0.00
C20    - C21       1.358(2)          0        0        0        0  0.0280(7)   0.0272(7)  0.0008(10)     0.0283     0.00  0.00  0.00
C21    - C22       1.421(2)          0        0        0        0  0.0253(7)   0.0263(7)  0.0010(10)     0.0316     0.00  0.00  0.00
C22    - C23     1.3614(19)          0        0        0        0  0.0193(7)   0.0191(6)   0.0002(9)     0.0141     0.00  0.00  0.00
C23    - C24     1.4317(18)          0        0        0        0  0.0165(6)   0.0161(6)   0.0004(8)     0.0200     0.00  0.00  0.00

                                                        Sqrt(Sum(DelIJ**2)/Nrb) =     0.0013

                             # - Indicates bonds exceeding the 5.0 sigma test level
```

```
                                                                                         "gliesep200"  PLATON-ADP-Anal  Page   14
===================================================================================================================================
Test Matrix for Rigid-Body Vibrations - /Del(A,B)/ = /Z(A,B)**2 - Z(B,A)**2/ Should be Near Zero (Acta Cryst. A34, 1978, 828)
===================================================================================================================================

Atom-Atom     1  2  3  4  5  6  7  8  9 10 11 12 13 14 15 16 17 18 19 20 21 22 23 24 25 26 27
-----------------------------------------------------------------------------------------------------------------------------------
 1 Si1    -   0 -2  4  3  3  1 -3 -2 -1  1  2  3  2  2  2  3  1  1  0  0  1  1  2  5  4  1  3
 2 N1     -  -2  0  1  0  1  0  2  1  0  3  5  2  1  1  4  6  1  0  2  1  2  2  3  7  5  1 -1
 3 C1     -   4  3  0 -2  2  2  3  3  0 10 11  5  3  3  2  0  4  4  3  2  2  2  2  2  0  2 -1
 4 C2     -   5  3 -1  0  0  2  0  3  2 13 14  3  0  0  2  4  1  2  1  2  1  1  0  6  1  1  2
 5 C3     -   4  3  2 -1  0 -1  0  0  3 13 15  0  2  2  3  6  2  1  3  1  2  1  3  7  3  2  2
 6 C4     -   3 -1  3  2 -1  0  2  1  1  5 11  2  2  0  0  3  1  2  2  1  3  3  4  9  6  3  3
 7 C5     -  -2  3  5  6  5  4  0  1  1  5  3  4  6  8  6  9 11  7  7  5  7  5  6  6  3  7  2
 8 C6     -  -2  3  5  6  5  3  3  0  2  3  2  7  7 11 12 10 11 11  9  7  6  3  2  2  2  5  3
 9 C7     -  -2  3  5  5  4  3  3  3  0  0 -3 -3  4  3  2  3  3  2  2  3  5  4  2  1  4  5  0
10 C8     -   3  3  5  5  4  4  4  4 -2  0  3  0  9  6  0  2  5  7  8 12 14 15 12  9 14 13  8
11 C9     -   3  4  6  6  4  4  4  3 -2  3  0  2  5  3  1  3  2  3  2  4  5  5  4  3  7  6  5
12 C10    -   3  4  7  7  6  5  3  4 -2  2  2  0 14 10  3  4 10 10 13 19 28 35 41 41 32 25  5
13 C11    -   3  3  3  4  4  4  3  5  5  5  6  6  0 -1  2  1  2  1  0  1  1  1  2  1  3  0  0
14 C12    -   4  3  4  5  5  5  3  5  6  6  7  6 -1  0  0  1  3  1 -1  0  1  0  2  1  1  1  1
15 C13    -   4  4  4  5  5  4  4  4  6  6  7  7  2 -1  0 -1  1  1  2  1  1  0  3  2  1  1  6
16 C14    -   5  5  5  6  7  6  5  5  7  8  8  8  4  2 -1  0 -1  1  3  3  1  3  0  0  2  0  7
17 C15    -   6  6  6  7  8  7  5  6  8  9  9  8  4  3  2 -1  0 -1  2  1  1  2  0  0  4  2  1
18 C16    -   6  6  6  7  8  7  5  6  8  8  9  8  4  2  3  2 -1  0  0  0  1  0  3  3  0  0  0
19 C17    -   5  5  5  6  7  6  4  6  7  7  8  7  2 -1  2  3  2 -1  0  0  1  0  2  3  0  0  1
20 C18    -   6  5  5  7  7  7  4  7  7  7  8  7  3  2  4  4  4  2 -1  0 -1  0  3  4  2  0  1
21 C19    -   5  5  5  6  7  6  4  7  6  6  8  7  2  3  4  5  5  4  2 -1  0 -1  1  3  2  0  0
22 C20    -   6  6  6  7  8  7  5  8  7  6  8  7  4  4  6  6  6  5  4  2 -1  0 -1  1  0  0  1
23 C21    -   6  6  6  7  7  7  5  8  7  6  8  7  4  5  6  7  7  6  5  4  2 -1  0 -1  1  1  3
24 C22    -   5  5  5  6  6  6  5  7  6  5  7  6  4  5  6  7  7  6  5  4  3  2 -1  0  0  1  8
25 C23    -   4  4  4  4  5  5  4  6  5  4  6  5  2  4  5  6  6  6  4  4  2  3  2 -1  0  0  8
26 C24    -   4  4  4  5  5  5  3  6  5  5  7  6 -1  2  4  5  5  4  3  2 -1  2  3  2 -1  0  1
27 B1     -   3 -1 -2  3  3  2  3  4  4  4  5  5 -2  3  3  4  5  5  4  4  4  5  5  4  3  3  0

Remarks
-------
- Upper Triangle Entries Represent /Del(A,B)/*1000 Values

- Lower Triangle Entries Represent Distances (A-B) Angstrom

- Negative Entries Indicate Bonded Atoms
```

```
                                                                                         "gliesep200"  PLATON-GEOMETRY  Page   15
===================================================================================================================================
Analysis of Bond Distance and Angle Values - Identification of Chiral Center(s) and Their (R/S)-Configuration (Cahn-Ingold-Prelog)
===================================================================================================================================
    The Following Tests are done. Faults are Marked Under Note
    -- V : Valency Check Fault for H, C
    -- S : Bond Too Short
    -- A : Unusual Bond Angle Values (PLEASE CHECK)
===================================================================================================================================
 *** PLEASE NOTE: R/S ASSIGNMENTS ARE TENTATIVE  *** (CIP Special rules NOT Implemented)
 *** See Angew.Chem.Intern. Ed. Eng.,(1966),5,385 & (1982),21,567 for Authoritative Details for Special Cases

                                                                                    =A.N.G.L.E.S=   =B.O.N.D.S=
Flag Label  - Connected to  (May be Incomplete for Polymeric Structures)          nra min max  Aver  min   max nrb tnr Hyb RS Note
-----------------------------------------------------------------------------------------------------------------------------------
d    Si1    - N1      C5      C6      C7      ------- ------- ------- ------- ------- 6 107 112 109.5 1.767 1.915  4  39 sp3
d    N1     - Si1     C4      B1      ------- ------- ------- ------- ------- ------- 3 112 130 120.0 1.405 1.767  3  38 sp2
d    C1     - C2      B1      H1A     H1B     ------- ------- ------- ------- ------- 6 108 113 109.4 0.990 1.586  4  30 sp3
d    C2     - C1      C3      H2      ------- ------- ------- ------- ------- ------- 3 120 121 120.0 0.950 1.495  3  32 sp2
d    C3     - C2      C4      H3      ------- ------- ------- ------- ------- ------- 3 119 122 120.0 0.950 1.499  3  33 sp2
d    C4     - N1      C3      H4A     H4B     ------- ------- ------- ------- ------- 6 108 114 109.4 0.990 1.499  4  35 sp3
d    C5     - Si1     H5A     H5B     H5C     ------- ------- ------- ------- ------- 6 109 109 109.5 0.980 1.861  4  36 sp3
d    C6     - Si1     H6A     H6B     H6C     ------- ------- ------- ------- ------- 6 109 109 109.5 0.980 1.866  4  36 sp3
d    C7     - Si1     C8      C9      C10     ------- ------- ------- ------- ------- 6 107 114 109.4 1.535 1.915  4  37 sp3
d    C8     - C7      H8A     H8B     H8C     ------- ------- ------- ------- ------- 6 109 109 109.5 0.980 1.535  4  34 sp3
d    C9     - C7      H9A     H9B     H9C     ------- ------- ------- ------- ------- 6 109 109 109.5 0.980 1.538  4  34 sp3
d    C10    - C7      H10A    H10B    H10C    ------- ------- ------- ------- ------- 6 109 109 109.5 0.980 1.539  4  34 sp3
d    C11    - C12     C24     B1      ------- ------- ------- ------- ------- ------- 3 118 122 120.0 1.411 1.595  3  28 sp2
d    C12    - C11     C13     C17     ------- ------- ------- ------- ------- ------- 3 117 122 120.0 1.415 1.439  3  19 sp2
d    C13    - C12     C14     H13     ------- ------- ------- ------- ------- ------- 3 119 122 120.0 0.950 1.430  3  26 sp2
d    C14    - C13     C15     H14     ------- ------- ------- ------- ------- ------- 3 120 120 120.0 0.950 1.422  3  15 sp2
d    C15    - C14     C16     H15     ------- ------- ------- ------- ------- ------- 3 120 120 120.0 0.950 1.422  3  23 sp2
d    C16    - C15     C17     H16     ------- ------- ------- ------- ------- ------- 3 119 121 120.0 0.950 1.429  3  18 sp2
d    C17    - C12     C16     C18     ------- ------- ------- ------- ------- ------- 3 119 122 120.0 1.394 1.439  3  27 sp2
d    C18    - C17     C19     H18     ------- ------- ------- ------- ------- ------- 3 119 121 120.0 0.950 1.396  3  20 sp2
d    C19    - C18     C20     C24     ------- ------- ------- ------- ------- ------- 3 119 122 120.0 1.396 1.437  3  27 sp2
d    C20    - C19     C21     H20     ------- ------- ------- ------- ------- ------- 3 119 121 120.0 0.950 1.430  3  18 sp2
d    C21    - C20     C22     H21     ------- ------- ------- ------- ------- ------- 3 120 120 120.0 0.950 1.421  3  23 sp2
d    C22    - C21     C23     H22     ------- ------- ------- ------- ------- ------- 3 120 120 120.0 0.950 1.421  3  15 sp2
d    C23    - C22     C24     H23     ------- ------- ------- ------- ------- ------- 3 119 122 120.0 0.950 1.432  3  26 sp2
d    C24    - C11     C19     C23     ------- ------- ------- ------- ------- ------- 3 118 121 120.0 1.411 1.437  3  19 sp2
d    B1     - N1      C1      C11     ------- ------- ------- ------- ------- ------- 3 118 123 120.0 1.405 1.595  3  12

cRU  H1A    - C1      ------- ------- ------- ------- ------- ------- ------- ------- 0   0   0   0.0 0.990 0.990  1   6
cRU  H1B    - C1      ------- ------- ------- ------- ------- ------- ------- ------- 0   0   0   0.0 0.990 0.990  1   6
cRU  H2     - C2      ------- ------- ------- ------- ------- ------- ------- ------- 0   0   0   0.0 0.950 0.950  1   7
cRU  H3     - C3      ------- ------- ------- ------- ------- ------- ------- ------- 0   0   0   0.0 0.950 0.950  1   8
cRU  H4A    - C4      ------- ------- ------- ------- ------- ------- ------- ------- 0   0   0   0.0 0.990 0.990  1  10
cRU  H4B    - C4      ------- ------- ------- ------- ------- ------- ------- ------- 0   0   0   0.0 0.990 0.990  1  10

                                                                                         "gliesep200"  PLATON-GEOMETRY  Page   16
===================================================================================================================================
cRU  H5A    - C5      ------- ------- ------- ------- ------- ------- ------- ------- 0   0   0   0.0 0.980 0.980  1  11
cRU  H5B    - C5      ------- ------- ------- ------- ------- ------- ------- ------- 0   0   0   0.0 0.980 0.980  1  11
cRU  H5C    - C5      ------- ------- ------- ------- ------- ------- ------- ------- 0   0   0   0.0 0.980 0.980  1  11
cRU  H6A    - C6      ------- ------- ------- ------- ------- ------- ------- ------- 0   0   0   0.0 0.980 0.980  1  11
```

```
cRU  H6B    - C6       ------- ------- ------- ------- ------- ------- ------- ------- 0   0   0   0.0 0.980 0.980  1  11
cRU  H6C    - C6       ------- ------- ------- ------- ------- ------- ------- ------- 0   0   0   0.0 0.980 0.980  1  11
cRU  H8A    - C8       ------- ------- ------- ------- ------- ------- ------- ------- 0   0   0   0.0 0.980 0.980  1   9
cRU  H8B    - C8       ------- ------- ------- ------- ------- ------- ------- ------- 0   0   0   0.0 0.980 0.980  1   9
cRU  H8C    - C8       ------- ------- ------- ------- ------- ------- ------- ------- 0   0   0   0.0 0.980 0.980  1   9
cRU  H9A    - C9       ------- ------- ------- ------- ------- ------- ------- ------- 0   0   0   0.0 0.980 0.980  1   9
cRU  H9B    - C9       ------- ------- ------- ------- ------- ------- ------- ------- 0   0   0   0.0 0.980 0.980  1   9
cRU  H9C    - C9       ------- ------- ------- ------- ------- ------- ------- ------- 0   0   0   0.0 0.980 0.980  1   9
cRU  H10A   - C10      ------- ------- ------- ------- ------- ------- ------- ------- 0   0   0   0.0 0.980 0.980  1   9
cRU  H10B   - C10      ------- ------- ------- ------- ------- ------- ------- ------- 0   0   0   0.0 0.980 0.980  1   9
cRU  H10C   - C10      ------- ------- ------- ------- ------- ------- ------- ------- 0   0   0   0.0 0.980 0.980  1   9
cRU  H13    - C13      ------- ------- ------- ------- ------- ------- ------- ------- 0   0   0   0.0 0.950 0.950  1   2
cRU  H14    - C14      ------- ------- ------- ------- ------- ------- ------- ------- 0   0   0   0.0 0.950 0.950  1   3
cRU  H15    - C15      ------- ------- ------- ------- ------- ------- ------- ------- 0   0   0   0.0 0.950 0.950  1   1
cRU  H16    - C16      ------- ------- ------- ------- ------- ------- ------- ------- 0   0   0   0.0 0.950 0.950  1   4
cRU  H18    - C18      ------- ------- ------- ------- ------- ------- ------- ------- 0   0   0   0.0 0.950 0.950  1   5
cRU  H20    - C20      ------- ------- ------- ------- ------- ------- ------- ------- 0   0   0   0.0 0.950 0.950  1   4
cRU  H21    - C21      ------- ------- ------- ------- ------- ------- ------- ------- 0   0   0   0.0 0.950 0.950  1   1
cRU  H22    - C22      ------- ------- ------- ------- ------- ------- ------- ------- 0   0   0   0.0 0.950 0.950  1   3
cRU  H23    - C23      ------- ------- ------- ------- ------- ------- ------- ------- 0   0   0   0.0 0.950 0.950  1   2

                                                                                      "gliesep200"  PLATON-GEOMETRY  Page   17
=================================================================================================================================
Analysis of the IntraMolecular Geometry in Terms of Unique Molecule(s)/Ions, with Bond Criterium: d(i-j) < R(i) + R(j) + Tol
---------------------------------------------------------------------------------------------------------------------------------
-- Tol = 0.40 Ang. for Normal Bonds + 0.70 for (Earth)alkali-NonMetal Contacts and adjusted by -.40 Ang. for Metal-Metal Distances
-- The Bond Distance and Angle su's have been Incremented to Include the Effect of the Unit-cell su.
   (Rel.Error in Dist. 0.0000 Ang. , Abs. Angle Error 0.000 Deg.)
-- Bonds below with '>' or '<' Substituted for '-' have Distances that Deviate from Expected Values(Based on the hybridisations).
---------------------------------------------------------------------------------------------------------------------------------

Bond Lengths (Angstrom). - (Bonds are ordered on the first label, left to right and  top to bottom) - su in last digit in ().
=================================================================================================================================
 Si1    -  N1       1.7669(12)    Si1    -  C5       1.8606(14)    Si1    -  C6       1.8655(14)    Si1    -  C7       1.9150(15)
 N1     -  C4       1.4962(16)    N1     -  B1       1.4053(18)    C1     -  C2       1.4946(19)    C1     -  B1         1.586(2)
 C2     -  C3         1.328(2)    C3     -  C4       1.4989(19)    C7     -  C8         1.535(2)    C7     -  C9         1.538(2)
 C7     -  C10        1.539(2)    C11    -  C12      1.4145(17)    C11    -  C24      1.4109(17)    C11    -  B1       1.5946(19)
 C12    -  C13      1.4300(18)    C12    -  C17      1.4395(18)    C13    -  C14      1.3647(19)    C14    -  C15        1.422(2)
 C15    -  C16        1.357(2)    C16    -  C17      1.4285(19)    C17    -  C18      1.3940(19)    C18    -  C19      1.3961(19)
 C19    -  C20        1.430(2)    C19    -  C24      1.4369(18)    C20    -  C21        1.358(2)    C21    -  C22        1.421(2)
 C22    -  C23      1.3614(19)    C23    -  C24      1.4317(18)

 C1     -  H1A            0.99    C1     -  H1B            0.99    C2     -  H2             0.95    C3     -  H3             0.95
 C4     -  H4A            0.99    C4     -  H4B            0.99    C5     -  H5A            0.98    C5     -  H5B            0.98
 C5     -  H5C            0.98    C6     -  H6A            0.98    C6     -  H6B            0.98    C6     -  H6C            0.98
 C8     -  H8A            0.98    C8     -  H8B            0.98    C8     -  H8C            0.98    C9     -  H9A            0.98
 C9     -  H9B            0.98    C9     -  H9C            0.98    C10    -  H10A           0.98    C10    -  H10B           0.98
 C10    -  H10C           0.98    C13    -  H13            0.95    C14    -  H14            0.95    C15    -  H15            0.95
 C16    -  H16            0.95    C18    -  H18            0.95    C20    -  H20            0.95    C21    -  H21            0.95
 C22    -  H22            0.95    C23    -  H23            0.95

Bond/Valence Angles (Degrees) - (Angles are ordered on the middle label, left to right and top to bottom) - su in last digit in ().
=================================================================================================================================
 N1     -  Si1    -  C5        112.07(6)     N1     -  Si1    -  C6        108.12(7)     N1     -  Si1    -  C7        109.65(6)
 C5     -  Si1    -  C6        107.16(7)     C5     -  Si1    -  C7        108.35(7)     C6     -  Si1    -  C7        111.49(7)
 Si1    -  N1     -  C4        112.36(9)     Si1    -  N1     -  B1        130.20(9)     C4     -  N1     -  B1       117.44(11)
 C2     -  C1     -  B1       113.29(12)     C1     -  C2     -  C3       120.61(12)     C2     -  C3     -  C4       121.63(12)
 N1     -  C4     -  C3       113.90(11)     Si1    -  C7     -  C8       109.94(10)     Si1    -  C7     -  C9       114.47(11)
 Si1    -  C7     -  C10      108.10(11)     C8     -  C7     -  C9       109.15(13)     C8     -  C7     -  C10      108.37(14)
 C9     -  C7     -  C10      106.60(13)     C12    -  C11    -  C24      118.47(11)     C12    -  C11    -  B1       119.92(11)
 C24    -  C11    -  B1       121.51(11)     C11    -  C12    -  C13      121.88(11)     C11    -  C12    -  C17      120.62(11)
 C13    -  C12    -  C17      117.50(11)     C12    -  C13    -  C14      121.65(12)     C13    -  C14    -  C15      120.36(13)
 C14    -  C15    -  C16      120.11(13)     C15    -  C16    -  C17      121.34(13)     C12    -  C17    -  C16      119.00(12)
 C12    -  C17    -  C18      119.46(12)     C16    -  C17    -  C18      121.54(12)     C17    -  C18    -  C19      121.15(12)
 C18    -  C19    -  C20      121.59(12)     C18    -  C19    -  C24      119.28(12)     C20    -  C19    -  C24      119.13(12)
 C19    -  C20    -  C21      120.99(13)     C20    -  C21    -  C22      120.28(13)     C21    -  C22    -  C23      120.48(13)
 C22    -  C23    -  C24      121.53(13)     C11    -  C24    -  C19      120.97(11)     C11    -  C24    -  C23      121.43(11)
 C19    -  C24    -  C23      117.59(11)     N1     -  B1     -  C1       118.97(12)     N1     -  B1     -  C11      123.12(11)
 C1     -  B1     -  C11      117.89(11)

 C2     -  C1     -  H1A             109     C2     -  C1     -  H1B             109     B1     -  C1     -  H1A             109

                                                                                      "gliesep200"  PLATON-GEOMETRY  Page   18
=================================================================================================================================
 B1     -  C1     -  H1B             109     H1A    -  C1     -  H1B             108     C1     -  C2     -  H2              120
 C3     -  C2     -  H2              120     C2     -  C3     -  H3              119     C4     -  C3     -  H3              119
 N1     -  C4     -  H4A             109     N1     -  C4     -  H4B             109     C3     -  C4     -  H4A             109
 C3     -  C4     -  H4B             109     H4A    -  C4     -  H4B             108     Si1    -  C5     -  H5A             109
 Si1    -  C5     -  H5B             109     Si1    -  C5     -  H5C             109     H5A    -  C5     -  H5B             109
 H5A    -  C5     -  H5C             109     H5B    -  C5     -  H5C             109     Si1    -  C6     -  H6A             109
 Si1    -  C6     -  H6B             109     Si1    -  C6     -  H6C             109     H6A    -  C6     -  H6B             109
 H6A    -  C6     -  H6C             109     H6B    -  C6     -  H6C             109     C7     -  C8     -  H8A             109
 C7     -  C8     -  H8B             109     C7     -  C8     -  H8C             109     H8A    -  C8     -  H8B             109
 H8A    -  C8     -  H8C             109     H8B    -  C8     -  H8C             109     C7     -  C9     -  H9A             109
 C7     -  C9     -  H9B             109     C7     -  C9     -  H9C             109     H9A    -  C9     -  H9B             109
 H9A    -  C9     -  H9C             109     H9B    -  C9     -  H9C             109     C7     -  C10    -  H10A            109
 C7     -  C10    -  H10B            109     C7     -  C10    -  H10C            109     H10A   -  C10    -  H10B            109
 H10A   -  C10    -  H10C            109     H10B   -  C10    -  H10C            109     C12    -  C13    -  H13             119
 C14    -  C13    -  H13             119     C13    -  C14    -  H14             120     C15    -  C14    -  H14             120
 C14    -  C15    -  H15             120     C16    -  C15    -  H15             120     C15    -  C16    -  H16             119
 C17    -  C16    -  H16             119     C17    -  C18    -  H18             119     C19    -  C18    -  H18             119
 C19    -  C20    -  H20             120     C21    -  C20    -  H20             119     C20    -  C21    -  H21             120
 C22    -  C21    -  H21             120     C21    -  C22    -  H22             120     C23    -  C22    -  H22             120
 C22    -  C23    -  H23             119     C24    -  C23    -  H23             119

Torsion/Dihedral Angles (Deg.) - Klyne & Prelog Convention (Dunitz, p241) - (Excl. Minor Disorder & Embedded Bond Angl. > 160. Deg.)
=================================================================================================================================
 C5     Si1    N1     C4          177.56(9)    C5     Si1    N1     B1           -3.02(14)    C6     Si1    N1     C4           59.67(10)
 C6     Si1    N1     B1        -120.90(12)    C7     Si1    N1     C4          -62.08(10)    C7     Si1    N1     B1          117.35(12)
 N1     Si1    C7     C8         -37.56(12)    N1     Si1    C7     C9           85.70(12)    N1     Si1    C7     C10       -155.69(11)
 C5     Si1    C7     C8          85.04(12)    C5     Si1    C7     C9         -151.70(11)    C5     Si1    C7     C10        -33.08(13)
 C6     Si1    C7     C8        -157.27(10)    C6     Si1    C7     C9          -34.01(13)    C6     Si1    C7     C10         84.61(13)
 Si1    N1     C4     C3         143.11(10)    B1     N1     C4     C3          -36.39(16)    Si1    N1     B1     C1         -168.86(9)
 Si1    N1     B1     C11         12.59(19)    C4     N1     B1     C1           10.54(17)    C4     N1     B1     C11       -168.01(11)
 B1     C1     C2     C3         -25.45(18)    C2     C1     B1     N1           20.11(17)    C2     C1     B1     C11       -161.26(11)
 C1     C2     C3     C4            -0.7(2)    C2     C3     C4     N1           32.76(19)    C24    C11    C12    C13       -178.98(11)
 C24    C11    C12    C17          1.46(17)    B1     C11    C12    C13          -2.48(17)    B1     C11    C12    C17        177.96(11)
 C12    C11    C24    C19         -2.56(17)    C12    C11    C24    C23         178.93(11)    B1     C11    C24    C19       -179.00(11)
 B1     C11    C24    C23          2.49(18)    C12    C11    B1     N1           83.53(16)    C12    C11    B1     C1         -95.04(14)
 C24    C11    B1     N1        -100.08(15)    C24    C11    B1     C1           81.36(15)    C11    C12    C13    C14        178.39(12)
 C17    C12    C13    C14         -2.03(18)    C11    C12    C17    C16        -178.74(12)    C11    C12    C17    C18          0.83(19)
 C13    C12    C17    C16          1.68(18)    C13    C12    C17    C18        -178.75(12)    C12    C13    C14    C15            0.7(2)
 C13    C14    C15    C16            1.2(2)    C14    C15    C16    C17            -1.5(2)    C15    C16    C17    C12            0.0(2)
 C15    C16    C17    C18       -179.53(14)    C12    C17    C18    C19            -2.1(2)    C16    C17    C18    C19        177.49(13)
 C17    C18    C19    C20       -178.58(13)    C17    C18    C19    C24             1.0(2)    C18    C19    C20    C21        179.64(14)
 C24    C19    C20    C21            0.1(2)    C18    C19    C24    C11           1.38(19)    C18    C19    C24    C23        179.94(14)
 C20    C19    C24    C11       -179.04(12)    C20    C19    C24    C23          -0.48(19)    C19    C20    C21    C22            0.1(2)
```

C20	C21	C22	C23	0.2(2)	C21	C22	C23	C24	-0.6(2)	C22	C23	C24	C11	179.33(13)
C22	C23	C24	C19	0.77(19)										
N1	Si1	C5	H5A	-55	N1	Si1	C5	H5B	-175	N1	Si1	C5	H5C	65
C6	Si1	C5	H5A	63	C6	Si1	C5	H5B	-57	C6	Si1	C5	H5C	-177

"gliesep200" PLATON-GEOMETRY Page 19

C7	Si1	C5	H5A	-176	C7	Si1	C5	H5B	64	C7	Si1	C5	H5C	-56
N1	Si1	C6	H6A	-59	N1	Si1	C6	H6B	-179	N1	Si1	C6	H6C	61
C5	Si1	C6	H6A	-180	C5	Si1	C6	H6B	60	C5	Si1	C6	H6C	-60
C7	Si1	C6	H6A	62	C7	Si1	C6	H6B	-58	C7	Si1	C6	H6C	-178
Si1	N1	C4	H4A	22	Si1	N1	C4	H4B	-95	B1	N1	C4	H4A	-158
B1	N1	C4	H4B	85	B1	C1	C2	H2	155	H1A	C1	C2	C3	-147
H1A	C1	C2	H2	33	H1B	C1	C2	C3	96	H1B	C1	C2	H2	-84
H1A	C1	B1	N1	141	H1A	C1	B1	C11	-40	H1B	C1	B1	N1	-101
H1B	C1	B1	C11	77	C1	C2	C3	H3	179	H2	C2	C3	C4	179
H2	C2	C3	H3	-1	C2	C3	C4	H4A	154	C2	C3	C4	H4B	-89
H3	C3	C4	N1	-147	H3	C3	C4	H4A	-26	H3	C3	C4	H4B	91
Si1	C7	C8	H8A	-55	Si1	C7	C8	H8B	-175	Si1	C7	C8	H8C	65
C9	C7	C8	H8A	179	C9	C7	C8	H8B	59	C9	C7	C8	H8C	-61
C10	C7	C8	H8A	63	C10	C7	C8	H8B	-57	C10	C7	C8	H8C	-177
Si1	C7	C9	H9A	-67	Si1	C7	C9	H9B	173	Si1	C7	C9	H9C	53
C8	C7	C9	H9A	57	C8	C7	C9	H9B	-63	C8	C7	C9	H9C	177
C10	C7	C9	H9A	174	C10	C7	C9	H9B	54	C10	C7	C9	H9C	-66
Si1	C7	C10	H10A	-57	Si1	C7	C10	H10B	-177	Si1	C7	C10	H10C	63
C8	C7	C10	H10A	-176	C8	C7	C10	H10B	64	C8	C7	C10	H10C	-56
C9	C7	C10	H10A	67	C9	C7	C10	H10B	-53	C9	C7	C10	H10C	-173
C11	C12	C13	H13	-2	C17	C12	C13	H13	178	C12	C13	C14	H14	-179
H13	C13	C14	C15	-179	H13	C13	C14	H14	1	C13	C14	C15	H15	-179
H14	C14	C15	C16	-179	H14	C14	C15	H15	1	C14	C15	C16	H16	179
H15	C15	C16	C17	179	H15	C15	C16	H16	-1	H16	C16	C17	C12	-180
H16	C16	C17	C18	0	C12	C17	C18	H18	178	C16	C17	C18	H18	-3
H18	C18	C19	C20	1	H18	C18	C19	C24	-179	C18	C19	C20	H20	0
C24	C19	C20	H20	-180	C19	C20	C21	H21	-180	H20	C20	C21	C22	-180
H20	C20	C21	H21	0	C20	C21	C22	H22	-180	H21	C21	C22	C23	-180
H21	C21	C22	H22	0	C21	C22	C23	H23	179	H22	C22	C23	C24	179
H22	C22	C23	H23	-1	H23	C23	C24	C11	-1	H23	C23	C24	C19	-179

"gliesep200" PLATON-GEOMETRY Page 20

Statistics of Bond Length per Bond Type (NOTE: A Indicates 10 Occurrences, B Indicates 11, Etc. and * more than 35)

```
Bond Type   Nr    B.P.  d(min)  d(max)   d(av) Sumrad Note  0.0       0.5       1.0       1.5       2.0       2.5       3.0 Angstrom
---------------------------------------------------------- I-I-I-I-I-I-I-I-I-I-I-I-I-I-I-I-I-I-I-I-I-I-I-I-I-I-I-I-I-I-I-I-I-I-I-I-I
Si  --  N    1  0.0012  1.7669  1.7669  1.7669   1.88        ....................................1...................................

Si  --  C4   3  0.0014  1.8606  1.9150  1.8804   1.88        .....................................21.................................

 N3 --  C4   1  0.0016  1.4962  1.4962  1.4962   1.36        ...............................1........................................

 N  --  B    1  0.0018  1.4053  1.4053  1.4053   1.51        .............................1..........................................

 C4 --  C3   1  0.0019  1.4946  1.4946  1.4946   1.36        ...............................1........................................

 C  --  B    2  0.0020  1.5860  1.5946  1.5903   1.51        .................................2......................................

 C3 --  C3  17  0.0019  1.3280  1.4395  1.4014   1.36        ...........................566..........................................

 C3 --  C4   1  0.0019  1.4989  1.4989  1.4989   1.36        ...............................1........................................

 C4 --  C4   3  0.0020  1.5350  1.5390  1.5373   1.36        ................................3.......................................

 C  --  H   30          0.9500  0.9900  0.9703   1.03        ...................BJ...................................................
```

Selected Bond Lengths (Angstrom) - see M.F.C. Ladd & R.A. Palmer, Structure Determination by X-ray Crystallography (1985)

```
                                                  Formal single bonds
                                                  -------------------
C4-C4 1.54   C4-C3 1.52   C4-C2 1.46   C4-N3 1.47   C4-N2 1.47   C4-O2 1.43   C3-C3 1.46   C3-C2 1.45   C3-N3 1.40   C3-N2 1.40
C3-O2 1.36   C2-C2 1.38   C2-N3 1.33   C2-N2 1.33   C2-O2 1.36   N3-N3 1.45   N3-N2 1.45   N3-O2 1.36   N2-N2 1.45   N2-O2 1.41

                                                  Formal double bonds
                                                  -------------------
C3-C3 1.34   C3-C2 1.31   C3-N2 1.32   C3-O1 1.22   C2-C2 1.28   C2-N2 1.32   C2-O1 1.16   N3-O1 1.24   N2-N2 1.25   N2-O1 1.21

Formal triple bonds                   Aromatic bonds
-------------------                   --------------
C2-C2 1.20   C2-N1 1.16   C3-C3 1.40   C2-N2 1.34   N2-N2 1.35
```

The notation in the table indicates the connectivity of the atoms

For more detailed standard bond distance tabulations see: J. Chem. Soc. Perkin II, (1987), S1-S19;
J. Chem. Soc. Dalton Trans. (1989), S1 - S83 or International Tables C, (1992), 707-791.

"gliesep200" PLATON-GEOMETRY Page 21

Automatic Search for Rings (3 to 24-Membered) and Planes Determined by 4 or More Connected Atoms (with Deviation < 0.10 Ang.)

```
Least-Squares Planes - P*X+Q*Y+R*Z=S   ::  First Line Orthogonal(XO,YO,ZO), Second Line Fractional(X,Y,Z)
=====================================      Ring/Plan/Resd/Lspl N Indicates that the Ring/Plane/Residue Involves N Atoms
                                           Sigref - R.M.S-Error of the Contributing Atoms
  The Deviation D of an Atom with          Sigpln - Sqrt(Sum(j=1:N)(D(j)**2/(N-3))
                                           Chisq  - Chi-Squared = Sum(j=1:N)(D(j)**2)/Sigref**2
  Fractional Coordinates X,Y,Z may be      Pl.Hyp. - Result of the Chi.Sq. Test for Planarity (See Stout & Jensen, p424)

  Calculated via Substitution in
                                           **** - Atoms Deviating by More Than 1.5 Angstrom and Hydrogen Atoms are NOT Listed
  D = P*X + Q*Y + R*Z - S (2nd Line)       Note - Weights : UNIT
                                                - Deviations from planes are in Angstrom Units
                                                - The Plane determining Atoms have been Marked #
                                                - DISTANCES TO PLANES ROUNDED TO 3 DECIMALS !!(Use Graphical Interface for more)
----------------------------------------------------------------------------------------------------------------------------------
Nr  1         P             Q             R             S     Sigref  0.001     Sigpln  0.233     Chisq    92121.4     Pl.Hyp.    P<5
----------------------------------------------------------------------------------------------------------------------------------
Ring     0.7430(4)    -0.0819(6)    -0.6643(4)    -6.265(10)  #N1     -0.156(1)  #C1      0.206(1)  #C2     -0.125(1)  #C3     -0.110(1)
A  6      9.904(5)     -0.912(6)   -18.996(12)    -6.265(10)  #C4      0.253(1)  #B1     -0.068(1)   Si1    -0.700(1)   C5     -1.137(1)
                                                               C6      0.706(2)   C11    -0.198(1)   C12     0.931(1)   C17     0.842(1)
                                                               C18    -0.375(1)   C19    -1.499(1)   C24    -1.401(1)
----------------------------------------------------------------------------------------------------------------------------------
Nr  2         P             Q             R             S     Sigref  0.001     Sigpln  0.014     Chisq      375.0     Pl.Hyp.    P<5
----------------------------------------------------------------------------------------------------------------------------------
```

```
Ring      0.4431(5)     0.2277(5)     0.8671(3)     19.111(2) #C11      0.013(1) #C12     -0.001(1) #C17     -0.011(1) #C18      0.012(1)
A  6      5.907(6)      2.536(6)     24.796(8)     19.111(2) #C19      0.001(1) #C24     -0.013(1)  N1       1.121(1)  C1      -1.436(1)
                                                               C2      -1.335(1)  C3      -0.244(1)  C4       0.917(1)  C13      0.002(1)
                                                               C14     -0.045(1)  C15     -0.085(1)  C16     -0.054(1)  C20     -0.006(1)
                                                               C21     -0.024(1)  C22     -0.035(1)  C23     -0.023(1)  B1      -0.025(1)
----------------------------------------------------------------------------------------------------------------------------------------
Nr  3        P             Q             R             S     Sigref  0.001     Sigpln  0.012     Chisq      227.9     Pl.Hyp.    P<5
----------------------------------------------------------------------------------------------------------------------------------------
Ring      0.4612(5)     0.2473(5)     0.8521(3)     19.183(2) #C12     -0.012(1) #C13      0.009(1) #C14      0.003(1) #C15     -0.010(1)
A  6      6.148(7)      2.754(6)     24.369(8)     19.183(2) #C16      0.006(1) #C17      0.005(1)  N1       1.026(1)  C2      -1.473(1)
                                                               C3      -0.389(1)  C4       0.800(1)  C11     -0.040(1)  C18      0.013(1)
                                                               C19     -0.041(1)  C20     -0.064(1)  C21     -0.123(1)  C22     -0.161(1)
                                                               C23     -0.135(1)  C24     -0.082(1)  B1      -0.109(1)
----------------------------------------------------------------------------------------------------------------------------------------
Nr  4        P             Q             R             S     Sigref  0.001     Sigpln  0.003     Chisq       15.8     Pl.Hyp.    P<5
----------------------------------------------------------------------------------------------------------------------------------------
Ring      0.4355(5)     0.2207(6)     0.8727(3)     19.098(3) #C19      0.001(1) #C20      0.001(1) #C21     -0.001(1) #C22     -0.002(1)
A  6      5.805(7)      2.457(6)     24.958(8)     19.098(3) #C23      0.004(1) #C24     -0.003(1)  N1       1.139(1)  C1      -1.416(1)
                                                               C2      -1.301(1)  C3      -0.208(1)  C4       0.941(1)  C11      0.017(1)
                                                               C12     -0.015(1)  C13     -0.019(1)  C14     -0.082(1)  C15     -0.132(1)
                                                               C16     -0.094(1)  C17     -0.034(1)  C18     -0.005(1)  B1      -0.011(1)

                                                                                           "gliesep200"  PLATON-GEOMETRY  Page   22
========================================================================================================================================
----------------------------------------------------------------------------------------------------------------------------------------
Nr  5        P             Q             R             S     Sigref  0.001     Sigpln  0.023     Chisq     2070.9     Pl.Hyp.    P<5
----------------------------------------------------------------------------------------------------------------------------------------
Ring      0.4529(2)     0.2377(4)     0.8593(1)   19.1331(17) #C11      0.002(1) #C12      0.010(1) #C13      0.023(1) #C14     -0.003(1)
A 10      6.037(3)      2.647(4)     24.574(4)   19.1331(17) #C15     -0.029(1) #C16     -0.006(1) #C17      0.014(1) #C18      0.028(1)
                                                              #C19     -0.005(1) #C24     -0.033(1)  N1       1.089(1)  C1      -1.471(1)
                                                               C2      -1.390(1)  C3      -0.302(1)  C4       0.873(1)  C20     -0.021(1)
                                                               C21     -0.061(1)  C22     -0.085(1)  C23     -0.065(1)  B1      -0.052(1)
----------------------------------------------------------------------------------------------------------------------------------------
Nr  6        P             Q             R             S     Sigref  0.001     Sigpln  0.012     Chisq      574.9     Pl.Hyp.    P<5
----------------------------------------------------------------------------------------------------------------------------------------
Ring      0.4381(2)     0.2248(4)     0.8704(1)   19.1042(19) #C11      0.019(1) #C12     -0.005(1) #C17     -0.019(1) #C18      0.009(1)
A 10      5.840(3)      2.503(4)     24.890(4)   19.1042(19) #C19      0.008(1) #C20      0.006(1) #C21     -0.003(1) #C22     -0.009(1)
                                                              #C23     -0.003(1) #C24     -0.002(1)  N1       1.132(1)  C1      -1.423(1)
                                                               C2      -1.316(1)  C3      -0.225(1)  C4       0.930(1)  C13     -0.008(1)
                                                               C14     -0.064(1)  C15     -0.108(1)  C16     -0.071(1)  B1      -0.016(1)
----------------------------------------------------------------------------------------------------------------------------------------
Nr  7        P             Q             R             S     Sigref  0.001     Sigpln  0.028     Chisq     4577.7     Pl.Hyp.    P<5
----------------------------------------------------------------------------------------------------------------------------------------
Ring      0.4457(1)     0.2327(3)     0.8644(1)   19.1084(15) #C11      0.026(1) #C12      0.019(1) #C13      0.024(1) #C14     -0.015(1)
A 14    5.9408(19)      2.591(4)     24.720(3)   19.1084(15) #C15     -0.049(1) #C16     -0.018(1) #C17      0.016(1) #C18      0.038(1)
                                                              #C19      0.019(1) #C20      0.011(1) #C21     -0.015(1) #C22     -0.033(1)
                                                              #C23     -0.020(1) #C24     -0.002(1)  N1       1.122(1)  C1      -1.436(1)
                                                               C2      -1.344(1)  C3      -0.255(1)  C4       0.911(1)  B1      -0.021(1)
----------------------------------------------------------------------------------------------------------------------------------------
Nr  8        P             Q             R             S     Sigref  0.001     Sigpln  1.516     Chisq  999999.9     Pl.Hyp.
----------------------------------------------------------------------------------------------------------------------------------------
Resd      0.4489(4)     0.6193(2)     0.6442(2)   18.8114(17) #Si1      1.201(1) #N1      -0.512(1) #C1      -2.814(1) #C2      -3.379(1)
A 27      5.983(5)    6.8973(19)     18.421(6)   18.8114(17) #C3      -2.662(1) #C4      -1.275(1) #C5       2.156(1) #C6       1.920(2)
                                                              #C7       1.392(1) #C8       0.347(2) #C9       1.287(2) #C10      2.791(2)
                                                              #C11     -0.526(1) #C12     -0.084(1) #C13     -0.201(1) #C14      0.198(1)
                                                              #C15      0.759(1) #C16      0.922(1) #C17      0.513(1) #C18      0.671(1)
                                                              #C19      0.221(1) #C20      0.350(1) #C21     -0.098(1) #C22     -0.707(1)
                                                              #C23     -0.843(1) #C24     -0.396(1) #B1      -1.230(1)
----------------------------------------------------------------------------------------------------------------------------------------
Nr  9        P             Q             R             S     Sigref  0.001     Sigpln  0.028     Chisq     1136.0     Pl.Hyp.    P<5
----------------------------------------------------------------------------------------------------------------------------------------
Plan      0.8373(3)     0.2046(5)    -0.5070(5)    -1.376(10) #Si1     -0.028(1) #N1      -0.002(1) #C4       0.018(1) #C5       0.021(1)
A  5     11.161(5)      2.279(6)   -14.499(13)    -1.376(10) #B1      -0.009(1)  C1      -0.250(1)  C2      -0.905(1)  C3      -0.785(1)
                                                               C11      0.247(1)  C18      0.743(1)  C19     -0.574(1)  C24     -0.819(1)
----------------------------------------------------------------------------------------------------------------------------------------
Nr 10        P             Q             R             S     Sigref  0.001     Sigpln  0.005     Chisq       13.5     Pl.Hyp.    P<5
----------------------------------------------------------------------------------------------------------------------------------------
Plan      0.6317(6)   -0.3292(10)    -0.7019(4)    -8.524(12) #C1      -0.001(1) #C2       0.003(1) #C3      -0.003(1) #C4       0.002(1)
A  4      8.420(8)    -3.666(11)   -20.071(13)    -8.524(12)  N1      -0.728(1)  C6      -0.357(2)  C11     -1.120(1)  C12     -0.257(1)
                                                               C13      1.032(1)  C15      1.478(1)  C16      0.251(1)  C17     -0.659(1)
                                                               B1      -0.639(1)

                                                                                           "gliesep200"  PLATON-GEOMETRY  Page   23
========================================================================================================================================
----------------------------------------------------------------------------------------------------------------------------------------
Nr 11        P             Q             R             S     Sigref  0.001     Sigpln  0.027     Chisq     4703.5     Pl.Hyp.    P<5
----------------------------------------------------------------------------------------------------------------------------------------
Plan      0.4461(1)     0.2303(3)     0.8649(1)   19.0969(12) #C11      0.030(1) #C12      0.022(1) #C13      0.028(1) #C14     -0.013(1)
A 15    5.9455(19)      2.565(3)     24.733(3)   19.0969(12) #C15     -0.050(1) #C16     -0.021(1) #C17      0.015(1) #C18      0.035(1)
                                                              #C19      0.018(1) #C20      0.008(1) #C21     -0.016(1) #C22     -0.030(1)
                                                              #C23     -0.016(1) #C24      0.001(1) #B1      -0.013(1)  N1       1.133(1)
                                                               C1      -1.426(1)  C2      -1.330(1)  C3      -0.240(1)  C4       0.925(1)
----------------------------------------------------------------------------------------------------------------------------------------
Nr 12        P             Q             R             S     Sigref  0.001     Sigpln  0.009     Chisq       55.3     Pl.Hyp.    P<5
----------------------------------------------------------------------------------------------------------------------------------------
Plan      0.8198(4)     0.0342(6)    -0.5716(5)    -3.555(13) #N1       0.003(1) #C1       0.003(1) #C11      0.003(1) #B1      -0.008(1)
A  4     10.928(5)      0.381(7)   -16.346(16)    -3.555(13)  Si1     -0.265(1)  C2      -0.449(1)  C3      -0.332(1)  C4       0.267(1)
                                                               C5      -0.520(1)  C6       1.263(1)  C12      1.227(1)  C17      1.259(1)
                                                               C18      0.067(1)  C19     -1.153(1)  C24     -1.180(1)

(Acute) Angles (Degrees) Between Planes (Numbers I,J from List Above)
----------------------------------------------------------------------------------------------------------------------------------------
 1, 2=   74.61(6)   1, 3=   75.90(6)   1, 4=   74.09(6)   1, 5=   75.30(5)   1, 6=   74.27(5)   1, 7=   74.81(5)   1, 8=   81.66(5)
 1, 9=   19.58(6)   1,10=   15.74(8)   1,11=   74.82(5)   1,12=    9.59(7)   2, 3=    1.75(6)   2, 4=    0.68(6)   2, 5=    0.92(5)
 2, 6=    0.38(5)   2, 7=    0.36(5)   2, 8=   26.05(5)   2, 9=   88.74(6)   2,10=   66.20(8)   2,11=    0.26(5)   2,12=   82.85(7)
 3, 4=    2.43(6)   3, 5=    0.84(5)   3, 6=    2.12(5)   3, 7=    1.41(5)   3, 8=   24.62(5)   3, 9=   89.73(6)   3,10=   67.16(8)
 3,11=    1.49(5)   3,12=   84.23(7)   4, 5=    1.59(5)   4, 6=    0.31(5)   4, 7=    1.02(5)   4, 8=   26.58(5)   4, 9=   88.13(6)
 4,10=   65.79(9)   4,11=    0.94(5)   4,12=   82.28(7)   5, 6=    1.29(4)   5, 7=    0.58(3)   5, 8=   25.31(4)   5, 9=   89.55(5)
 5,10=   66.72(8)   5,11=    0.66(3)   5,12=   83.58(6)   6, 7=    0.72(3)   6, 8=   26.30(4)   6, 9=   88.37(5)   6,10=   65.91(8)
 6,11=    0.64(3)   6,12=   82.49(6)   7, 8=   25.71(3)   7, 9=   89.00(5)   7,10=   66.31(7)   7,11=    0.14(3)   7,12=   83.07(6)
 8, 9=   79.86(5)   8,10=   68.14(8)   8,11=   25.85(3)   8,12=   88.80(6)   9,10=   35.17(8)   9,11=   88.98(5)   9,12=   10.51(7)
10,11=   66.36(7)  10,12=   24.80(9)  11,12=   83.06(6)


(Acute) Angles (Degrees) Between Axes, Lines, and Bonds with L.S.-Planes
----------------------------------------------------------------------------------------------------------------------------------------
 Bond  /  Plane NM --> M      1          2          3          4          5          6          7          8          9         10
----------------------------------------------------------------------------------------------------------------------------------------
Axes O --->a  / 0M =    47.99(5),  26.30(4),  27.47(4),  25.82(5),  26.93(3),  25.98(3),  26.47(2),  26.67(3),  56.86(4),  39.18(7),
                1M =    26.49(2),  55.07(5),
           b  / 0M =     4.70(5),  13.16(4),  14.32(4),  12.75(5),  13.75(3),  12.99(3),  13.46(2),  38.27(3),  11.81(4),  19.22(7),
                1M =    13.32(2),   1.96(5),
           c  / 0M =    41.63(5),  60.12(4),  58.45(4),  60.78(5),  59.24(3),  60.50(3),  59.82(2),  40.10(3),  30.47(4),  44.58(7),
                1M =    59.87(2),  34.86(5),
           a* / 0M =    47.99(5),  26.30(4),  27.47(4),  25.82(5),  26.93(3),  25.98(3),  26.47(2),  26.67(3),  56.86(4),  39.18(7),
                1M =    26.49(2),  55.07(5),
           b* / 0M =     4.70(5),  13.16(4),  14.32(4),  12.75(5),  13.75(3),  12.99(3),  13.46(2),  38.27(3),  11.81(4),  19.22(7),
```

```
                 1M =    13.32(2),   1.96(5),
            c* / 0M =    41.63(5),  60.12(4),  58.45(4),  60.78(5),  59.24(3),  60.50(3),  59.82(2),  40.10(3),  30.47(4),  44.58(7),
                 1M =    59.87(2),  34.86(5),
Si1   -N1      / 0M =    17.93(6),  75.50(6),  76.31(6),  75.18(6),  75.92(5),  75.39(5),  75.75(4),  75.83(5),   0.83(6),  29.49(8),

                                                                                                 "gliesep200"  PLATON-GEOMETRY  Page   24
=====================================================================================================================================
                 1M =    75.61(4),   8.71(7),
      -C5      / 0M =    13.59(6),   7.57(6),   8.46(6),   7.26(6),   8.02(5),   7.46(5),   7.82(5),  30.89(5),   1.48(6),  26.99(8),
                 1M =     7.68(5),   7.89(7),
      -C6      / 0M =    48.89(6),  24.38(6),  25.41(6),  23.94(6),  24.94(5),  24.08(5),  24.51(5),  22.65(5),  55.09(6),  41.70(8),
                 1M =    24.55(5),  54.95(7),
      -C7      / 0M =    53.92(6),  26.39(6),  24.65(6),  27.07(6),  25.48(5),  26.77(5),  26.06(5),   5.71(5),  56.40(6),  43.97(8),
                 1M =    26.13(5),  54.07(7),
N1    -C4      / 0M =    15.86(8),   7.87(7),   8.70(8),   7.59(8),   8.28(7),   7.78(7),   8.11(6),  30.68(7),   0.79(8),  29.19(9),
                 1M =     7.97(6),  10.17(8),
      -B1      / 0M =     3.59(9),  54.68(8),  53.87(9),  54.94(9),  54.28(8),  54.76(8),  54.45(8),  30.70(8),   0.29(9),  3.64(10),
                 1M =    54.58(8),   0.45(9),
C1    -C2      / 0M =    12.82(9),   3.86(8),   2.43(9),   4.39(9),   3.13(8),   4.11(8),   3.53(8),  22.18(8),  26.01(9),  0.18(10),
                 1M =     3.66(8),  17.59(9),
      -B1      / 0M =     9.95(9),  62.77(8),  64.11(8),  62.27(9),  63.47(8),  62.55(8),  63.10(8),  87.66(8),   8.72(8), 23.70(10),
                 1M =    62.96(7),   0.39(9),
C2    -C3      / 0M =    0.67(10), 55.29(10), 54.72(10), 55.45(10),  55.02(9),  55.31(9),  55.10(9),  32.70(9),  5.20(10),  0.27(11),
                 1M =    55.23(9),  5.07(10),
C3    -C4      / 0M =    14.00(9),  50.73(8),  52.48(9),  50.05(9),  51.64(8),  50.36(8),  51.07(8),  67.65(8),  32.40(8),  0.18(10),
                 1M =    50.99(7),  23.56(9),
C7    -C8      / 0M =    35.73(9),  38.32(9),  39.76(9),  37.74(9),  39.09(8),  37.96(8),  38.56(8),  42.90(8),  50.10(9), 24.41(10),
                 1M =    38.55(8),  44.42(9),
      -C9      / 0M =    11.61(9),  18.73(9),  18.00(9),  18.97(9),  18.37(8),  18.79(8),  18.51(8),   3.89(8),   3.89(9), 19.57(10),
                 1M =    18.64(8),  10.11(9),
      -C10     / 0M =    30.94(9),  53.75(9),  54.04(9),  53.66(9),  53.89(8),  53.77(8),  53.90(8),  65.47(8),  11.58(9), 45.56(10),
                 1M =    53.78(8),  21.38(9),
C11   -C12     / 0M =    52.96(8),   0.60(8),   1.13(8),   1.27(8),   0.31(7),   0.98(7),   0.27(7),  18.21(7),  69.33(8), 37.58(10),
                 1M =     0.34(7),  59.97(9),
      -C24     / 0M =    58.56(8),   1.08(8),   1.68(8),   0.80(8),   1.42(7),   0.86(7),   1.12(7),   5.28(7),  49.09(8), 62.11(10),
                 1M =     1.19(7),  56.91(9),
      -B1      / 0M =     4.67(8),   1.39(8),   2.47(8),   1.01(8),   1.94(7),   1.24(7),   1.67(7),  26.17(7),   9.25(8), 17.55(10),
                 1M =     1.53(7),   0.39(9),
C12   -C13     / 0M =    61.90(9),   0.14(8),   0.84(8),   0.18(9),   0.52(8),   0.10(8),   0.19(8),   4.67(8),  52.53(8), 64.32(10),
                 1M =     0.26(7),  60.43(9),
      -C17     / 0M =     3.55(8),   0.39(8),   0.71(8),   0.78(9),   0.17(8),   0.55(8),   0.10(7),  24.51(8),   9.96(8), 16.20(10),
                 1M =     0.24(7),   1.27(9),
C13   -C14     / 0M =    53.33(9),   1.97(9),   0.24(9),   2.65(9),   1.06(8),   2.35(8),   1.64(8),  16.97(8),  69.12(9), 38.08(11),
                 1M =     1.71(8), 60.06(10),
C14   -C15     / 0M =     3.70(9),   1.62(9),   0.53(9),   2.01(9),   1.07(8),   1.78(9),   1.34(8),  23.22(9),   9.49(9), 16.12(11),
                 1M =     1.48(8),  0.93(10),
C15   -C16     / 0M =   60.85(10),   1.33(9),  0.69(10),  1.62(10),   0.98(9),   1.56(9),   1.29(9),   6.94(9), 50.38(10), 64.81(11),
                 1M =     1.22(9), 58.65(10),
C16   -C17     / 0M =    54.89(9),   1.70(9),   0.02(9),   2.37(9),   0.80(8),   2.08(8),   1.38(8),  16.65(8),  70.95(9), 39.53(10),
                 1M =     1.44(8),  61.79(9),
C17   -C18     / 0M =    60.82(9),   0.94(9),   0.29(9),   1.23(9),   0.58(8),   1.16(8),   0.89(8),   6.49(8),  50.58(9), 64.54(11),
                 1M =     0.82(8),  58.76(9),
C18   -C19     / 0M =    53.62(9),   0.45(9),   2.18(9),   0.22(9),   1.36(8),   0.07(8),   0.78(8),  18.80(8),  70.52(9), 38.12(11),
                 1M =     0.71(8),  60.92(9),
C19   -C20     / 0M =    60.54(9),   0.28(9),   0.94(9),   0.01(9),   0.65(8),   0.06(8),   0.33(8),   5.20(8),  50.98(9), 63.61(11),

                                                                                                 "gliesep200"  PLATON-GEOMETRY  Page   25
=====================================================================================================================================
                 1M =     0.40(8), 58.90(10),
      -C24     / 0M =     3.89(9),   0.55(8),   1.65(8),   0.16(9),   1.11(8),   0.40(8),   0.84(7),  25.43(8),   9.85(8), 16.67(10),
                 1M =     0.70(7),   1.06(9),
C20   -C21     / 0M =   52.94(10),   0.76(9),  2.49(10),  0.08(10),   1.67(9),   0.38(9),   1.09(9),  19.29(9), 69.89(10), 37.45(11),
                 1M =     1.02(9), 60.26(10),
C21   -C22     / 0M =     3.47(9),   0.43(9),   1.53(9),   0.04(9),   0.99(8),   0.27(9),   0.71(8),  25.36(9),  10.25(9), 16.25(11),
                 1M =     0.57(8),  1.48(10),
C22   -C23     / 0M =    58.94(9),   0.51(9),   1.11(9),   0.23(9),   0.84(8),   0.29(8),   0.54(8),   5.76(8),  49.22(9), 62.67(11),
                 1M =     0.61(8), 57.15(10),
C23   -C24     / 0M =    55.14(9),   0.39(8),   2.11(8),   0.28(9),   1.30(8),   0.01(8),   0.72(8),  18.20(8),  72.20(8), 39.59(10),
                 1M =     0.65(7),  62.56(9),

                                                                                                 "gliesep200"  PLATON-GEOMETRY  Page   26
=====================================================================================================================================
Ring Puckering Analysis (Cremer & Pople) - (e.s.d. following Norrestam, Acta Cryst. (1981), A37, 764-765)
-------------------------------------------------------------------------------------------------------------------------------------

                   Symmetrical Forms                                                                      References
                   -----------------                                                                      ----------
6-Membered Rings : C : Chair      - Th = 0.0                                          J.C.A. Boeyens, J.Cryst.Mol.Struct. 8,(1978),317-320
                   H : Half-Chair- Th = 50.8; Phi = k X 60 + 30
                   E : Envelope  - Th = 54.7; Phi = k X 60
                   S : Screw-Boat- Th = 67.5; Phi = k X 60 + 30
                   B : Boat      - Th = 90.0; Phi = k X 60
                   T : Twist-Boat- Th = 90.0; Phi = k X 60 + 30


Definitions (All Values Rounded on Esd)
---------------------------------------
Dev            - Deviation of Atom I from Cremer&Pople Plane (Defined Differently from Least-Squares Plane)
Cs(I),C2(I)    - Mirror Plane and 2-Axis Asym. Par. for Atom I (See Duax et al., Topics in Stereochemistry,V-9, (1976) pp.271-383)
Cs(I-J),C2(I-J)- Asymmetry Parameters for Bond I-J
Tors(I-J)      - Torsion Angle for Bond I-J



Descriptors for Torsion Angles                                        Descriptors for Ring Substituents (J.Appl.Cryst.,1983,16,431)
------------------------------                                        -------------------------------------------------------------

Torsion Angle Range     Full Descriptor     Short Descriptor          Angle Range of Subst.   Full Descriptor     Short Descriptor
-------------------------------------------------------------         -----------------------------------------------------------
    0   TO   30 Deg     +  Syn-Periplanar       +sp                     0    TO  30 Deg.            Axial             ax
   30   to   90         +  Syn-Clinal           +sc                    30    to  60            Bisectional            bi
   90   to  150         + Anti-Clinal           +ac                    60    to  90             Equatorial            eq
  150   to  180         + Anti-Periplanar       +ap
    0   to  -30         -  Syn-Periplanar       -sp
  -30   to  -90         -  Syn-Clinal           -sc
  -90   to -150         - Anti-Clinal           -ac
 -150   to -180         - Anti-Periplanar       -ap


*** NOTE *** - For Ring Puckering Comparisons: Make Sure that the Absolute Configuration, Pivot Atom and Cyclic Sense Agree.
             - The "RING AT1 AT2 AT3 ... ATn" Instruction Gives the User Explicit Choice of Pivot Atom (AT1) and Sense (AT2).
             - Use TRNS Instructions to Obtain the Required Absolute Configuration.
             - The Values of Theta and Phi [ = Phi(2)] Depend on the Abs. Conf. and the Choice of the First and Second Ring Atom.
             - Alternatively, Appropriate Phase Shifts may be Applied to the Same Effect (see Below)
             - C&P Analysis is with reference to the Cremer-Pople plane, not to be confused with the associated LS-plane.
               See H.Essen & D.Cremer (1984) Acta Cryst. B40, 418-420.
```

```
            For Correct Usage of C&P Puckering Parameters see also: D. Cremer (1984) Acta Cryst. B40, 498-500.

                                                                                              "gliesep200"  PLATON-GEOMETRY  Page   27
=========================================================================================================================================
  6-Membered Ring ( 1)   N1     -->   C4     -->   C3     -->   C2     -->   C1     -->   B1     -->
-----------------------------------------------------------------------------------------------------------------------------------------
                              sp2          sp3          sp2          sp2          sp3
Dev (A) from CPplane   0.1605(12)  -0.2494(14)   0.1087(15)   0.1209(15)  -0.2099(15)   0.0692(15)
Cs(I)-Asym-Par (Deg)    37.18(15)     6.80(15)    43.94(14)    37.18(15)     6.80(15)    43.94(14)
C2(I)-Asym-Par (Deg)    31.49(14)    48.25(14)    21.06(16)    31.49(14)    48.25(14)    21.06(16)
Ring Bond Angle(Deg)   117.44(11)   113.90(11)   121.63(12)   120.61(12)   113.29(12)   118.97(12)

Tors(I-J) (Deg)             -36.39(16)    32.76(19)      -0.7(2)    -25.45(18)    20.11(17)    10.54(17)
Cs(I-J)-Asym-Par   (Deg)     33.55(18)    28.27(18)    12.62(17)     33.55(18)    28.27(18)    12.62(17)
C2(I-J)-Asym-Par   (Deg)     21.53(18)    35.84(18)    57.36(18)     21.53(18)    35.84(18)    57.36(18)
Ring Bond Distance (Ang)    1.4962(16)   1.4989(19)     1.328(2)   1.4946(19)     1.586(2)   1.4053(18)

Weighted Average Ring Bond Distance = 1.4692(  8,344) Ang. - NOTE: 1st esd. Internal, 2nd esd External.
Weighted Average Abs. Torsion Angl. =  21.86(  7,537) Deg.   see: e.g. Domenicano et al., Acta Cryst.(1975), B31, 221-234.

Cremer & Pople Puckering Parameters  [D. Cremer & J.A. Pople, J.Amer.Chem.Soc., 97, (1975), 1354-1358]
-----------------------------------------------------------------------------------------------------------------------------------------
Q(2)  =      0.4011(14) Ang.,  Phi(2) =        52.6(2) Deg
Q(3)  =      0.0484(14) Ang.

Puckering Amplitude (Q) =     0.4040(14) Ang, Theta =        83.1(2) Deg,  Phi =        52.6(2) Deg


* NOTE * - A Change of the Absolute Configuration Transforms Theta into 180 - Theta and Phi into 180 + Phi.
         - A Cyclic Forward Shift of the Pivot Atom from At1 to At2 Transforms Theta into 180 - Theta and Phi into Phi + 120.
         - A Change of the Sense Transforms Theta into 180 - Theta and Phi into 180 - Phi, and Vice Versa.

Conformational Analysis (G.G. Evans & J.A. Boeyens, Acta Cryst. (1989), B45, 581-590)

           Coefficients of Primitive and Normalised Forms
           M      Primitive  Coefficient Angular Value
           ------------------------------------------
CosForm    2        0.308        0.670        4.0
SinForm             0.103        0.225        2.0
           3                     0.105        1.0
```

```
                                                                                              "gliesep200"  PLATON-GEOMETRY  Page   28
=========================================================================================================================================
  6-Membered Ring ( 2)   C11    -->   C12    -->   C17    -->   C18    -->   C19    -->   C24    -->
-----------------------------------------------------------------------------------------------------------------------------------------
                              sp2          sp2          sp2          sp2          sp2          sp2
Dev (A) from CPplane  -0.0135(13)   0.0013(13)   0.0112(15)  -0.0116(15)  -0.0006(15)   0.0131(13)
Cs(I)-Asym-Par (Deg)     1.56(15)     3.30(15)     1.74(15)     1.56(15)     3.30(15)     1.74(15)
C2(I)-Asym-Par (Deg)     2.95(15)     0.50(15)     2.85(15)     2.95(15)     0.50(15)     2.85(15)
Ring Bond Angle(Deg)   118.47(11)   120.62(11)   119.46(12)   121.15(12)   119.28(12)   120.97(11)

Tors(I-J) (Deg)               1.46(17)     0.83(19)      -2.1(2)       1.0(2)     1.38(19)    -2.56(17)
Cs(I-J)-Asym-Par   (Deg)      1.33(18)     1.19(19)     2.39(18)     1.33(18)     1.19(19)     2.39(18)
C2(I-J)-Asym-Par   (Deg)      3.44(19)     3.56(19)     0.13(19)     3.44(19)     3.56(19)     0.13(19)
Ring Bond Distance (Ang)    1.4145(17)   1.4395(18)   1.3940(19)   1.3961(19)   1.4369(18)   1.4109(17)

Weighted Average Ring Bond Distance = 1.4159(  7, 77) Ang. - NOTE: 1st esd. Internal, 2nd esd External.
Weighted Average Abs. Torsion Angl. =   1.59(  8, 27) Deg.   see: e.g. Domenicano et al., Acta Cryst.(1975), B31, 221-234.


No C & P - Puckering Analysis since <Tau> =  1.6 <    5.0 Deg.



Centroid Cg2   : x ,y ,z       0.50568(4)      0.77262(5)    0.571257(18)
                XO,YO,ZO        6.7403(5)       8.6043(5)      16.3364(5)
```

```
                                                                                              "gliesep200"  PLATON-GEOMETRY  Page   29
=========================================================================================================================================
  6-Membered Ring ( 3)   C12    -->   C13    -->   C14    -->   C15    -->   C16    -->   C17    -->
-----------------------------------------------------------------------------------------------------------------------------------------
                              sp2          sp2          sp2          sp2          sp2          sp2
Dev (A) from CPplane   0.0126(13)  -0.0084(14)  -0.0030(15)   0.0102(15)  -0.0061(15)  -0.0053(15)
Cs(I)-Asym-Par (Deg)     0.48(16)     2.03(16)     2.51(16)     0.48(16)     2.03(16)     2.51(16)
C2(I)-Asym-Par (Deg)     2.68(15)     1.82(16)     1.06(16)     2.68(15)     1.82(16)     1.06(16)
Ring Bond Angle(Deg)   117.50(11)   121.65(12)   120.36(13)   120.11(13)   121.34(13)   119.00(12)

Tors(I-J) (Deg)              -2.03(18)      0.7(2)       1.2(2)      -1.5(2)       0.0(2)     1.68(18)
Cs(I-J)-Asym-Par   (Deg)      1.89(19)     0.60(19)     1.54(19)     1.89(19)     0.60(19)     1.54(19)
C2(I-J)-Asym-Par   (Deg)        1.1(2)     3.21(19)       2.1(2)       1.1(2)     3.21(19)       2.1(2)
Ring Bond Distance (Ang)    1.4300(18)   1.3647(19)     1.422(2)     1.357(2)   1.4285(19)   1.4395(18)

Weighted Average Ring Bond Distance = 1.4086(  8,146) Ang. - NOTE: 1st esd. Internal, 2nd esd External.
Weighted Average Abs. Torsion Angl. =   1.23(  8, 30) Deg.   see: e.g. Domenicano et al., Acta Cryst.(1975), B31, 221-234.


No C & P - Puckering Analysis since <Tau> =  1.2 <    5.0 Deg.



Centroid Cg3   : x ,y ,z       0.66678(4)      0.78298(5)    0.530518(19)
                XO,YO,ZO        8.8876(5)       8.7197(5)      15.1714(5)
```

```
                                                                                              "gliesep200"  PLATON-GEOMETRY  Page   30
=========================================================================================================================================
  6-Membered Ring ( 4)   C19    -->   C20    -->   C21    -->   C22    -->   C23    -->   C24    -->
-----------------------------------------------------------------------------------------------------------------------------------------
                              sp2          sp2          sp2          sp2          sp2          sp2
Dev (A) from CPplane  -0.0009(15)  -0.0012(16)   0.0008(16)   0.0017(16)  -0.0037(15)   0.0033(13)
Cs(I)-Asym-Par (Deg)     0.60(16)     0.22(16)     0.38(16)     0.60(16)     0.22(16)     0.38(16)
C2(I)-Asym-Par (Deg)     0.69(16)     0.88(16)     0.83(16)     0.69(16)     0.88(16)     0.83(16)
Ring Bond Angle(Deg)   119.13(12)   120.99(13)   120.28(13)   120.48(13)   121.53(13)   117.59(11)

Tors(I-J) (Deg)                 0.1(2)       0.1(2)       0.2(2)      -0.6(2)     0.77(19)    -0.48(19)
Cs(I-J)-Asym-Par   (Deg)        0.7(2)       0.8(2)       0.7(2)       0.7(2)       0.8(2)       0.7(2)
C2(I-J)-Asym-Par   (Deg)        0.6(2)       0.1(2)       0.7(2)       0.6(2)       0.1(2)       0.7(2)
Ring Bond Distance (Ang)      1.430(2)     1.358(2)     1.421(2)   1.3614(19)   1.4317(18)   1.4369(18)

Weighted Average Ring Bond Distance = 1.4077(  8,149) Ang. - NOTE: 1st esd. Internal, 2nd esd External.
Weighted Average Abs. Torsion Angl. =   0.38(  8, 12) Deg.   see: e.g. Domenicano et al., Acta Cryst.(1975), B31, 221-234.


No C & P - Puckering Analysis since <Tau> =  0.4 <    5.0 Deg.
```

Centroid Cg4 : x ,y ,z 0.34257(4) 0.76480(5) 0.61023(2)
XO,YO,ZO 4.5661(6) 8.5172(6) 17.4509(6)

"gliesep200" PLATON-GEOMETRY Page 31

10-Membered Ring (5)	C11 -->	C12 -->	C13 -->	C14 -->	C15 -->	C16 -->	C17 -->	C18 -->
	sp2	sp2	sp2	sp2	sp2	sp2	sp2	sp2
Dev (A) from CPplane	-0.0023(13)	-0.0099(13)	-0.0233(13)	0.0020(14)	0.0291(15)	0.0063(14)	-0.0136(14)	-0.0272(14)
Cs(I)-Asym-Par (Deg)	161.23(10)	1.73(11)	159.56(10)	195.16(9)	195.09(9)	161.23(10)	1.73(11)	159.56(10)
C2(I)-Asym-Par (Deg)	158.26(10)	225.92(8)	159.95(10)	113.82(10)	113.93(11)	158.26(10)	225.92(8)	159.95(10)
Ring Bond Angle(Deg)	118.47(11)	121.88(11)	121.65(12)	120.36(13)	120.11(13)	121.34(13)	121.54(12)	121.15(12)
Tors(I-J) (Deg)	-178.98(11)	178.39(12)	0.7(2)	1.2(2)	-1.5(2)	-179.53(14)	177.49(13)	1.0(2)
Cs(I-J)-Asym-Par (Deg)	124.40(11)	126.15(12)	180.26(11)	1.28(11)	178.38(12)	124.40(11)	126.15(12)	180.26(11)
C2(I-J)-Asym-Par (Deg)	127.26(11)	127.38(12)	176.94(11)	252.58(9)	178.83(12)	127.26(11)	127.38(12)	176.94(11)
Ring Bond Distance (Ang)	1.4145(17)	1.4300(18)	1.3647(19)	1.422(2)	1.357(2)	1.4285(19)	1.3940(19)	1.3961(19)

(Continued)	C19 -->	C24 -->
	sp2	sp2
Dev (A) from CPplane	0.0058(14)	0.0331(13)
Cs(I)-Asym-Par (Deg)	195.16(9)	195.09(9)
C2(I)-Asym-Par (Deg)	113.82(10)	113.93(11)
Ring Bond Angle(Deg)	119.28(12)	120.97(11)
Tors(I-J) (Deg)	1.38(19)	-2.56(17)
Cs(I-J)-Asym-Par (Deg)	1.28(11)	178.38(12)
C2(I-J)-Asym-Par (Deg)	252.58(9)	178.83(12)
Ring Bond Distance (Ang)	1.4369(18)	1.4109(17)

Weighted Average Ring Bond Distance = 1.4067(6, 84) Ang. - NOTE: 1st esd. Internal, 2nd esd External.
Weighted Average Abs. Torsion Angl. = 1.43(5, 23) Deg. see: e.g. Domenicano et al., Acta Cryst.(1975), B31, 221-234.

No C & P - Puckering Analysis since <Tau> = 1.4 < 5.0 Deg.

Centroid Cg5 : x ,y ,z 0.58626(3) 0.77786(4) 0.550795(15)
XO,YO,ZO 7.8144(4) 8.6626(4) 15.7513(4)

"gliesep200" PLATON-GEOMETRY Page 32

10-Membered Ring (6)	C11 -->	C12 -->	C17 -->	C18 -->	C19 -->	C20 -->	C21 -->	C22 -->
	sp2	sp2	sp2	sp2	sp2	sp2	sp2	sp2
Dev (A) from CPplane	-0.0200(13)	0.0052(13)	0.0204(14)	-0.0075(14)	-0.0068(14)	-0.0050(15)	0.0030(15)	0.0081(15)
Cs(I)-Asym-Par (Deg)	160.71(10)	112.54(11)	196.03(9)	160.78(11)	160.22(8)	160.71(10)	112.54(11)	196.03(9)
C2(I)-Asym-Par (Deg)	159.72(10)	196.65(9)	113.62(10)	159.65(11)	160.21(9)	159.72(10)	196.65(9)	113.62(10)
Ring Bond Angle(Deg)	118.47(11)	120.62(11)	119.46(12)	121.15(12)	121.59(12)	120.99(13)	120.28(13)	120.48(13)
Tors(I-J) (Deg)	1.46(17)	0.83(19)	-2.1(2)	-178.58(13)	179.64(14)	0.1(2)	0.2(2)	-0.6(2)
Cs(I-J)-Asym-Par (Deg)	179.01(12)	179.49(10)	179.36(11)	125.82(12)	127.03(11)	179.01(12)	179.49(10)	179.36(11)
C2(I-J)-Asym-Par (Deg)	179.24(12)	178.76(9)	178.89(11)	127.73(12)	126.07(11)	179.24(12)	178.76(9)	178.89(11)
Ring Bond Distance (Ang)	1.4145(17)	1.4395(18)	1.3940(19)	1.3961(19)	1.430(2)	1.358(2)	1.421(2)	1.3614(19)

(Continued)	C23 -->	C24 -->
	sp2	sp2
Dev (A) from CPplane	0.0009(14)	0.0017(13)
Cs(I)-Asym-Par (Deg)	160.78(11)	160.22(8)
C2(I)-Asym-Par (Deg)	159.65(11)	160.21(9)
Ring Bond Angle(Deg)	121.53(13)	121.43(11)
Tors(I-J) (Deg)	179.33(13)	178.93(11)
Cs(I-J)-Asym-Par (Deg)	125.82(12)	127.03(11)
C2(I-J)-Asym-Par (Deg)	127.73(12)	126.07(11)
Ring Bond Distance (Ang)	1.4317(18)	1.4109(17)

Weighted Average Ring Bond Distance = 1.4068(6, 87) Ang. - NOTE: 1st esd. Internal, 2nd esd External.
Weighted Average Abs. Torsion Angl. = 0.91(5, 17) Deg. see: e.g. Domenicano et al., Acta Cryst.(1975), B31, 221-234.

No C & P - Puckering Analysis since <Tau> = 0.9 < 5.0 Deg.

Centroid Cg6 : x ,y ,z 0.42399(3) 0.76874(4) 0.590753(15)
XO,YO,ZO 5.6515(4) 8.5611(4) 16.8939(4)

"gliesep200" PLATON-GEOMETRY Page 33

14-Membered Ring (7)	C11 -->	C12 -->	C13 -->	C14 -->	C15 -->	C16 -->	C17 -->	C18 -->
	sp2	sp2	sp2	sp2	sp2	sp2	sp2	sp2
Dev (A) from CPplane	-0.0283(12)	-0.0201(12)	-0.0260(13)	0.0146(14)	0.0506(15)	0.0212(14)	-0.0147(14)	-0.0352(14)
Cs(I)-Asym-Par (Deg)	135.21(7)	135.52(9)	165.00(9)	213.45(7)	252.77(7)	214.00(8)	191.85(8)	135.21(7)
C2(I)-Asym-Par (Deg)	234.18(7)	234.00(8)	214.24(8)	166.03(8)	96.08(9)	165.32(9)	190.58(8)	234.18(7)
Ring Bond Angle(Deg)	118.47(11)	121.88(11)	121.65(12)	120.36(13)	120.11(13)	121.34(13)	121.54(12)	121.15(12)
Tors(I-J) (Deg)	-178.98(11)	178.39(12)	0.7(2)	1.2(2)	-1.5(2)	-179.53(14)	177.49(13)	-178.58(13)
Cs(I-J)-Asym-Par (Deg)	230.88(8)	178.76(10)	206.80(9)	146.55(8)	145.97(10)	178.22(10)	178.69(8)	230.88(8)
C2(I-J)-Asym-Par (Deg)	103.43(10)	178.86(10)	206.26(9)	252.65(7)	252.99(8)	179.12(10)	179.46(8)	103.43(10)
Ring Bond Distance (Ang)	1.4145(17)	1.4300(18)	1.3647(19)	1.422(2)	1.357(2)	1.4285(19)	1.3940(19)	1.3961(19)

(Continued)	C19 -->	C20 -->	C21 -->	C22 -->	C23 -->	C24 -->
	sp2	sp2	sp2	sp2	sp2	sp2
Dev (A) from CPplane	-0.0178(14)	-0.0083(15)	0.0159(15)	0.0309(15)	0.0168(14)	0.0004(12)
Cs(I)-Asym-Par (Deg)	135.52(9)	165.00(9)	213.45(7)	252.77(7)	214.00(8)	191.85(8)
C2(I)-Asym-Par (Deg)	234.00(8)	214.24(8)	166.03(8)	96.08(9)	165.32(9)	190.58(8)
Ring Bond Angle(Deg)	121.59(12)	120.99(13)	120.28(13)	120.48(13)	121.53(13)	121.43(11)
Tors(I-J) (Deg)	179.64(14)	0.1(2)	0.2(2)	-0.6(2)	179.33(13)	178.93(11)
Cs(I-J)-Asym-Par (Deg)	178.76(10)	206.80(9)	146.55(8)	145.97(10)	178.22(10)	178.69(8)
C2(I-J)-Asym-Par (Deg)	178.86(10)	206.26(9)	252.65(7)	252.99(8)	179.12(10)	179.46(8)
Ring Bond Distance (Ang)	1.430(2)	1.358(2)	1.421(2)	1.3614(19)	1.4317(18)	1.4109(17)

Weighted Average Ring Bond Distance = 1.4024(5, 77) Ang. - NOTE: 1st esd. Internal, 2nd esd External.
Weighted Average Abs. Torsion Angl. = 1.06(4, 17) Deg. see: e.g. Domenicano et al., Acta Cryst.(1975), B31, 221-234.

No C & P - Puckering Analysis since <Tau> = 1.1 < 5.0 Deg.

```
Centroid Cg7   : x ,y ,z       0.50489(3)      0.77359(3)    0.570567(13)
                XO,YO,ZO        6.7298(4)       8.6151(4)      16.3167(4)

                                                                                         "gliesep200"  PLATON-GEOMETRY  Page   34
====================================================================================================================================
Analysis of Short Ring-Interactions with Cg-Cg Distances <   6.0 Ang., Alpha <  20.000 Deg. and Beta < 60.0 Deg.
====================================================================================================================================
- Cg(I)    = Plane number I (= ring number in () above)
- Alpha    = Dihedral Angle between Planes I and J (Deg)
- Beta     = Angle Cg(I)-->Cg(J) or Cg(I)-->Me vector and normal to plane I (Deg)
- Gamma    = Angle Cg(I)-->Cg(J) vector and normal to plane J (Deg)
- Cg-Cg    = Distance between ring Centroids (Ang.)
- CgI_Perp = Perpendicular distance of Cg(I) on ring J (Ang.)
- CgJ_Perp = Perpendicular distance of Cg(J) on ring I (Ang.)
- Slippage = Distance between Cg(I) and Perpendicular Projection of Cg(J) on Ring I (Ang).
- P,Q,R,S  = J-Plane Parameters for Carth. Coord. (Xo, Yo, Zo)

Cg(I) Res(I)   Cg(J)  [   ARU(J)]       Cg-Cg Transformed J-Plane P, Q, R, S     Alpha  Beta Gamma    CgI_Perp    CgJ_Perp  Slippage

Cg2    [ 1] -> Cg3    [  3466.01]   5.4092(8)  0.4612-0.2473-0.8521 -12.3897  53.79(6)  30.4  84.2   0.5497(5)   4.6663(5)
Cg3    [ 1] -> Cg4    [  3566.01]   4.6877(8)  0.4355-0.2207-0.8727  -6.6434  53.32(6)  58.0   7.2   4.6506(5)   2.4807(6)
Cg4    [ 1] -> Cg3    [  3466.01]   4.6876(8)  0.4612-0.2473-0.8521 -12.3897  53.32(6)   7.2  58.0   2.4807(6)   4.6505(5)
Cg4    [ 1] -> Cg5    [  3466.01]   5.7534(7)  0.4529-0.2377-0.8593 -12.4294  52.76(5)  12.8  64.0   2.5226(6)   5.6114(4)
Cg6    [ 1] -> Cg3    [  3466.01]   4.9105(7)  0.4612-0.2473-0.8521 -12.3897  53.48(5)  18.7  72.0   1.5163(4)   4.6500(5)
Cg7    [ 1] -> Cg3    [  3466.01]   5.3911(7)  0.4612-0.2473-0.8521 -12.3897  53.95(5)  30.3  84.2   0.5405(4)   4.6540(5)
                                   ----------                                 ----------------------------------------------
                           Min or Max  4.688                                      52.8   7.2  84.2      -4.651      -5.611

[  3466] = -1/2+X,3/2-Y,1-Z
[  3566] = 1/2+X,3/2-Y,1-Z

                                                                                         "gliesep200"  PLATON-GEOMETRY  Page   35
====================================================================================================================================

Analysis of X-H...Cg(Pi-Ring) Interactions (H..Cg < 3.0 Ang. - Gamma <  40.0 Deg)
====================================================================================================================================
- Cg(J)    = Center of gravity of ring J (Plane number above)
- H-Perp   = Perpendicular distance of H to ring plane J
- Gamma    = Angle between Cg-H vector and ring J normal
- X-H..Cg = X-H-Cg angle (degrees)
- X..Cg    = Distance of X to Cg (Angstrom)
- X-H, Pi = Angle of the X-H bond with the Pi-plane (i.e.' Perpendicular = 90 degrees, Parallel = 0 degrees)

   X--H(I)    Res(I)   Cg(J)  [   ARU(J)]       H..Cg  Transformed J-Plane P, Q, R, S  H-Perp Gamma      X-H..Cg        X..Cg X-H,Pi

C2     -H2     [ 1] -> Cg3    [  5666.01]        2.92  -0.4612-0.2473-0.8521 -14.0868  -2.88   9.73          133   3.6380(15)     51
C4     -H4B    [ 1] -> Cg3    [  7645.01]        2.97  -0.4612 0.2473 0.8521   8.5848   2.87  15.09          167   3.9373(15)     62
C5     -H5A    [ 1] -> Cg2    [  1555.01]        2.86   0.4431 0.2277 0.8671  19.1106   2.60  24.74          120   3.4529(15)     29
C5     -H5A    [ 1] -> Cg5    [  1555.01]        2.91   0.4529 0.2377 0.8593  19.1331   2.60  26.70          142   3.7305(15)     28
C5     -H5A    [ 1] -> Cg7    [  1555.01]        2.88   0.4457 0.2327 0.8644  19.1084   2.61  24.98          120   3.4772(15)     29
                                           ----------                                 ----------------------------------------------
                                   Min or Max  2.860                                  -2.881    9.7       167.00         3.453  62.00

[  5666] = 1-X,1-Y,1-Z
[  7645] = 3/2-X,-1/2+Y,Z
[  1555] = X,Y,Z

The Cg(I) refer to the Ring Centre-of-Gravity numbers given in () in the Ring-Analysis above

Cg(I)           x               y               z                    Xo              Yo              Zo

Cg2         0.50568(4)      0.77262(5)    0.571257(18)             6.7403(5)       8.6043(5)      16.3364(5)
Cg3         0.66678(4)      0.78298(5)    0.530518(19)             8.8876(5)       8.7197(5)      15.1714(5)
Cg4         0.34257(4)      0.76480(5)      0.61023(2)             4.5661(6)       8.5172(6)      17.4509(6)
Cg5         0.58626(3)      0.77786(4)    0.550795(15)             7.8144(4)       8.6626(4)      15.7513(4)
Cg6         0.42399(3)      0.76874(4)    0.590753(15)             5.6515(4)       8.5611(4)      16.8939(4)
Cg7         0.50489(3)      0.77359(3)    0.570567(13)             6.7298(4)       8.6151(4)      16.3167(4)
:: SPF File spf on :GlieseP200207_0ma_pl.spf
```

Computationally Optimized Geometry

The data is provided in the .xyz format.

```
57

C      1.2481957546  -0.5893021259 3.6919235361
C      2.3892740677  -0.9317315809 3.0939373824
C      2.4794810413  -1.3036730299 1.6389173446
N      1.3940644704  -0.7647445032 0.7665973256
B      0.1412199976  -0.4567134455 1.3502616010
C      -0.0470994723 -0.5470421472 2.9359003229
C      -1.1649180534 -0.0392960411 0.5422832744
C      -2.0471742278 -1.0349949009 0.0560116245
C      -3.2858125967 -0.6686711888 -0.5944438067
C      -3.6117819835 0.6841750732  -0.7310103257
C      -2.7753047010 1.6889240884  -0.2349951475
C      -1.5394677017 1.3201410789  0.4187534977
C      -1.7558690065 -2.4338452636 0.1791792150
C      -2.6100919459 -3.3930298439 -0.2999442853
C      -3.8256688878 -3.0216908252 -0.9430831452
C      -4.1490188879 -1.6988030124 -1.0842198990
C      -3.1141359819 3.0733905386  -0.3544233700
C      -2.2962999555 4.0492066728  0.1495990713
C      -1.0834740866 3.6950273615  0.8073999950
C      -0.7209704951 2.3787441860  0.9331586628
Si     1.9203970732  -0.6551603910 -0.9579830372
C      3.2277350569  0.7579496247  -1.1983392688
C      2.6834235043  -2.3293943833 -1.4176569963
C      0.4858355092  -0.3752659375 -2.1480514138
C      4.6668746388  0.3339657071  -0.8283928307
C      3.2382657559  1.1529403757  -2.6961463426
C      2.8433327678  2.0041231670  -0.3732475962
H      1.2497269459  -0.3354587793 4.7520148738
H      3.3212927498  -0.9848017771 3.6545162886
H      2.4945497821  -2.4016996587 1.5425455298
H      3.4509403182  -0.9707413466 1.2592675026
H      -0.6523456757 -1.4432347077 3.1639067563
H      -0.6778404941 0.2856366480  3.2794822443
H      -4.5415996979 0.9611840886  -1.2244015236
H      -0.8301838890 -2.7337304999 0.6632730926
H      -2.3628447712 -4.4456928704 -0.1899785599
H      -4.4924981266 -3.7932142093 -1.3187384385
H      -5.0751175981 -1.4042786467 -1.5729398042
H      -4.0444951573 3.3349017865  -0.8537887443
H      -2.5690873494 5.0968011798  0.0534915621
H      -0.4451015961 4.4763855388  1.2110674253
H      0.2070775479  2.1221612688  1.4366501534
H      3.5501469689  -2.6134871834 -0.8110301779
H      3.0093241077  -2.3172426283 -2.4653069443
H      1.9324903717  -3.1228809831 -1.3197334430
H      -0.0080599852 0.5926284380  -2.0289080496
H      -0.2727577986 -1.1543814923 -2.0407734248
H      0.8778725666  -0.4294029196 -3.1707620064
H      4.9838687688  -0.5656688601 -1.3688456481
H      4.7919092168  0.1432687982  0.2436981663
H      5.3724004489  1.1364056401  -1.0894558930
H      3.4791732263  0.3048043190  -3.3502543363
H      4.0023622243  1.9235509822  -2.8723665664
H      2.2775074288  1.5651876803  -3.0217097976
H      2.8710349824  1.8061234012  0.7047637014
H      1.8370495160  2.3637515281  -0.6181438176
H      3.5435547722  2.8278868445  -0.5764460623
```

References

Becke, A. D. (1993). *J. Chem. Phys.* **98**, 5648–5652.

Epifanovsky, E., *et al.* (2021). *J. Chem. Phys.* **155**, 084801.

Francl, M. M., Pietro, W. J., Hehre, W. J., Binkley, J. S., Gordon, M. S., DeFrees, D. J. & Pople, J. A. (1982). *The Journal of Chemical Physics* **77**, 3654–3665.

Glendening, E. D., Badenhoop, J. K., Reed, A. E., Carpenter, J. E., Bohmann, J. A., Morales, C. M. & Weinhold, F. (2001). *NBO 5.0 University of Wisconsin, Madison, WI: Theoretical Chemistry Institute*.

Hariharan, P. C. & Pople, J. A. (1973). *Theoret. Chim. Acta* **28**, 213–222.

Hehre, W. J., Ditchfield, R. & Pople, J. A. (1972). *The Journal of Chemical Physics* **56**, 2257–2261.

Stephens, P. J., Devlin, F. J., Chabalowski, C. F. & Frisch, M. J. (1994). *J. Phys. Chem.* **98**, 11623–11627.
